# Hydrative Aminoxylation of Ynamides: One Reaction, Two Mechanisms

**DOI:** 10.1002/chem.201706063

**Published:** 2018-01-24

**Authors:** Alexandre Pinto, Daniel Kaiser, Boris Maryasin, Giovanni Di Mauro, Leticia González, Nuno Maulide

**Affiliations:** ^1^ Institute of Organic Chemistry University of Vienna Währinger Strasse 38 1090 Vienna Austria; ^2^ Institute of Theoretical Chemistry University of Vienna Währinger Strasse 17 1090 Vienna Austria

**Keywords:** aminoxylation, density functional calculations, heterocycles, radicals, reaction mechanisms

## Abstract

Organic synthesis boasts a wide array of reactions involving either radical species or ionic intermediates. The combination of radical and polar species, however, has not been explored to a comparable extent. Herein we present the hydrative aminoxylation of ynamides, a reaction which can proceed by either a polar‐radical crossover mechanism or through a rare cationic activation. Common to both processes is the versatility of the persistent radical TEMPO and its oxidised oxoammonium derivative TEMPO^+^. The unique mechanisms of these processes are elucidated experimentally and by in‐depth DFT‐calculations.

## Introduction

**Scheme 1:**
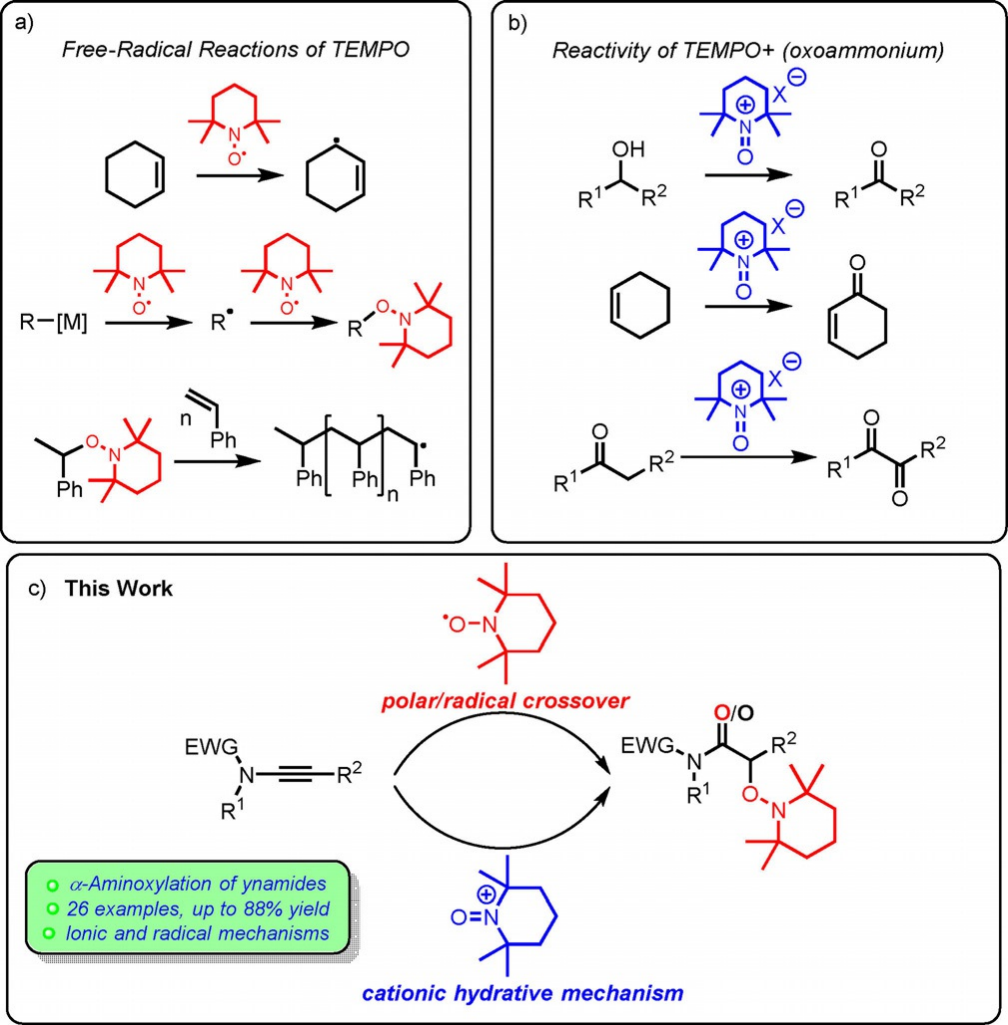
a) Reactivity patterns of TEMPO, b) TEMPO^+^, and c) our hydrative aminoxylation.

The chemistry of free radicals has shaped organic chemistry for over a hundred years, and during this time, has produced several ground‐breaking innovations.^1^ Once thought uncontrollable due to high reactivity and barely predictable behaviour, the last decades have brought a deeper understanding of the role of free radicals in organic reactions^2^ and have placed them at the forefront of some major developments in organic synthesis. These range from free‐radical chain reactions,^3^ all the way to photoredox catalysis.^4^ In contrast to transient radicals generated in situ, persistent radicals exhibit much greater lifetimes and therefore stability.^5^ The triphenylmethyl radical, the first described member of this family,^6^ is perhaps the most well‐known carbon‐centred radical, while the oxygen‐centred persistent nitroxyl radical (2,2,6,6‐tetramethylpiperidin‐1‐yl)oxyl (TEMPO) has also gained widespread attention since its discovery in 1959.^7^ TEMPO (alongside other nitroxyl radicals) has found extensive application in hydrogen‐abstraction reactions,^8^ as well as in combination with organometallic reagents for C−O, C−N and also C−C coupling reactions,7c,7d and in nitroxide‐mediated living free‐radical polymerisation (NMP)7a, 9 (Scheme 1 a). Additionally, its longevity allows TEMPO to be used as a trapping agent or radical scavenger in radical carbon–carbon bond‐forming reactions.7c, 10 Similarly, TEMPO itself has been employed in the aminoxylation of enolate derivatives, affording α‐oxidised carbonyl products.^11^ It is, however, arguably most famous for its ability to oxidise primary and secondary alcohols to the corresponding carbonyl compounds,^12^ a task which it achieves via its oxidised oxoammonium counterpart TEMPO^+^. Curiously, the chemistry of oxoammonium salts is not much developed beyond this synthetically useful reactivity manifold (Scheme 1 b).^13^

Herein we report the TEMPO‐mediated hydrative aminoxylation of ynamides (Scheme 1 c), an unusual reaction that can proceed by either a polar‐radical crossover mechanism or a cationic hydrative pathway and which showcases the unique versatility of the chemistries of persistent radicals and keteniminium ions, as well as a detailed mechanistic and computational study of the process.

## Results and Discussion

**Scheme 2:**
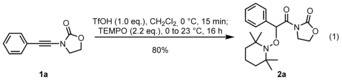
Coupling of **1 a** with TEMPO following preactivation with a Brønsted acid.

Following our recent report on the reaction of TEMPO with activated amides,^14^ our initial efforts focused on the combination of ynamides^15^ with TEMPO under the action of a Brønsted acid (Scheme 2). We eventually found that it was possible to intercept an acid‐preactivated ynamide **1 a** with TEMPO under mild conditions. This enabled the preparation of a hydrative oxyamination product **2 a** in 80 % isolated yield.^16^ Further modification of the conditions, including the premixing of ynamide and TEMPO, as well as the use of fewer equivalents of TEMPO, led to no improvement in yield (details of optimisation experiments are compiled in the Supporting Information). The strict requirement for 2.2 equivalents of TEMPO in order to obtain high yields of product would prove to have significant mechanistic implications (vide infra).

**Scheme 3:**
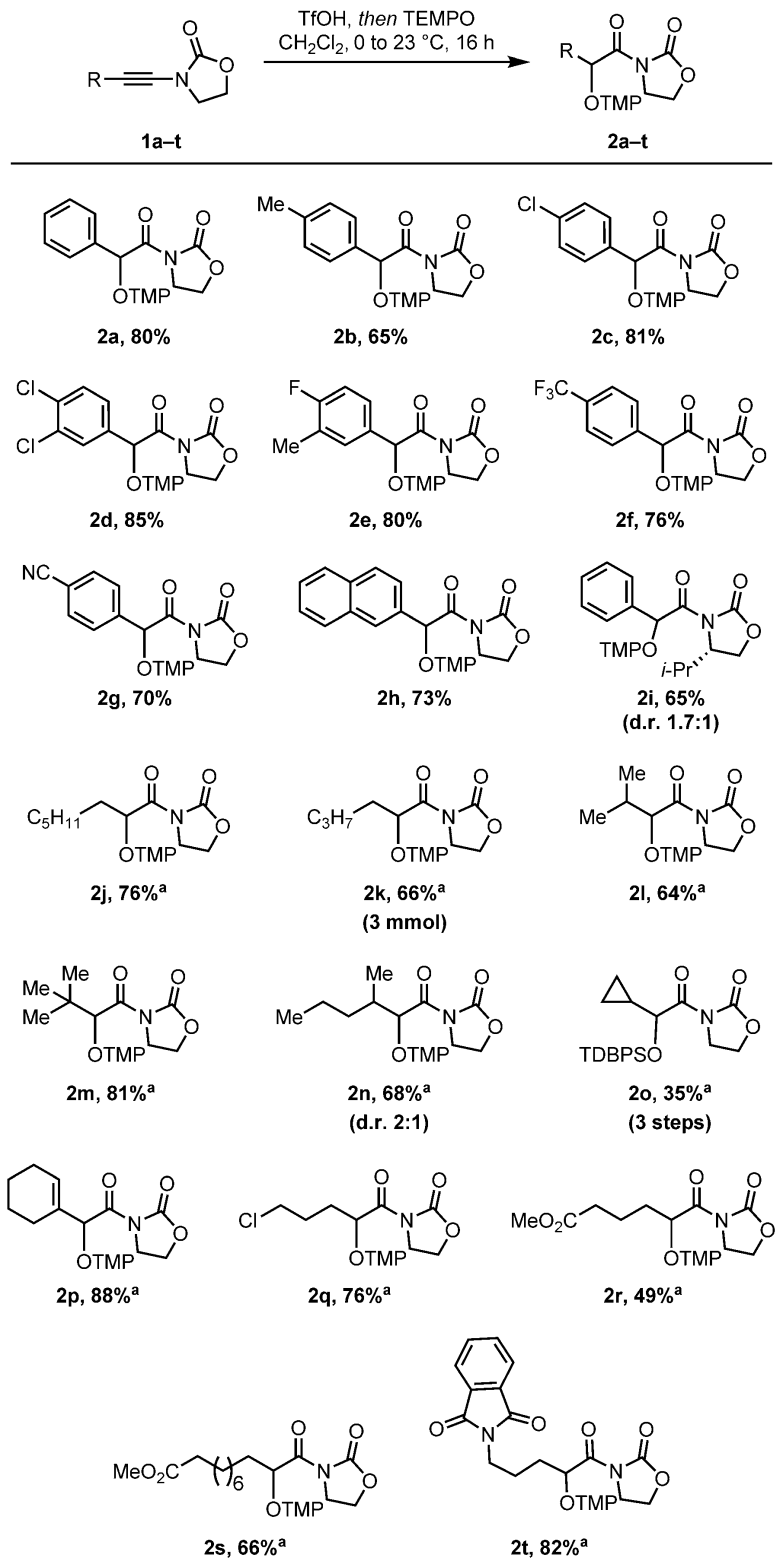
Scope of the aminoxylation reaction of ynamides with TEMPO. Yields refer to isolated products. For details, see the Supporting Information. [a] Reaction performed at 40 °C.

Having identified optimal reaction conditions, we were interested in investigating the scope and functional group tolerance of the reaction (Scheme 3).

Aromatic substituents on the ynamide were well tolerated throughout (**2 a**–**i**), affording the desired hydrative aminoxylation products in high yields. Upon changing the substitution to aliphatic chains (**2 j**–**t**), it became evident that the reaction benefited from slightly elevated temperatures (40 °C). When these conditions were applied, linear (**2 j, k**) and branched (**2 l**–**n**) aliphatic substrates alike smoothly underwent hydrative aminoxylation and good to excellent yields of the corresponding products were isolated. For both aromatic and aliphatic substrates, poor diastereocontrol was observed (**2 i** and **2 n**, respectively). Cyclopropyl ynamide **1 o**, a valuable substrate to probe radical processes (vide infra), also underwent the reaction with minimal ring cleavage.^17^ However, isolation of the corresponding product **2 o** required reductive TMP‐cleavage and TBDPS‐protection, contributing to the modest 35 % yield. Various functional groups were tolerated under the reaction conditions, including alkene (**2 p**), chloride (**2 q**), ester (**2 r**,^18^ **2 s**) and phthalimide (**2 t**) moieties.

**Scheme 4:**
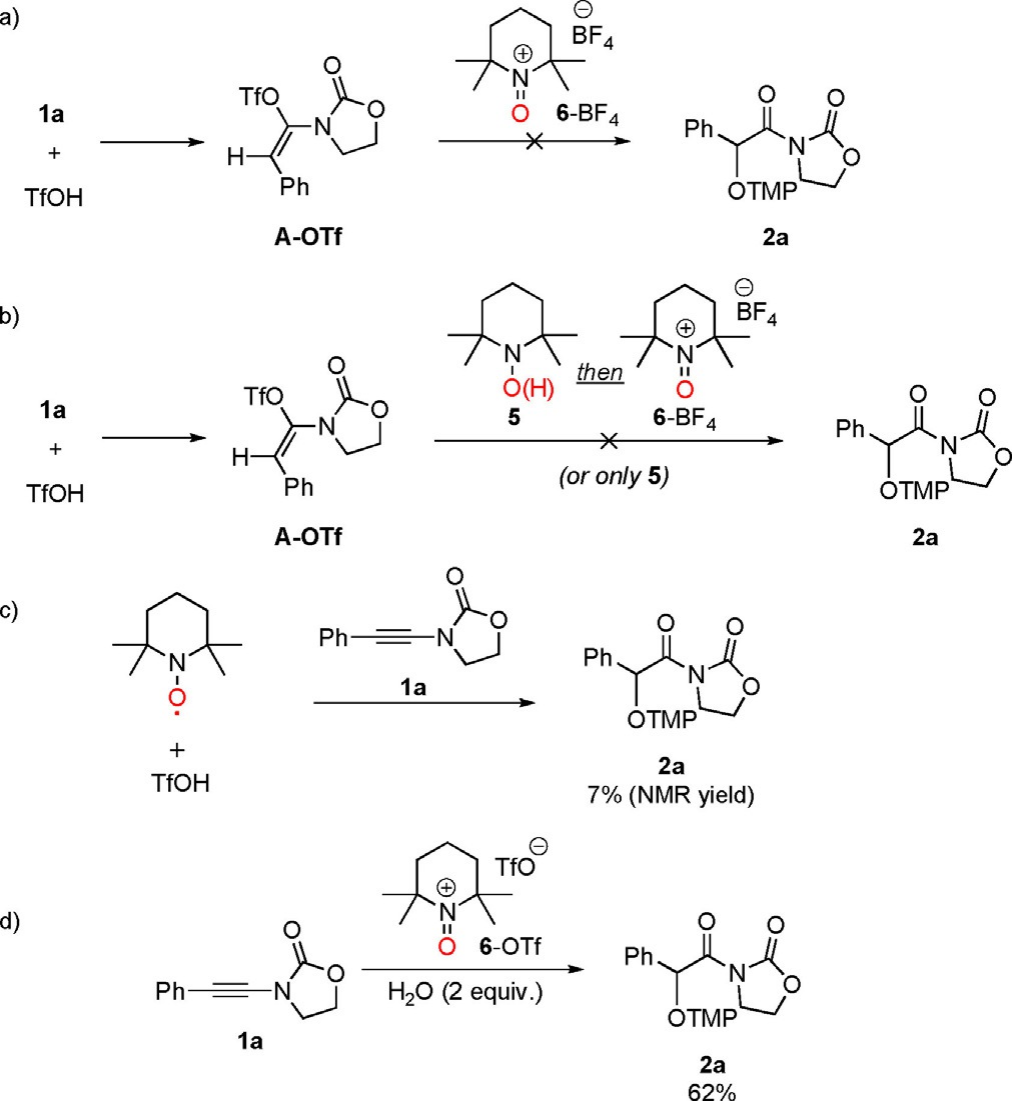
Mechanistic experiments.

From the outset, we were intrigued about the role of TEMPO in this reaction, and carried out the mechanistic experiments depicted in Scheme 4.^19^, ^20^, ^21^ For instance, we initially suspected that TEMPO^+^, the oxoammonium counterpart of TEMPO, was involved in this process. However, substituting TEMPO for TEMPO^+^ in the procedure presented above yielded no traces of product (Scheme 4 a). Similar thoughts concerning a possible in situ disproportionation of TEMPO under reaction conditions led us to add TEMPO‐H (the reduced, protonated form of TEMPO) instead, which also did not afford any product (with or without added TEMPO^+^, Scheme 4 b).

Similarly, the addition of triflic acid to TEMPO (known to promote disproportionation^22^) followed by subsequent introduction of the ynamide into the reaction mixture afforded only 7 % NMR yield of the hydrative aminoxylation product (Scheme 4 c).

Surprisingly, however, we observed that in the absence of triflic acid, a combination of TEMPO^+^ (1.00 equiv) and water (2.00 equiv) is competent in providing the hydrative aminoxylation product **2 a** in 62 % yield (Scheme 4 d). This unexpected observation hints at the ability of TEMPO^+^ to activate ynamides as a cationic O^+^‐donor reagent.^23^

**Scheme 5:**
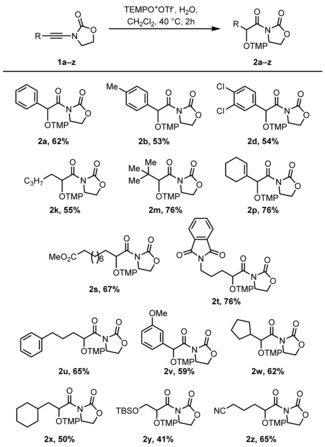
Scope of the electrophilic TEMPO^+^/H_2_O addition to ynamides. Yields refer to isolated products.

The generality of this transformation was briefly investigated and results are compiled in Scheme 5. As can be seen, the use of TEMPO^+^/water allows hydrative aminoxylation of several ynamides in yields comparable to those of the combined TfOH/TEMPO procedure (and with considerably shorter reaction times) for a variety of substitution patterns. In addition to select repeated examples from Scheme 3 (**2 a**,**b**,**d**,**k**,**m**,**p**,**s**,**t**), ynamides containing varying alkyl and aryl substitution (**2 u**–**x**) were smoothly converted to the desired products and, pleasingly, both silyl ethers (**2 y**) and nitriles (**2 z**) were tolerated under the reaction conditions.

### Mechanistic studies

**Scheme 6:**
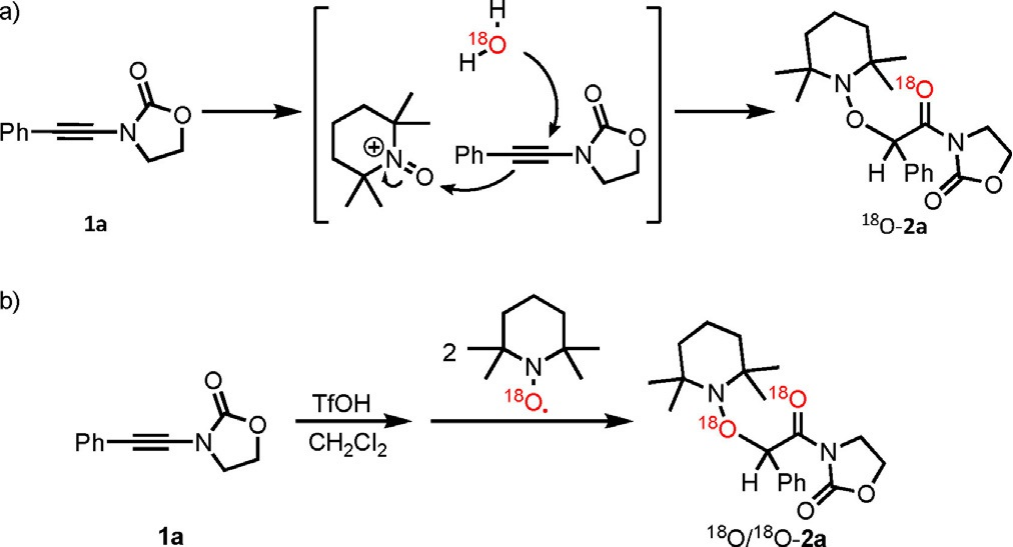
a) Proposed mechanistic outline and isotopic labelling validation for the TEMPO^+^‐mediated hydrative aminoxylation of ynamides. b) Double incorporation of the ^18^O‐label for the reaction with TEMPO.

The unusual observation that two sets of diametrically opposed conditions lead to the same product, raises significant mechanistic questions. While the procedure involving TEMPO^+^/water appears to proceed by a “conventional” cationic activation/aqueous capture pathway (Scheme 6 a), we still had no clear picture for the intriguing polar‐radical combination of the TfOH/TEMPO protocol. In particular, the stringent requirement for 2 equivalents of TEMPO in the latter set of conditions contrasts with the successful hydrative aminoxylation observed with only 1 equivalent TEMPO^+^ in the aqueous procedure. Furthermore, the possibility that both mechanisms would overlap remained open—until the isotopic labelling experiments of Scheme 6 were carried out.

As shown, when using ^18^O‐water in conjunction with TEMPO^+^, unambiguous incorporation of the label into the carbonyl oxygen was observed (Scheme 6 a). This strongly suggests that the reaction proceeds by cationic activation of the ynamide coupled to hydrolysis. Unexpectedly, the use of ^18^O‐labelled TEMPO for the TfOH/TEMPO procedure led to significant double incorporation of the label (Scheme 6 b), indicating that both oxygens inserted into the final product originate from the persistent aminoxyl radical reagent. Notably, quenching the TfOH/TEMPO reaction with ^18^O‐labelled water did not lead to incorporation of the label into the final product, thereby further corroborating a difference in mechanism for the two transformations. At this juncture, we resorted to quantum chemical calculations at the DFT level of theory (see the Supporting Information for the computational details) to shed more light on the intricacies of this unusual polar‐radical crossover process.

### DFT studies

**Scheme 7:**
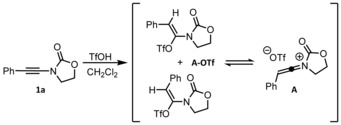
Established characteristics on the reaction of ynamides with TfOH.

As shown previously,^24^ the treatment of ynamide **1 a** with TfOH leads to the transient formation of an *E*/*Z*‐mixture of the triflated species **A‐OTf**, which exists in equilibrium with the keteniminium ion **A**. For this reason, calculations of the reaction mechanism for the formation of products **2 a** were performed starting from **A** (Scheme 7).

At the outset of our calculations, we were mindful of the labelling studies that effectively established the prerequisite for incorporation of two oxygen atoms from TEMPO in the final product; this prerequisite was reflected in the calculations.

**Figure 1:**
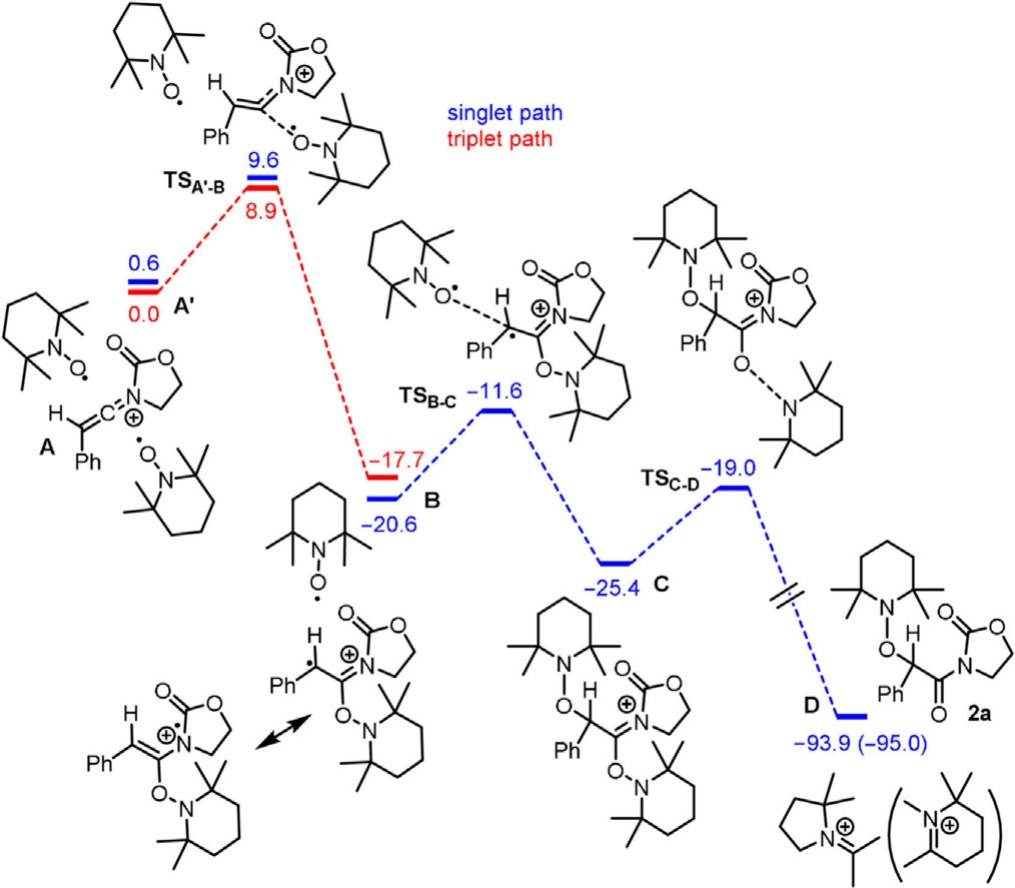
The computed reaction pathway (Δ*G*_298,DCM_) for the conversion of the intermediate **A** into the final product **D/2 a**. The energy of the intermediate **A′** is taken as a reference (0.0 kcal mol^−1^).

The computed reaction profile is shown in Figure 1. The starting point (intermediate **A′**) presents two TEMPO radicals and the keteniminium cation **A**. In the first step, one of the TEMPO radicals attacks the cation **A** leading to the imidate intermediate **B** (Pinner‐type).^25^ The intermediates **A′** and **B**, as well as the corresponding transition state **TS_A′‐B_** are cationic diradicals and therefore exist both in triplet and singlet states, both of which were considered in the calculations (shown in red and blue, Figure 1).^26^ Intersystem crossing (ISC) of the triplet and the singlet states occurs on the phase between the transition state **TS_A′‐B_** and the intermediate **B** (Figure 1). The depicted resonance structure of **B** (a *N,O*‐ketene acetal derivative) also accounts for the stability of the cationic radical species and thereby can be reconciled with the low amounts of the corresponding ring‐opening product detected in the case of cyclopropyl product **2 o** (vide supra).

The next steps of the reaction occur in the closed‐shell state. Intermediate **B** and the second TEMPO radical recombine, forming intermediate **C**. This explains the experimental fact that both oxygen atoms within the final product are derived from TEMPO (cf. Scheme 6 b). The final step **C→D** is very favourable thermodynamically (−95.0 kcal mol^−1^). In this step, one of the O−N bonds is cleaved heterolytically, leading to the neutral final product **2 a**, and a cation originating from rearrangement of the TMP^+^ fragment (two possible cations are depicted in Figure 1).^27^

We have also computationally considered the reaction of the keteniminium intermediate with TEMPOH or TEMPO^−^ as alternative non‐radical mechanism of the studied processes.

The quantum chemical calculations at the DFT level of theory suggest that the necessary closed‐shell (non‐radical) transition state does not exist as a stationary point on the potential energy surface. This means that this process has a large kinetic barrier and therefore is highly unlikely to take place. This is consistent with the aforementioned experimental observations (cf. Scheme 4).

## Conclusions

In conclusion, we have documented an unusual hydrative aminoxylation of ynamides that can proceed by two different mechanisms. Crucial to each pathway is the presence of either the persistent aminoxyl radical TEMPO or its oxidised oxoammonium variant TEMPO^+^. The first process involves addition of a radical species to a keteniminium intermediate, a transformation which is underrepresented in synthesis and which was elucidated by extensive DFT calculations. The second pathway dispenses with acidic pre‐activation and proceeds by a classical nucleophile/electrophile cooperative process. Both processes highlight the versatility of TEMPO as a truly chameleonic reagent in synthesis.

## Conflict of interest

The authors declare no conflict of interest.

## Supporting information

As a service to our authors and readers, this journal provides supporting information supplied by the authors. Such materials are peer reviewed and may be re‐organized for online delivery, but are not copy‐edited or typeset. Technical support issues arising from supporting information (other than missing files) should be addressed to the authors.

Supplementary
