# Supplementary material for: Hydrative Aminoxylation of Ynamides: One Reaction, Two Mechanisms
Source: Chemistry. 2018 Jan 24;24(10):2515–9. doi: 10.1002/chem.201706063 (PMC5838720; doi:10.1002/chem.201706063)
Supplement: Supplementary file 1 — Supplementary [file CHEM-24-2515-s001.pdf]

CHEMISTRY
A European Journal

Supporting Information

Hydrative Aminoxylation of Ynamides: One Reaction, Two Mechanisms

Alexandre Pinto+,[a] Daniel Kaiser+,[a] Boris Maryasin+,[a, b] Giovanni Di Mauro,[a]
Leticia González,[b] and Nuno Maulide*[a]

chem_201706063_sm_miscellaneous_information.pdf

Supporting Information

Table of Contents

1. General Information 2
2. Optimization 3
3. Ynamides 4
   3.1. General Procedure 4
   3.2. Characterization 5
4. Experimental Procedure and Characterization of Products 11
   4.1. General Procedures TEMPO 11
   4.2. General Procedure $TEMPO^+$ 12
   4.3. Characterization of Products 13
   4.4. Products of Reductive (3) and Oxidative (4) Cleavage 26
5. Mechanistic Experiments 27
6. NMR-Spectra 31
   6.1. Ynamides 31
   6.2. Products 35
   6.3. Derivatives 61
7. DFT-Calculations 63

1. General Information

Unless otherwise stated, all glassware was flame-dried before use and all reactions were performed under an atmosphere of argon. All reagents were used as received from commercial suppliers unless otherwise stated. Reaction progress was monitored by thin layer chromatography (TLC) performed on aluminium plates coated with silica gel F_{254} with 0.2 mm thickness. Chromatograms were visualized by fluorescence quenching with UV light at 254 nm or by staining using potassium permanganate. Flash column chromatography was performed using silica gel 60 (230-400 mesh, Merck and co.). Neat infra-red spectra were recorded using a Perkin-Elmer Spectrum 100 FT-IR spectrometer. Wavenumbers (**ν_{max}**) are reported in cm^{-1}. Mass spectra were obtained using a Finnigan MAT 8200 or (70 eV) or an Agilent 5973 (70 eV) spectrometer, using electrospray ionization (ESI). All ^{1}H-NMR and ^{13}C-NMR spectra were recorded using a Bruker AV-400 or AV-600 spectrometer at 300K. Chemical shifts were given in parts per million (ppm, δ), referenced to the solvent peak of $CDCl_3$, defined at δ = 7.26 ppm (^{1}H NMR) and δ = 77.00 (^{13}C NMR), or C_6D_6, defined at δ = 7.16 ppm (^{1}H NMR) and δ = 128.06 (^{13}C NMR). Coupling constants are quoted in Hz (*J*). ^{1}H NMR splitting patterns were designated as singlet (s), doublet (d), triplet (t), quartet (q), pentet (p). Splitting patterns that could not be interpreted or easily visualized were designated as multiplet (m) or broad (br).

2. Optimization

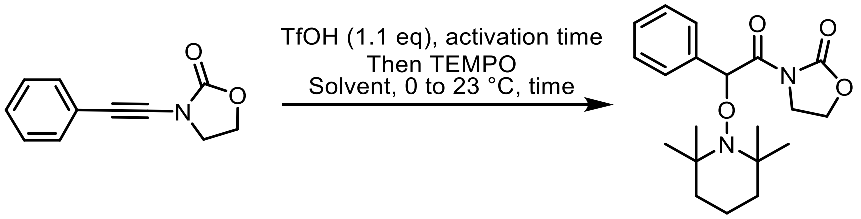

Solvent/[M]	*TEMPO (eq)*	*Activation Time*	*Reaction Time (h)*	*Yield (%)*
CH_2Cl_2 [0.05M]	2.2	15min	16	59%
**CH_2Cl_2 [0.1M]**	**2.2**	**15min**	**16**	**80%**
CH_2Cl_2 [0.1M]	1.5	15min	16	30%[a)]
CH_2Cl_2 [0.1M]	2.2	15min	10	42%
CH_2Cl_2 [0.2M]	2.2	15min	16	73%
CH_2Cl_2 [0.5M]	2.2	15min	16	50%
CH_2Cl_2 [0.1M]	2.2	15min	16	20%[b)]
DCE [0.1M]	2.2	15min	18	53%
$PhCF_3$ [0.1M]	2.2	15min	16	65%
THF [0.1M]	2.2	15min	16	n.d.
MeCN [0.1M]	2.2	15min	16	n.d.

DCE = 1,2-dichloroethane; THF = tetrahydrofuran; [a)]^{1}H-NMR yield, using 1,3,5-trimethoxybenzene as internal standard. [b)]TfOH was added to a mixture of substrate and TEMPO.

3. Ynamides

3.1. General Procedure

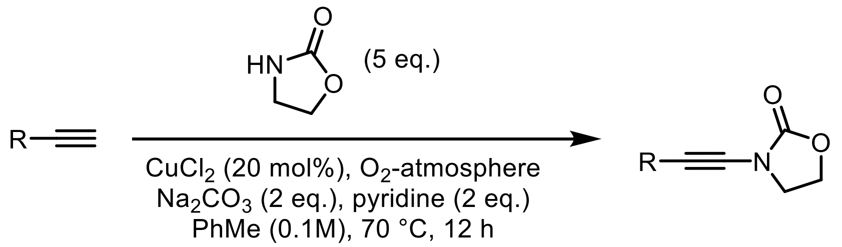

All ynamides were prepared following the method developed by Stahl.[1] $CuCl_2$ (20 mol%), 2-oxazolidone (5.0 eq) and Na_2CO_3 (2.0 eq) were added to a round bottom flask. The flask was subsequently purged with oxygen for 15 min, after which a solution of pyridine (2.0 eq) in dry toluene (0.2 M) was added. A balloon filled with oxygen was connected to the flask and the flask was heated at 70 °C. After 15 min, a solution of the corresponding alkyne (1.0 eq.) in dry toluene (0.2 M) was added over the course of 4 h using syringe pump addition. After completion of the addition, the mixture was stirred at 70 °C for another 12 h and was then cooled to ambient temperature (23 °C). The reaction mixture was concentrated under reduced pressure and the residue was purified by flash chromatography on silica gel with hexane/ethyl acetate.

[1] T. Hamada, X. Ye, S. S. Stahl, *J. Am. Chem. Soc.* **2008**, *130*, 833–835.

3.2. Characterization

3-(Phenylethynyl)oxazolidin-2-one (1a)

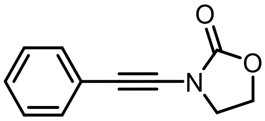 Prepared following the general procedure. All spectroscopic data were in good accordance with those reported in the literature.[2]

3-(*p*-Tolylethynyl)oxazolidin-2-one (1b)

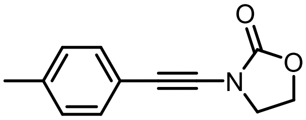 Prepared following the general procedure. All spectroscopic data were in good accordance with those reported in the literature.[3]

3-((4-Chlorophenyl)ethynyl)oxazolidin-2-one (1c)

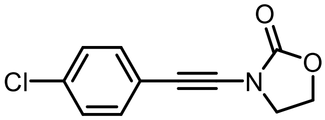 Prepared following the general procedure. All spectroscopic data were in good accordance with those reported in the literature.[3]

3-((3,4-Dichlorophenyl)ethynyl)oxazolidin-2-one (1d)

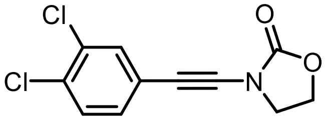 Prepared following the general procedure. All spectroscopic data were in good accordance with those reported in the literature.[4]

3-((4-Fluoro-3-methylphenyl)ethynyl)oxazolidin-2-one (1e)

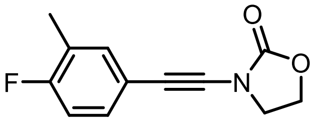 Prepared following the general procedure; (85%, colorless oil). **^{1}H-NMR (400 MHz, $CDCl_3$):** δ 7.30-7.26 (m, 1H), 7.25-7.19 (m, 1H), 6.93 (t, *J* = 8.9 Hz, 1H), 4.50-4.44 (m, 2H), 4.01-3.95 (m, 2H), 2.23 (d, *J* = 2.0 Hz, 3H); **^{13}C-NMR (100 MHz, $CDCl_3$):** δ 161.1 (d, *J* = 248 Hz), 155.9, 134.9 (d, *J* =

[2] B. Peng, X. Huang, L.-G. Xie, N. Maulide, *Angew. Chem. Int. Ed*. **2014**, *53*, 8718–8721.
[3] L.-G. Xie, S. Shaaban, X. Chen, N. Maulide, *Angew. Chem. Int. Ed*. **2016**, *55*, 12864–12867.
[4] H. Huang, J. Fan, G. He, Z. Yang, X. Jin, Q. Liu, H. Zhu, *Chem. Eur. J*. **2016**, *22*, 2532–2538.

5.5 Hz), 130.9 (d, J = 8.3 Hz), 125.1 (d, J = 18 Hz), 117.7 (d, J = 3.8 Hz), 115.1 (d, J = 23 Hz), 78.2, 70.3, 63.0, 46.9, 14.3 (d, J = 3.5 Hz); **IR (neat) ν_{max}:** 2260, 1755, 1433, 1396, 1219, 972, 745, 708; **HRMS (ESI+):** exact mass calculated for $[M+Na]^+$ ($C_{12}H_{10}NFO_2Na$) requires m/z 242.0588, found m/z 242.0584.

3-((4-(Trifluoromethyl)phenyl)ethynyl)oxazolidin-2-one (1f)

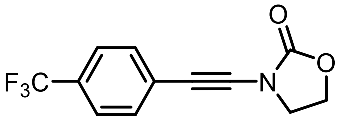

Prepared following the general procedure. All spectroscopic data were in good accordance with those reported in the literature.[3]

4-((2-Oxooxazolidin-3-yl)ethynyl)benzonitrile (1g)

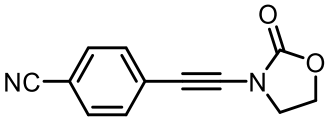

Prepared following the general procedure; (31%, colorless oil). **^{1}H-NMR (600 MHz, CDCl$_3$):** δ 7.61-7.57 (m, 2H), 7.51-7.46 (m, 2H), 4.54-4.50 (m, 2H), 4.06-4.01 (m, 2H); **^{13}C-NMR (150 MHz, CDCl$_3$):** δ 155.4, 132.0 (2C), 131.4 (2C), 127.4, 118.4, 111.2, 83.4, 70.5, 63.2, 46.8; **IR (neat) ν_{max}:** 2965, 2930, 2235, 1768, 1421, 1204; **HRMS (ESI+):** exact mass calculated for $[M+Na]^+$ ($C_{12}H_8N_2O_2Na$) requires m/z 235.0478, found m/z 235.0473.

3-(Naphthalen-2-ylethynyl)oxazolidin-2-one (1h)

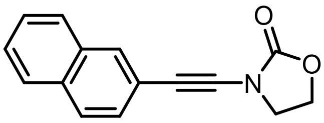

Prepared following the general procedure. All spectroscopic data were in good accordance with those reported in the literature.[5]

***(S)*-4-*Iso*propyl-3-(phenylethynyl)oxazolidin-2-one (1i)**

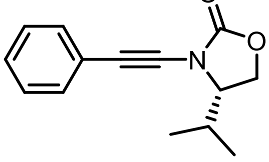

Prepared following the general procedure. All spectroscopic data were in good accordance with those reported in the literature.[6]

[5] D. L. Smith, W. R. F. Goundry, H. W. Lam, *Chem. Commun*. **2012**, *48*, 1505–1507.

3-(Oct-1-yn-1-yl)oxazolidin-2-one (1j)

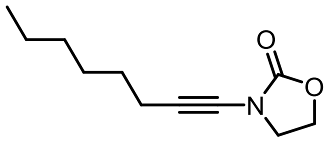

Prepared following the general procedure. All spectroscopic data were in good accordance with those reported in the literature.[6]

3-(Hex-1-yn-1-yl)oxazolidin-2-one (1k)

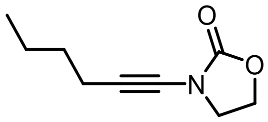

Prepared following the general procedure. All spectroscopic data were in good accordance with those reported in the literature.[3]

3-(3-Methylbut-1-yn-1-yl)oxazolidin-2-one (1l)

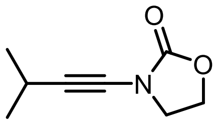

Prepared following the general procedure. All spectroscopic data were in good accordance with those reported in the literature.[2]

3-(3,3-Dimethylbut-1-yn-1-yl)oxazolidin-2-one (1m)

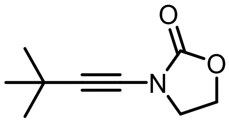

Prepared following the general procedure. All spectroscopic data were in good accordance with those reported in the literature.[7]

3-(3-Methylhex-1-yn-1-yl)oxazolidin-2-one (1n)

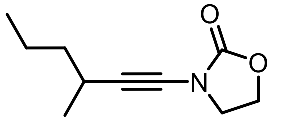

Prepared following the general procedure; (77%, colorless oil). **^{1}H-NMR (600 MHz, $CDCl_3$):** δ 4.04 (t, *J* = 8.4 Hz, 2H), 3.87 (t, *J* = 8.4 Hz, 2H), 2.59-2.53 (m, 1H), 1.55-1.36 (m, 4H), 1.17 (d, *J* = 7.2 Hz, 3H), 0.91 (t, *J* = 6.6 Hz, 3H); **^{13}C-NMR (150 MHz, $CDCl_3$):** δ 156.5, 75.4, 70.3, 62.7, 47.1, 39.1,

---

[6] V. Tona, S. A. Ruider, M. Berger, S. Shaaban, M. Padmanaban, L.-G. Xie, L. Gonzalez, N, Maulide, *Chem. Sci.* **2016**, *7*, 6032–6040.
[7] D. Kaldre, B. Maryasin, D. Kaiser, O. Gajsek, L. Gonzalez, N. Maulide, *Angew. Chem. Int. Ed*. **2017**, *56*, 2212–2215.

25.4, 21.1, 20.5, 13.9; **IR (neat) ν_{max}:** 2962, 2931, 2873, 1766, 1416, 1206, 1036, 751; **HRMS (ESI+):** exact mass calculated for $[M+Na]^+$ ($C_{10}H_{15}NO_2Na$) requires *m/z* 204.0995, found *m/z* 204.0994.

3-(Cyclopropylethynyl)oxazolidin-2-one (1o)

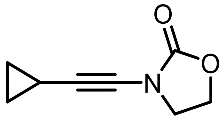

Prepared following the general procedure. All spectroscopic data were in good accordance with those reported in the literature.[2]

3-(Cyclohex-1-en-1-ylethynyl)oxazolidin-2-one (1p)

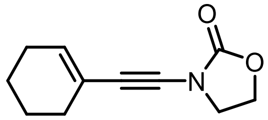

Prepared following the general procedure. All spectroscopic data were in good accordance with those reported in the literature.[6]

3-(5-Chloropent-1-yn-1-yl)oxazolidin-2-one (1q)

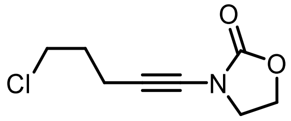

Prepared following the general procedure. All spectroscopic data were in good accordance with those reported in the literature.[6]

Methyl 7-(2-oxooxazolidin-3-yl)hept-6-ynoate (1r)

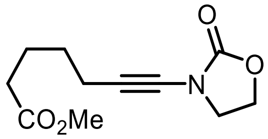

Prepared following the general procedure. All spectroscopic data were in good accordance with those reported in the literature.[2]

Methyl 11-(2-oxooxazolidin-3-yl)undec-10-ynoate (1s)

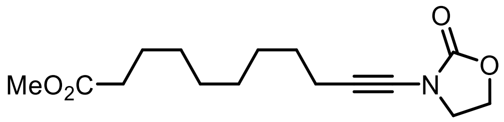

Prepared following the general procedure; (41% yield, yellow oil). **^{1}H-NMR (400 MHz, $CDCl_3$)** δ 4.51-4.32 (m, 2H), 4.01-3.84 (m, 2H), 3.69 (s, 3H), 2.35-2.29 (m, 4H), 1.73-1.61 (m, 2H), 1.58-1.48 (m, 2H), 1.47-1.23 (m, 8H); **^{13}C-NMR (100 MHz, $CDCl_3$)** δ 174.3, 156.6, 71.1, 70.1, 62.8, 51.4, 47.1, 34.1, 29.1, 29.1, 28.9, 28.7, 24.9, 18.4; **IR (neat) ν_{max}:** 2927, 2855, 2267, 1765, 1732,

1413, 1197, 1111; **HRMS (ESI+):** exact mass calculated for $[M+Na]^+$ ($C_{15}H_{23}NO_4Na$) requires *m/z* 304.1525, found *m/z* 304.1518.

2-(5-(2-oxooxazolidin-3-yl)pent-4-yn-1-yl)isoindoline-1,3-dione (1t)

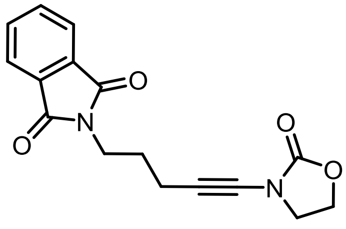
Prepared following the general procedure. All spectroscopic data were in good accordance with those reported in the literature. [8]

3-(5-phenylpent-1-yn-1-yl)oxazolidin-2-one (1u)

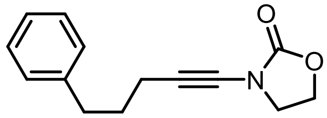
Prepared following the general procedure. All spectroscopic data were in good accordance with those reported in the literature.[2]

3-((3-methoxyphenyl)ethynyl)oxazolidin-2-one (1v)

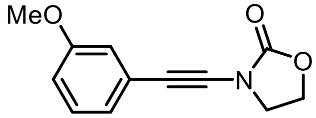

Prepared following the general procedure. All spectroscopic data were in good accordance with those reported in the literature.[9]

3-(cyclopentylethynyl)oxazolidin-2-one (1w)

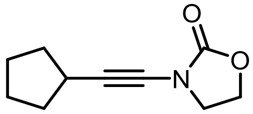
Prepared following the general procedure. All spectroscopic data were in good accordance with those reported in the literature.[2]

3-(3-cyclohexylprop-1-yn-1-yl)oxazolidin-2-one (1x)

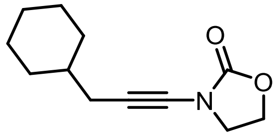
Prepared following the general procedure. All spectroscopic data were in good accordance with those reported in the literature.[2]

[8] P. W. Davies, A. Cremonesi, N. Martin, *Chem. Commun*. **2011**, *47*, 379–381.
[9] L.-G. Xie, S. Niyomchon, A. J. Mota, L. Gonzalez, M. Maulide, *Nat. Commun.* **2016**, *7*, 10914.

3-(3-((tert-butyldimethylsilyl)oxy)prop-1-yn-1-yl)oxazolidin-2-one (1y)

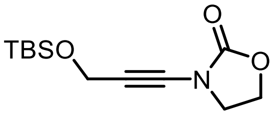

Prepared following the general procedure. All spectroscopic data were in good accordance with those reported in the literature.[10]

6-(2-oxooxazolidin-3-yl)hex-5-ynenitrile (1z)

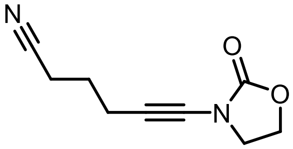

Prepared following the general procedure. All spectroscopic data were in good accordance with those reported in the literature.[2]

---

[10] B. Gourdet, M. E. Rudkin, C. A. Watts, H. W. Lam, *J. Org. Chem.* 2009, **74**, 7849.

4. Experimental Procedure and Characterization of Products

4.1. General Procedures TEMPO

Unless stated otherwise, all reactions were performed on a 0.2 mmol scale.

General procedure A

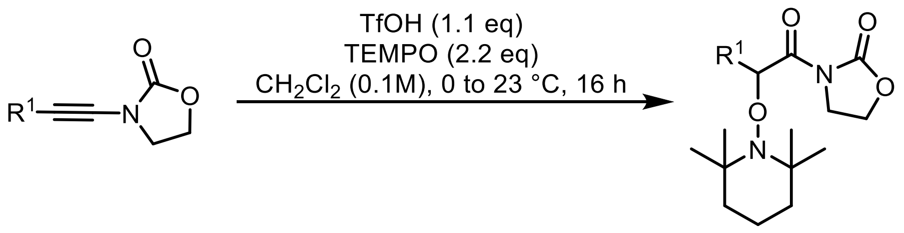

To a solution of the ynamide (1.0 equiv.) in dichloromethane (0.1M) at 0 °C was added trifluoromethanesulfonic acid (1.1 equiv.). After 15 minutes at 0 °C, TEMPO (2.2 equiv.) was added in one portion and the resulting reaction mixture was allowed to warm to ambient temperature over the course of 16 h. After this, H_2O was added, the resulting biphasic mixture was diluted with dichloromethane and the phases were separated. The aqueous phase was extracted with dichloromethane and the combined organic phases were dried over anhydrous sodium sulfate. The dried solution was filtered and the filtrate was concentrated under reduced pressure on a rotary evaporator. The crude residue was purified by flash column chromatography on silica gel (heptane/ethyl acetate) to afford the title compounds.

General procedure B

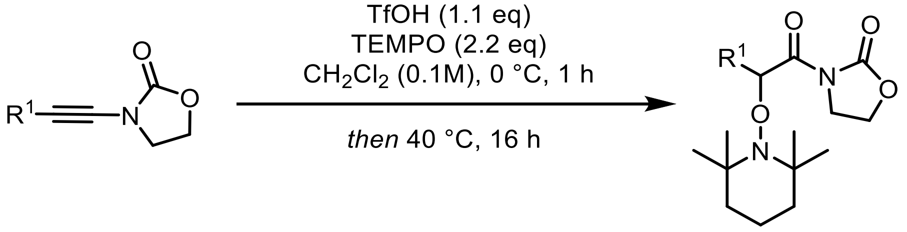

To a solution of the ynamide (1.0 equiv.) in dichloromethane (0.1M) at 0 °C was added trifluoromethanesulfonic acid (1.1 equiv.). After 15 minutes at 0 °C, TEMPO (2.2 equiv.) was added in one portion and the resulting reaction mixture was stirred for an additional 1 h at 0 °C. After this time, the reaction mixture was heated to 40 °C and stirring continued for 16 h. H_2O was added, the biphasic mixture was diluted with dichloromethane and the phases were separated. The aqueous phase was extracted with dichloromethane and the combined organic phases were dried over anhydrous sodium sulfate. The dried solution was filtered and the filtrate was concentrated under reduced pressure on a rotary evaporator. The crude residue was purified by flash column chromatography on silica gel (heptane/ethyl acetate) to afford the title compounds.

4.2. General Procedure TEMPO+

Synthesis of TEMPO+TfO-

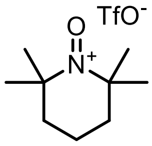

Prepared following the general procedure. All spectroscopic data were in good accordance with those reported in the literature.[11]

Synthesis of TEMPO+BF4-

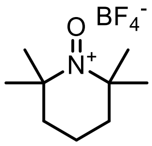

Prepared following the general procedure. All spectroscopic data were in good accordance with those reported in the literature.[12]

Unless stated otherwise, all reactions were performed on a 0.1 mmol scale.

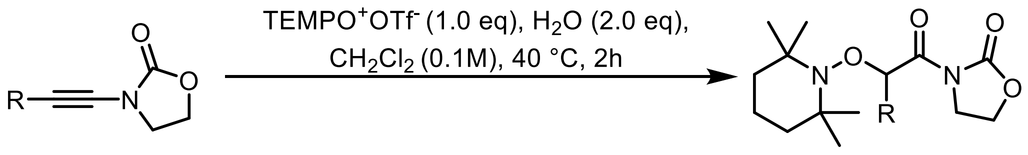

To a solution of the ynamide (1.0 equiv.) and Tempo+OTf- (1.00 equiv.) in dichloromethane (0.1M), water (2.0 equiv.) was added and the resulting mixture was stirred at 40 °C for 2h. After this time, the organic solution was washed sequentially with saturated aqueous sodium bicarbonate and saturated aqueous sodium chloride. The organic phase was dried over anhydrous magnesium sulfate, filtered and concentrated under reduced pressure. The resulting crude material was purified by flash column chromatography on silica gel (heptane/ethyl acetate) to afford the desired compound.

---

[11] M. Shibuya, M. Tomizawa, Y. Iwabuchi, *J. Org. Chem.* 2008, **73**, 4750.
[12] J. Wang, S. Yang, *Tetrahedron Letters*, 2016, **57**, 3444.

4.3. Characterization of Products

3-(2-Phenyl-2-((2,2,6,6-tetramethylpiperidin-1-yl)oxy)acetyl)oxazolidin-2-one (2a)

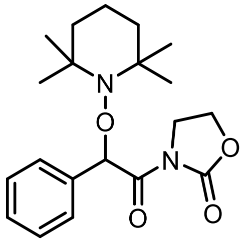

General Procedure A; (80%, dark yellow foam). **^{1}H-NMR (600 MHz, C_6D_6):** δ 7.99-7.96 (m, 2H), 7.17-7.12 (m, 3H), 7.03 (t, J = 5.0 Hz, 1H), 3.16-3.09 (m, 1H), 2.92-2.85 (m, 1H), 2.72-2.67 (m, 2H), 1.49-1.45 (m, 2H), 1.41 (br s, 7H), 1.35-1.33 (m, 1H), 1.26-1.24 (m, 1H), 1.12 (s, 4H), 0.92 (s, 3H); **^{13}C-NMR (151 MHz, C_6D_6):** δ 172.2, 153.3, 139.0 , 128.9 , 128.7 (2 carbons), 86.2 , 61.5 and 61.4 , 60.3 and 59.8 , 42.2 , 40.3 and 40.2 , 34.3 , 33.3 , 20.5 and 20.4 , 17.5 ; **IR (neat) ν_{max}:** 2932, 1778, 1707, 1387, 1219,1133, 759; **HRMS (ESI+):** exact mass calculated for $[M+H]^+$ ($C_{20}H_{29}N_2O_4$) requires *m/z* 361.2122, found *m/z* 361.2119.

3-(2-((2,2,6,6-Tetramethylpiperidin-1-yl)oxy)-2-(*p*-tolyl)acetyl)oxazolidin-2-one (2b)

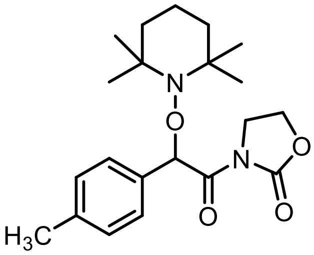

General Procedure A; (65%, white solid). **^{1}H-NMR (600 MHz, C_6D_6):** δ 7.91 (d, J = 7.8 Hz, 2H), 7.12 (s, 1H), 7.0 (d, J = 7.8 Hz, 2H), 3.14-3.10 (m, 1H), 2.90-2.87 (m, 1H), 2.77-2.69 (m, 2H), 1.99 (s, 3H), 1.51-1.34 (m, 10H), 1.27-1.26 (m, 1H), 1.15 (s, 4H), 0.97 (s, 3H); **^{13}C-NMR (151 MHz, C_6D_6):** δ 172.3, 153.2, 138.3, 136.0, 129.4, 128.8, 128.3, 86.1, 61.3, 60.2, 59.7, 42.2, 40.3 and 40.1, 34.4, 33.3, 21.0, 20.4, 20.3, 17.4; **IR (neat) ν_{max}:** 2927, 1776, 1704, 1383, 1361, 1042, 815; **HRMS (ESI+):** exact mass calculated for $[M+H]^+$ ($C_{21}H_{31}N_2O_4$) requires *m/z* 375.2278, found *m/z* 375.2279.

3-(2-(4-Chlorophenyl)-2-((2,2,6,6-tetramethylpiperidin-1-yl)oxy)acetyl)oxazolidin-2-one (2c)

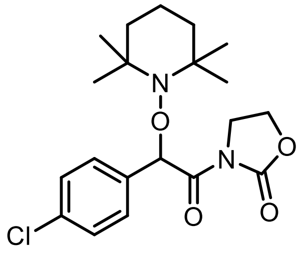

General Procedure A; (81%, colourless oil). **^{1}H-NMR (600 MHz, $CDCl_3$):** δ 7.51 (d, *J* = 8.5 Hz, 2H), 7.30 (d, *J* = 8.5 Hz, 2H), 6.60 (s, 1H), 4.45-4.40 (m, 1H), 4.36-4.30 (m, 1H), 4.08-4.02 (m, 1H), 3.90-3.85 (m, 1H), 1.57-1.48 (m, 1H), 1.48-1.43 (m, 2H), 1.41-1.36 (m, 2H), 1.32-1.27 (m, 1H), 1.20 (s, 3H), 1.08 (s, 3H), 10.01 (s, 3H), 0.67 (s, 3H); **^{13}C-NMR (151 MHz, $CDCl_3$):** δ 172.1, 153.1, 136.3, 134.1, 129.5, 128.5, 84.6, 62.2, 60.0 and 59.5, 42.5, 40.0 and 39.9, 33.9, 32.8, 20.0, 20.0, 17.0; **IR (neat) ν_{max}:** 2969, 2931, 1780, 1707, 1488, 1386, 1363, 1184, 1091, 1044, 820; **HRMS (ESI+):** exact mass calculated for $[M+H]^+$ ($C_{20}H_{28}N_2O_4{}^{35}Cl$) requires *m/z* 395.1732, found *m/z* 395.1735.

3-(2-(3,4-Dichlorophenyl)-2-((2,2,6,6-tetramethylpiperidin-1-yl)oxy)acetyl)oxazolidin-2-one (2d)

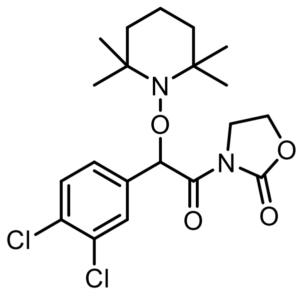

General Procedure A; (85%, colorless oil). **^{1}H-NMR (400 MHz, C_6D_6):** δ 8.15 (br s, 1H), 7.58 (d, *J* = 8.4 Hz, 1H), 7.06 (d, *J* = 8.4 Hz, 1H), 6.98 (s, 1H), 3.09-3.06 (m, 1H), 2.94 (br s, 1H), 2.81.2.75 (m, 2H), 1.48-1.38 (m, 2H), 1.34 (s, 3H), 1.30 (s, 3H), 1.26-1.23 (m, 2H), 1.10-1.08 (m, 1H), 1.05 (s, 3H), 0.89-0.84 (m, 1H), 0.82 (m, 3H); **^{13}C-NMR (100 MHz, C_6D_6):** δ 171.4, 153.3, 139.1, 133.2 and 133.1, 130.9 and 130.5, 84.9, 61.7, 60.4 and 59.9, 42.2, 40.3 and 40.1, 34.3, 33.2, 20.4 and 20.3, 17.4; **IR (neat) ν_{max}:** 2931, 1779, 1705, 1469, 1387, 1363, 1220, 957; **HRMS (ESI+):** exact mass calculated for $[M+H]^+$ ($C_{20}H_{27}Cl_2N_2O_4$) requires *m/z* 429.1342, found *m/z* 429.1350.

3-(2-(4-Fluoro-3-methylphenyl)-2-((2,2,6,6-tetramethylpiperidin-1-yl)oxy)acetyl)oxazolidin-2-one (2e)

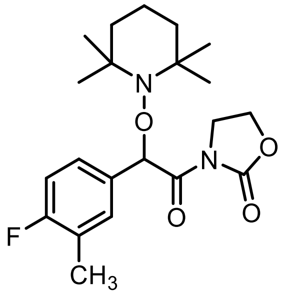

General Procedure A; (80%, reddish oil). **^{1}H-NMR (400 MHz, C_6D_6):** δ 7.80 (dd, *J* = 7.6, 1.6 Hz, 1H), 7.69 (ddd, *J* = 8.0, 5.2, 2.2 Hz, 1H), 7.05 (s, 1H), 6.83 (t, *J* = 9.0 Hz, 1H), 3.17-3.12 (m, 1H), 2.95-2.91 (m, 1H), 2.83-2.74 (m, 2H), 2.02 (d, *J* = 1.6 Hz, 3H), 1.52-1.45 (m, 3H), 1.41 (s, 4H), 1.37 (s, 3H), 1.28-1.26 (m, 1H), 1.14 (s, 4H), 0.94 (s, 3H); **^{13}C-NMR (100 MHz, C_6D_6):** δ 172.2, 161.9 (d, *J* = 205.5 Hz), 153.3, 134.6, 131.9, 125.4 and 125.3, 115.4 and 115.2, 85.5, 61.5, 60.4 and 59.8, 42.2, 40.4 and 40.2, 34.3, 33.3, 20.5 and 20.4, 17.5, 14.4; **IR (neat) ν_{max}:** 2928, 1776, 1703, 1499, 1361, 1198, 1111, 758, 713; **HRMS (ESI+):** exact mass calculated for $[M+H]^+$ ($C_{21}H_{30}FN_2O_4$) requires *m/z* 393.2184, found *m/z* 393.2187.

3-(2-((2,2,6,6-tetramethylpiperidin-1-yl)oxy)-2-(4-(trifluoromethyl)phenyl)acetyl)oxazolidin-2-one (2f)

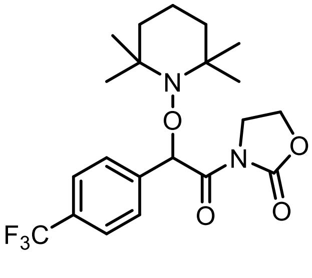

General Procedure A; (76%, colorless oil). **^{1}H-NMR (400 MHz, C_6D_6):** δ 7.89 (d, *J* = 8.2 Hz, 2H), 7.37 (d, *J* = 8.2 Hz, 2H), 7.09 (s, 1H), 3.12-3.06 (m, 1H), 2.96-2.89 (m, 1H), 2.83-2.70 (m, 2H), 1.44-1.34 (m, 10H), 1.24-1.21 (m, 1H), 1.08 (s, 4H), 0.80 (s, 3H); **^{13}C-NMR (100 MHz, C_6D_6):** δ 171.6, 153.3, 142.7, 130.8 (d, *J* = 33 Hz), 129.1, 125.7, 85.6, 61.6, 60.4 and 59.9, 42.2, 40.4 and 40.2, 34.2, 33.3, 20.4 and 20.3, 17.4; **IR (neat) ν_{max}:** 1777, 1706, 1363, 1111, 825, 712; **HRMS (ESI+):** exact mass calculated for $[M+H]^+$ ($C_{21}H_{28}F_3N_2O_4$) requires *m/z* 429.1996, found *m/z* 429.2002.

4-(2-Oxo-2-(2-oxooxazolidin-3-yl)-1-((2,2,6,6-tetramethylpiperidin-1-yl)oxy)ethyl)benzonitrile (2g)

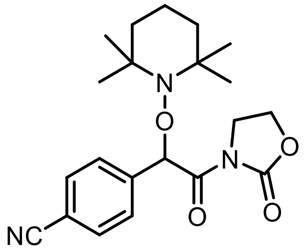

General Procedure A; (70%, white solid). **^{1}H-NMR (600 MHz, C_6D_6):** δ 7.70 (d, *J* = 7.8 Hz, 2H), 7.00 (s, 3H), 3.06-3.02 (m, 1H), 2.90-2.86 (m, 1H), 2.78-2.74 (m, 1H), 2.71-2.67 (m, 1H), 1.46-1.37 (m, 3H), 1.35 (s, 3H), 1.30 (s, 4H), 1.12-1.10 (m, 1H), 1.06 (s, 4H), 0.75 (s, 3H); **^{13}C-NMR (151 MHz, C_6D_6):** δ 171.2, 153.3, 143.1, 132.0, 129.1, 128.4, 118.5, 112.9, 85.4, 61.6, 60.4 and 59.8, 42.1, 40.2 and 40.1, 34.1, 33.2, 20.4 and 20.3, 17.4; **IR (neat) ν_{max}:** 2971, 2929, 2238, 1775, 1704, 1358, 1363, 1220, 824; **HRMS (ESI+):** exact mass calculated for $[M+H]^+$ ($C_{21}H_{28}N_3O_4$) requires *m/z* 386.2074, found *m/z* 386.2085.

3-(2-(naphthalen-2-yl)-2-((2,2,6,6-tetramethylpiperidin-1-yl)oxy)acetyl)oxazolidin-2-one (2h)

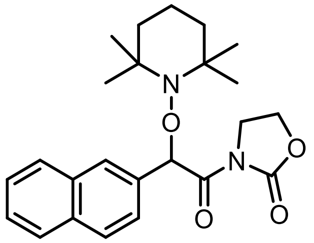

General Procedure B; (73%, brown foam). **^{1}H-NMR (400 MHz, C_6D_6):** δ 9.36 (d, *J* = 7.0 Hz, 1H), 8.29 (d, *J* = 7.0 Hz, 1H), 7.68 (br s, 1H), 7.57 (t, *J* = 8.2 Hz, 2H), 7.53-7.49 (m, 1H), 7.28-7.22 (m, 2H), 3.13-3.11 (m, 1H), 2.88-2.83 (m, 1H), 2.61-2.54 (m, 2H), 1.56-1.45 (s, 7H), 1.42-1.34 (m, 3H), 1.28-1.25 (m, 1H), 1.19-1.14 (m, 1H), 1.04 (s, 3H), 0.76 (s, 3H) ; **^{13}C-NMR (100 MHz, C_6D_6):** δ 171.92, 153.2, 135.0 and 134.9, 132.1, 129.7, 128.8, 126.6, 126.1, 125.6, 61.3, 60.5 and 59.8, 42.6, 40.3 and 40.2, 34.2, 33.4, 20.6 and 20.5, 17.5; **IR (neat) ν_{max}:** 2926, 1776, 1703, 1382, 1361, 1205, 806; **HRMS (ESI+):** exact mass calculated for $[M+H]^+$ ($C_{24}H_{31}N_2O_4$) requires *m/z* 411,2278, found *m/z* 411.2280.

(4l)-4-*Iso*propyl-3-(2-phenyl-2-((2,2,6,6-tetramethylpiperidin-1-yl)oxy)acetyl)oxazolidin-2-one (2i)

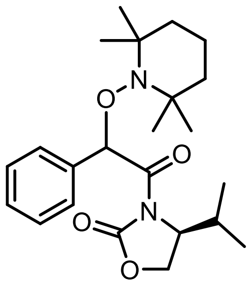

General Procedure A; (65%, d.r. 1.7:1; colorless oil). For simplicity, only the major diastereomer is reported. **^{1}H-NMR (600 MHz, C_6D_6):** δ 7.92 (dd, *J* = 8.4, 1.2 Hz, 2H), 7.25 (s, 1H), 7.12 (t, *J* = 7.2 Hz, 2H), 4.03 (dt, *J* = 7.2, 3.6, 1.2 Hz, 1H), 3.24-3.22 (m, 2H), 3.17 (t, *J* = 9.0 Hz, 1H), 1.82-1.77 (m, 1H), 1.45 (d, *J* = 8.4 Hz, 4H), 1.41 (br s, 4H), 1.37-1.31 (m, 2H), 1.14 (d, *J* = 9.0 Hz, 4H) 0.93 (d, *J* = 7.8 Hz, 4H), 0.17 (d, *J* = 6.6 Hz, 3H), 0.06 (d, *J* = 6.6 Hz, 3H); **^{13}C-NMR (151 MHz, C_6D_6):** δ 172.4, 153.9, 139.2, 128.9, 128.8, 128.7, 128.6, 86.7, 63.00, 60.4 and 59.8, 57.7, 40.3 and 40.2, 30.1, 28.0, 20.5 and 20.4, 17.6 and 17.5, 17.2, 14.0; **IR (neat) ν_{max}:** 2965, 2931, 1778, 1708, 1366, 1203, 753; **HRMS (ESI+):** exact mass calculated for $[M+H]^+$ ($C_{23}H_{35}N_2O_4$) requires *m/z* 403.2590, found *m/z* 403.2591.

3-(2-((2,2,6,6-Tetramethylpiperidin-1-yl)oxy)octanoyl)oxazolidin-2-one (2j)

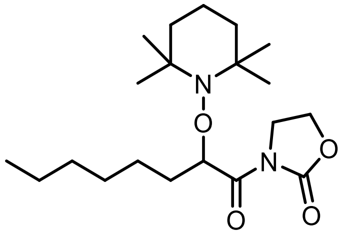

General Procedure B; (76%, yellow oil). **^{1}H-NMR (600 MHz, $CDCl_3$):** δ 5.70 (dd, *J* = 8.7, 3.9 Hz, 1H), 4.34-4.04 (m, 2H), 4.04-4.01 (m, 2H), 2.00-1.94 (m, 1H), 1.81-1.76 (m, 1H), 1.55-1.50 (m, 1H), 1.43 (br m, 4H), 1.27-1.22 (m, 12H), 1.12 (s, 3H), 1.10 (s, 3H), 0.96 (br s, 3H), 0.86 (t, *J* = 6.9 Hz, 3H); **^{13}C-NMR (151 MHz, $CDCl_3$):** δ 174.8, 153.1, 81.7, 62.2, 60.1 and 59.5, 42.4, 40.4 and 40.3, 33.8 and 33.2, 32.3 and 31.7, 29.6, 23.8 and 22.7, 20.2 and 20.0, 17.2, 14.2; **IR (neat) ν_{max}:** 2928, 2870, 1779, 1707, 1467, 991; **HRMS (ESI+):** exact mass calculated for $[M+H]^+$ ($C_{20}H_{37}N_2O_4$) requires *m/z* 369.2748, found *m/z* 369.2742.

3-(2-((2,2,6,6-Tetramethylpiperidin-1-yl)oxy)hexanoyl)oxazolidin-2-one (2k)

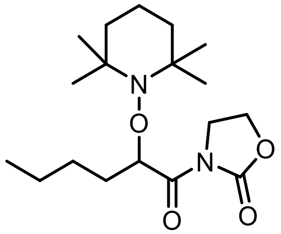

General Procedure B; (3 mmol scale; 66%, yellow oil). **^{1}H-NMR (600 MHz, C_6D_6):** δ 6.19 (dd, *J* = 4.7, 3.9 Hz, 1H), 3.27-3.22 (m, 1H), 3.13-3.00 (m, 3H), 2.32-2.24 (m, 1H), 2.06-1.98 (m, 1H), 1.53-1.39 (m, 8H), 1.33-1.26 (m, 7H), 1.25 (s, 3H), 1.23 (s, 3H), 0.90-0.86 (m, 1H), 0.85 (t, *J* = 7.3Hz, 3H); **^{13}C-NMR (151 MHz, C_6D_6):** δ 174.8, 153.7, 82.4, 62.1, 43.0, 41.4 and 41.1, 34.7 and 34.3, 33.2, 27.2, 24.0, 21.0, 18.1, 14.9; **IR (neat) ν_{max}:** 2930, 1780, 1707, 1647, 1383, 1361, 1261, 1218, 1042; **HRMS (ESI+):** exact mass calculated for $[M+H]^+$ ($C_{18}H_{33}N_2O_4$) requires *m/z* 341.2435, found *m/z* 341.2432.

3-(3-Methyl-2-((2,2,6,6-tetramethylpiperidin-1-yl)oxy)butanoyl)oxazolidin-2-one (2l)

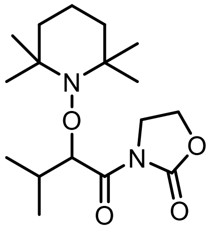

General Procedure B; (64%, colorless oil). **^{1}H-NMR (400 MHz, C_6D_6):** δ 6.18 (d, *J* = 6.8 Hz, 1H), 3.31-3.26 (m, 1H), 3.16-3.05 (m, 3H), 2.54-2.49 (m, 1H), 1.44 (bs, 6H), 1.23 (br s, 10H), 1.16 (dd, *J* = 6.8, 1.2 Hz, 4H), 1.09 (d, *J* = 6.8 Hz, 4H); **^{13}C-NMR (100 MHz, C_6D_6):** δ 173.4, 153.2, 83.3, 61.6, 60.9 and 59.7, 42.2, 40.9, 34.2, 31.9, 20.5, 18.8, 17.5; **IR (neat) ν_{max}:** 2966, 2929, 1776, 1707, 1381, 1209, 1042, 710; **HRMS (ESI+):** exact mass calculated for $[M+H]^+$ ($C_{17}H_{31}N_2O_4$) requires *m/z* 327.2278, found *m/z* 327.2277.

3-(3,3-Dimethyl-2-((2,2,6,6-tetramethylpiperidin-1-yl)oxy)butanoyl)oxazolidin-2-one (2m)

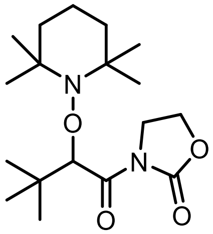

General Procedure A (81%, yellowish oil). **^{1}H-NMR (600 MHz, C_6D_6):** δ 6.28 (s, *J* = 6.8 Hz, 1H), 3.27-3.23 (m, 1H), 3.13-3.09 (m, 1H), 2.99-2.95 (m, 1H), 2.89 (q, *J* = 8.7 Hz, 1H), 1.61 (br s, 2H), 1.47 (br m, 3H), 1.37 (br s, 4H), 1.26 (br s, 15H), 1.11 (s, 1H), 1.05 (br m, 1H); **^{13}C-NMR (151 MHz, $CDCl_3$):** δ 173.1, 153.2, 81.9, 61.6, 45.8, 42.8, 41.2 and 40.8, 36.2, 34.5 and 34.3, 30.3, 29.5, 26.4 (3C), 20.2, 17.0; **IR (neat) ν_{max}:** 2951, 2931, 1777, 1711, 1382, 1260, 1184, 761; **HRMS (ESI+):** exact mass calculated for $[M+H]^+$ ($C_{18}H_{33}N_2O_4$) requires *m/z* 341.2425, found *m/z* 341.2435.

3-(3-Methyl-2-((2,2,6,6-tetramethylpiperidin-1-yl)oxy)hexanoyl)oxazolidin-2-one (2n)

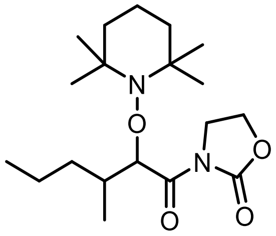

General Procedure B; (64%, d.r. 2:1; yellowish oil). For simplicity, only the major diastereomer is reported. **^{1}H-NMR (600 MHz, C_6D_6):** δ 6.24 (dd, *J* = 9.6, 7.2 Hz, 1H), 3.32-3.26 (m, 1H), 3.16-3.11 (m, 1H), 3.09-3.02 (m, 2H), 2.45-2.38 (m, 1H), 1.91-1.84 (m, 1H), 1.50-1.38 (m, 5H), 1.37-1.29 (m, 4H), 1.24 (br s, 11H), 1.11 (d, *J* = 7.2 Hz, 3H), 1.03 (t, *J* = 7.2 Hz, 1H), 0.92 (t, *J* = 7.2 Hz, 3H); **^{13}C-NMR (151 MHz, C_6D_6):** δ 173.1, 153.3 153.2, 83.1, 82.5, 81.5, 61.2, 45.9, 42.6 and 42.5, 40.9, 40.8 and 40.7, 37.0, 36.7, 34.9, 34.2, 34.1, 23.2, 21.0, 20.5, 17.5, 15.7, 14.5, 12.7 ; **IR (neat) ν_{max}:** 2928, 1776, 1703, 1379, 1200, 1042, 711; **HRMS (ESI+):** exact mass calculated for $[M+H]^+$ ($C_{19}H_{35}N_2O_4$) requires *m/z* 355.2591, found *m/z* 355.2592.

3-(2-((*tert*-Butyldiphenylsilyl)oxy)-2-cyclopropylacetyl)oxazolidin-2-one (2o)

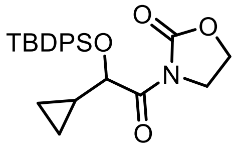

1st step: General Procedure B.

2nd step: Reductive cleavage of the above crude was performed under the conditions reported in the literature.[13]

3rd step: TBDPSCl (2.0 equiv) was added in one portion to a stirred solution of the crude alcohol (prepared see step 2; 1.0 equiv) and imidazole (6.0 equiv) in dry DMF (0.5M). After stirring for 24h, saturated aqueous sodium bicarbonate was added and the mixture diluted with dichloromethane. The phases were separated and the aqueous phase was further extracted with dichloromethane. The combined organic extracts were dried over anhydrous sodium sulfate, filtered and the solvent evaporated. Preparative TLC (7:3-heptane/ethyl acetate) afforded compound **2o** (35%, colorless oil). **^{1}H-NMR (600 MHz, $CDCl_3$):** δ 7.67 (d, *J* = 6.6 Hz, 2H), 7.61 (d, *J* = 6.6 Hz, 2H), 7.43-7.34 (m, 6H), 5.26 (d, *J* = 6.0 Hz, 1H), 4.24-4.19 (m, 1H), 4.08-4.03 (m, 1H), 3.72-3.68 (m, 1H), 3.39-3.35 (m, 1H), 1.29-1.23 (m, 1H), 1.11 (s, 9H), 0.71-0.67 (m, 1H), 0.58-0.54 (m, 1H), 0.50-0.46 (m, 1H), 0.44-0.40 (m, 1H); **^{13}C-NMR (151 MHz, $CDCl_3$):** δ 173.5, 152.6, 136.2, 135.9, 135.5 and 133.4, 129.6, 127.5 and 127.4, 70.8, 62.0, 42.2, 26.9, 14.9, 1.88, 1.10; **IR (neat) ν_{max}:** 2931, 2858, 1775, 1710, 1387, 1109, 704; **HRMS (ESI+):** exact mass calculated for $[M+H]^+$ ($C_{24}H_{30}NO_4Si$) requires *m/z* 424.1939, found *m/z* 424.1934.

3-(2-(Cyclohex-1-en-1-yl)-2-((2,2,6,6-tetramethylpiperidin-1-yl)oxy)acetyl)oxazolidin-2-one (2p)

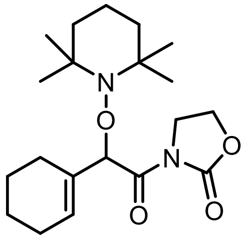

General Procedure B; (88%, colorless oil). **^{1}H-NMR (600 MHz, C_6D_6):** δ 7.62, (s, 1H), 4.37-4.35 (m, 1H), 3.48-3.46 (m, 1H), 3.01-3.00 (m, 5H), 2.76-2.72 (m, 1H), 2.14-2.09 (m, 1H), 1.83-1.78 (m, 1H), 1.72-1.66 (m, 1H), 1.58 (t, *J* = 5.4 Hz, 2H), 1.45 (br s, 2H), 1.35 (br s, 6H), 1.30 (s, 3H), 1.28 (s, 4H), 1.24 (s, 3H), ; **^{13}C-NMR (151 MHz,C_6D_6):** δ 165.4, 163.7, 153.0, 113.1, 86.3, 61.0, 60.5 and 59.8, 42.3, 40.6, 35.9 and 34.7,

[13] A. de la Torre, D. Kaiser, N. Maulide, *J. Am. Chem. Soc.* **2017**, *139*, 6578–6581.

34.4, 29.6, 28.8, 24.7, 20.8 and 20.7, 17.6; **IR (neat) ν_{max}:** 2928, 1774, 1676, 1630, 1383, 1191, 1041, 760; **HRMS (ESI+):** exact mass calculated for $[M+H]^+$ ($C_{20}H_{33}N_2O_4$) requires *m/z* 365.2435, found *m/z* 365.2434.

3-(5-Chloro-2-((2,2,6,6-tetramethylpiperidin-1-yl)oxy)pentanoyl)oxazolidin-2-one (2q)

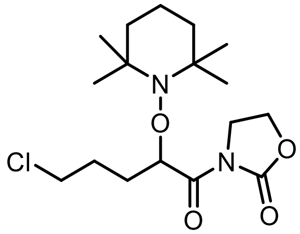

General Procedure B; (76%, yellow oil). **^{1}H-NMR (400 MHz, C_6D_6):** δ 6.11 (dd, *J* = 6.8, 3.6 Hz, 1H), 3.26-3.17 (m, 3H), 3.08-3.05 (m, 3H), 2.34-2.23 (m, 1H), 2.03-1.95 (m, 1H), 1.85-1.81 (m, 2H), 1.41 (br s, 3H), 1.32-1.26 (m, 9H), 1.19 (d, *J* = 9.2 Hz, 6H); **^{13}C-NMR (100 MHz, C_6D_6):** δ 173.5, 153.1, 81.5, 61.6, 60.4 and 59.9, 45.0, 42.2, 40.7 and 40.5, 34.1 and 33.6, 29.8, 27.6, 20.4, 17.4; **IR (neat) ν_{max}:** 2930, 1782, 1707, 1349, 1190, 1135, 1059; **HRMS (ESI+):** exact mass calculated for $[M+H]^+$ ($C_{17}H_{30}$ClN2O4) requires *m/z* 361.1889, found *m/z* 361.1894.

Methyl 6-oxo-6-(2-oxooxazolidin-3-yl)-5-((2,2,6,6-tetramethylpiperidin-1-yl)oxy)hexanoate (2r)

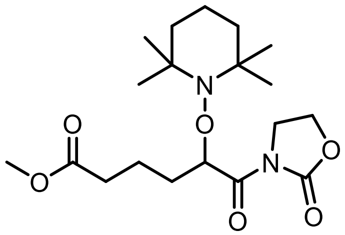

General Procedure B; (49%, colorless oil). **^{1}H-NMR (600 MHz, C_6D_6):** δ 6.17-6.15 (m, 1H), 3.28 (s, 3H), 3.25-3.20 (m, 1H), 3.13-3.03 (m, 3H), 2.28-2.22 (m, 1H), 2.16 (t, *J* = 7.2 Hz, 2H), 1.99-1.93 (m, 1H), 1.85-1.80 (m, 2H), 1.43 (br s, 3H), 1.35 (br s, 4H), 1.28 (br s, 5H), 1.24 (br s, 4H), 1.20 (br s, 4H); **^{13}C-NMR (100 MHz, C_6D_6):** δ 173.7 and 173.2, 153.2, 81.9, 61.6, 60.3, 59.9, 51.0, 42.2, 40.7 and 40.4, 34.1, 33.8 and 33.7, 31.8, 20.4, 19.8, 17.5; **IR (neat) ν_{max}:** 2928, 1776, 1734, 1705, 1383, 1199, 1133, 956, 712; **HRMS (ESI+):** exact mass calculated for $[M+H]^+$ ($C_{19}H_{35}N_2O_4$) requires *m/z* 355.2591, found *m/z* 355.2592.

Methyl-11-oxo-11-(2-oxooxazolidin-3-yl)-10-((2,2,6,6-tetramethylpiperidin-1-yl)oxy)undecanoate (2s)

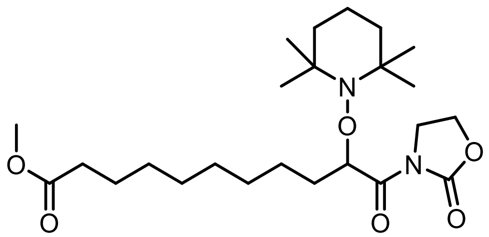

General Procedure B; (66%, dark yellow oil). **^{1}H-NMR (600 MHz, C_6D_6):** δ 6.20 (dd, *J* = 9.4, 3.8 Hz, 1H), 3.35 (s, 3H), 3.29-3.24 (m, 1H), 3.17-3.12 (m, 1H), 3.08-3.06 (m, 2H), 2.33-2.26 (m, 1H), 2.08 (d, *J* = 7.4 Hz, 2H), 2.06-2.01 (m, 1H), 1.55-1.48 (m, 5H), 1.46-1.43 (m 5H), 1.30 (s, 6H), 1.26 (br s, 4H), 1.24 (br s, 3H), 1.20-1.16 (m, 3H), 1.12-1.11 (br m, 4H); **^{13}C-NMR (151 MHz, C_6D_6):** δ 174.7 and 174.0, 153.7, 82.4, 62.0, 61.0 and 60.3, 51.5, 42.9, 41.3 and 41.0, 34.7 and 34.6, 34.2 and 33.4, 30.8 and 30.2, 30.1 and 29.9, 25.8, 25.0, 21.0, 18.0; **IR (neat) ν_{max}:** 2926, 2855, 1778, 1736, 1707, 1382, 1199, 761; **HRMS (ESI+):** exact mass calculated for $[M+H]^+$ ($C_{24}H_{43}N_2O_6$) requires *m/z* 455.3116, found *m/z* 455.3115.

2-(5-Oxo-5-(2-oxooxazolidin-3-yl)-4-((2,2,6,6-tetramethylpiperidin-1-yl)oxy)pentyl)isoindoline-1,3-dione (2t)

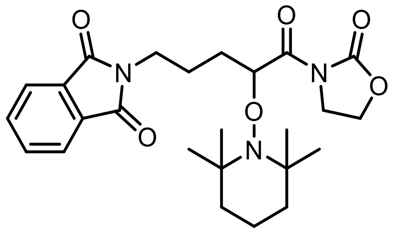

General Procedure B; (82%, yellow sticky oil). **^{1}H-NMR (600 MHz, C_6D_6):** δ 7.39-7.38 (m, 2H), 6.84-6.82 (m, 2H), 6.21-6.20 (m, 1H), 3.57 (t, *J* = 7.2 Hz, 2H), 3.28-3.24 (m, 4H), 3.10-3.08 (m, 1H), 2.31-2.26 (m, 1H), 2.00-1.95 (m, 2H), 1.88-1.85 (m, 1H), 1.43-1.41 (m, 2H), 1.34 (s, 3H), 1.26 (s, 4H), 1.24 (s, 3H), 1.20 (s, 3H), 1.12 (t, *J* = 6.9 Hz, 1H), 1.09-1.07 (m, 1H); **^{13}C-NMR (151 MHz, C_6D_6):** δ 173.7, 168.0, 153.3, 133.3, 132.6, 128.4, 122.9, 81.3, 65.9, 61.6, 60.3 and 59.9, 42.3, 40.6 and 40.4,37.9, 34.0 and 33.6, 29.6, 23.3, 20.4, 17.4, 15.6; **IR (neat) ν_{max}:** 2932, 1774, 1708, 1395, 1043, 720; **HRMS (ESI+):** exact mass calculated for $[M+H]^+$ ($C_{25}H_{34}N_3O_6$) requires *m/z* 472.2446, found *m/z* 472.2442.

3-(5-Phenyl-2-((2,2,6,6-tetramethylpiperidin-1-yl)oxy)pentanoyl)oxazolidin-2-one (2u)

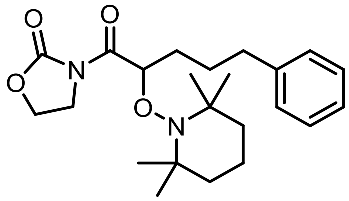

General procedure TEMPO$^+$; (65%, colourless oil). **^{1}H NMR (400 MHz, CDCl$_3$):** δ 7.27-7.24 (m, 2H), 7.18-7.15 (m, 3H), 5.73 (dd, *J* = 8.6, 3.8 Hz, 1H), 4.40 – 4.35 (m, 2H), 4.04 – 3.92 (m, 2H), 2.61 (td, *J* = 7.8, 3.3 Hz, 2H), 2.10 – 2.01 (m, 1H), 1.87 – 1.78 (m, 1H), 1.67 – 1.57 (m, 3H), 1.41 (s, br, 4H), 1.26 (br, 2H), 1.17 (s, br, 3H), 1.11 (s, br, 5H), 0.96 (s, br, 3H); **^{13}C NMR (101 MHz, CDCl$_3$):** δ 174.51, 153.11, 142.16, 128.63, 128.40, 125.90, 81.65, 62.22, 42.58, 40.50, 36.06, 33.80, 33.24, 31.81, 29.84, 25.65, 20.24, 17.25; ; **IR (neat) ν_{max}:** 2923, 2853, 1781, 1465; **HRMS (ESI+):** exact mass calculated for $[M+Na]^+$ ($C_{23}H_{34}N_2O_4Na^+$) requires *m/z* 425.2411, found *m/z* 425.2510.

3-(2-(3-Methoxyphenyl)-2-((2,2,6,6-tetramethylpiperidin-1-yl)oxy)acetyl)oxazolidin-2-one (2v)

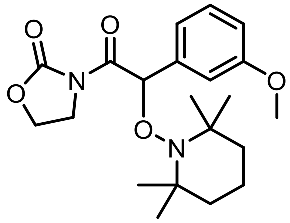

General procedure TEMPO$^+$; (59%, pale-yellow solid). **^{1}H NMR (600 MHz, CDCl$_3$):** δ 7.22 (t, *J* = 7.9 Hz, 1H), 7.16-7.16 (m, 2H), 6.84 – 6.82 (m, 1H), 6.62 (s, 1H), 4.41 (td, *J* = 9.2, 5.9 Hz, 1H), 4.31 (dd, *J* = 16.8, 9.0 Hz, 1H), 4.08-4.03 (m, 1H), 3.90-3.86 (m, 1H), 3.81 (s, 3H), 1.45 (d, *J* = 8.8 Hz, 2H), 1.39 (d, *J* = 6.2 Hz, 2H), 1.29 – 1.23 (m, 2H), 1.21 (s, 3H), 1.09 (s, 3H), 1.03 (s, 3H), 0.69 (s, 3H). **^{13}C NMR (151 MHz, CDCl$_3$):** δ 172.42, 159.50, 153.20, 139.45, 129.34, 120.55, 114.34, 112.94, 85.26, 62.21, 60.00, 59.55, 55.40, 42.61, 40.06, 40.00, 33.80, 32.89, 20.19, 20.14, 17.17; **IR (neat) ν_{max}:** 3362, 2923, 2852, 1742, 1660, 1466, 1259; **HRMS (ESI+):** exact mass calculated for $[M+H]^+$ ($C_{21}H_{31}N_2O_5^+$) requires *m/z* 391.2227, found *m/z* 391.2227.

3-(2-Cyclopentyl-2-((2,2,6,6-tetramethylpiperidin-1-yl)oxy)acetyl)oxazolidin-2-one (2w)

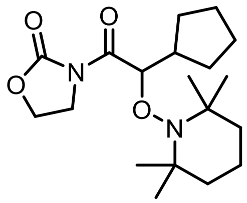

General procedure TEMPO$^+$; (62%, colourless oil). **^{1}H NMR (600 MHz, CDCl$_3$):** δ 5.80 (d, *J* = 8.6 Hz, 1H), 4.44 – 4.37 (m, 2H), 4.05 – 3.98 (m, 2H), 2.43 – 2.36 (m, 1H), 1.94 – 1.89 (m, 1H), 1.66 – 1.33 (m, 13H), 1.29 (s, 3H), 1.16 (s, 3H), 1.08 (s, 3H), 0.94 (s, 3H). **^{13}C NMR (151 MHz, CDCl$_3$):** δ 174.51, 153.23, 81.75, 62.01, 60.92, 59.32, 42.96, 42.77, 40.69, 33.93, 33.79, 29.67, 27.38, 25.25, 24.38, 20.16, 20.13, 17.16; **IR (neat) ν_{max}:** 2927, 1781, 1706, 1382, 1275, 1100, 1042; **HRMS (ESI+):** exact mass calculated for [M+H]$^+$ ($C_{19}H_{33}N_2O_4^+$) requires *m/z* 353.2435, found *m/z* 353.2434.

3-(3-Cyclohexyl-2-((2,2,6,6-tetramethylpiperidin-1-yl)oxy)propanoyl)oxazolidin-2-one (2x)

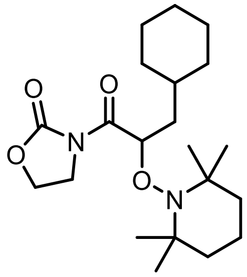

General procedure TEMPO$^+$; (62%, colourless oil). **^{1}H NMR (600 MHz, CDCl$_3$):** δ 5.79 (d, *J* = 7.1 Hz, 1H), 4.45 – 4.38 (m, 2H), 4.06 – 3.99 (m, 2H), 1.91 – 1.84 (m, 2H), 1.72 – 1.59 (m, 6H), 1.54 – 1.53 (m, 5H), 1.27 (m, 1H), 1.23 (s, 3H), 1.19 – 1.11 (m, 1H), 1.10 (s, 3H), 1.06 (s, 3H), 0.99 – 0.95 (m, 1H), 0.94 (s, 3H), 0.91 – 0.85 (m, 1H); **^{13}C NMR (151 MHz, CDCl$_3$):** δ 175.22, 153.02, 79.21, 62.07, 60.52, 59.15, 42.74, 40.44, 40.20, 40.07, 34.45, 33.95, 33.58, 33.35, 33.21, 26.45, 26.38, 26.27, 20.18, 20.08, 17.17; **IR (neat) ν_{max}:** 2924, 1782, 1706, 1382, 1206, 1106, 1042; **HRMS (ESI+):** exact mass calculated for [M+H]$^+$ ($C_{21}H_{37}N_2O_4^+$) requires *m/z* 381.2748, found *m/z* 381.2748.

3-(3-((*tert*-Butyldimethylsilyl)oxy)-2-((2,2,6,6-tetramethylpiperidin-1-yl)oxy)propanoyl)oxazolidin-2-one (2y)

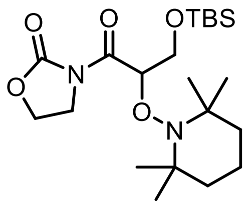

General procedure TEMPO$^+$; (41%, pale yellow oil). **^{1}H NMR (600 MHz, CDCl$_3$):** δ 5.97 (t, *J* = 7.5 Hz, 1H), 4.37 (dq, *J* = 36.9, 8.3 Hz, 2H), 4.11 (t, *J* = 7.8 Hz, 1H), 4.02 (t, *J* = 8.0 Hz, 2H), 3.75 (t, *J* = 8.9 Hz, 1H), 1.58–1.25 (m, 6H), 1.22 (s, 3H), 1.10 (s, 3H), 1.10 (s, 3H), 0.99 (s, 3H), 0.83 (s, 9H), 0.02 (s, 6H); **^{13}C NMR (151 MHz, CDCl$_3$):** δ 174.41, 153.08, 81.89, 63.11, 62.02, 60.29, 59.56, 42.56, 40.37, 40.29, 33.41, 33.16, 25.82, 20.22, 18.20, 17.24, -5.46, -5.68; **IR (neat) ν_{max}:** 2926, 2854, 1786, 1710, 1260, 1108, 838;**HRMS (ESI+):** exact mass calculated for [M+H]$^+$ ($C_{21}H_{41}N_2O_5Si^+$) requires *m/z* 429.2779, found *m/z* 429.2777.

6-Oxo-6-(2-oxooxazolidin-3-yl)-5-((2,2,6,6-tetramethylpiperidin-1-yl)oxy)hexanenitrile (2z)

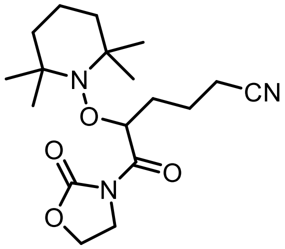

^{1}H NMR (600 MHz, CDCl$_3$): δ 5.75 (d, *J* = 5.3 Hz, 1H), 4.48 – 4.41 (m, 2H), 4.03 (t, *J* = 8.0 Hz, 2H), 2.48 – 2.32 (m, 2H), 2.24 – 2.18 (m, 1H), 1.90 - 1.86 (m, 1H), 1.83 – 1.65 (m, 2H), 1.52 (br, 1H), 1.48 – 1.35 (m, 4H), 1.29 – 1.24 (m, 1H), 1.21 (s, 3H), 1.12 (s, 3H), 1.10 (s, 3H), 0.96 (s, 3H); **^{13}C NMR (151 MHz, CDCl$_3$):** δ 173.65, 153.18, 119.64, 81.09, 62.44, 60.16, 59.78, 42.56, 40.45, 40.23, 33.94, 33.21, 30.67, 20.35, 20.19, 20.16, 17.37, 17.15; **IR (neat) ν_{max}:** 2933, 1777, 1705, 1387, 1266, 1115; **HRMS (ESI+):** exact mass calculated for [M+Na]$^+$ ($C_{18}H_{29}N_3O_4Na^+$) requires *m/z* 374.2050, found *m/z* 374.2046.

4.4. Products of Reductive (3) and Oxidative (4) Cleavage

Reductive and oxidative cleavage of **2k** were performed under the conditions reported in the literature.[9]

3-(2-Hydroxyhexanoyl)oxazolidin-2-one (3)

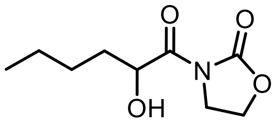
(77%, yellow sticky oil). **^{1}H-NMR (400 MHz, $CDCl_3$):** δ 5.01 (td, *J* = 7.7, 3.4 Hz, 1H), 4.45-4.44 (m, 2H), 4.14-3.98 (m, 2H), 3.24 (d, *J* = 8.4 Hz, 1H), 1.88-1.78 (m, 1H), 1.61-1.52 (m, 1H), 1.51-1.30 (m, 4H), 0.91 (t, *J* = 7.3 Hz, 3H); **^{13}C-NMR (100 MHz, C_6D_6):** δ 175.7, 153.3, 70.9, 63.0, 42.7, 34.5, 27.5, 22.5, 14.0; **IR (neat) ν_{max}:** 2958, 2930, 1778, 1739, 1699, 1389, 1120, 1080, 1040; **HRMS (ESI+):** exact mass calculated for $[M+Na]^+$ ($C_9H_{15}NO_4Na$) requires *m/z* 224.0893, found *m/z* 224.0895.

1-(2-Oxooxazolidin-3-yl)hexane-1,2-dione (4)

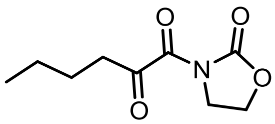
(80%, yellow sticky oil). **^{1}H-NMR (400 MHz, $CDCl_3$):** δ 4.60-4.54 (m, 2H), 4.05-3.99 (m, 2H), 2.71 (t, *J* = 7.4 Hz, 2H), 1.72-1.64 (m, 2H), 1.44-1.34 (m, 2H), 0.92 (t, *J* = 7.4 Hz, 3H); **^{13}C-NMR (100 MHz, C_6D_6):** δ 197.8, 167.4, 153.6, 64.2, 40.9, 39.2, 24.4, 22.1, 13.8; **IR (neat) ν_{max}:** 2960, 2933, 1783, 1722, 1692, 1393, 1365, 1229, 1140, 1123, 1032; **HRMS (ESI+):** exact mass calculated for $[M+Na]^+$ ($C_9H_{13}NO_4Na$) requires *m/z* 222.0737, found *m/z* 222.0736.

5. Mechanistic Experiments

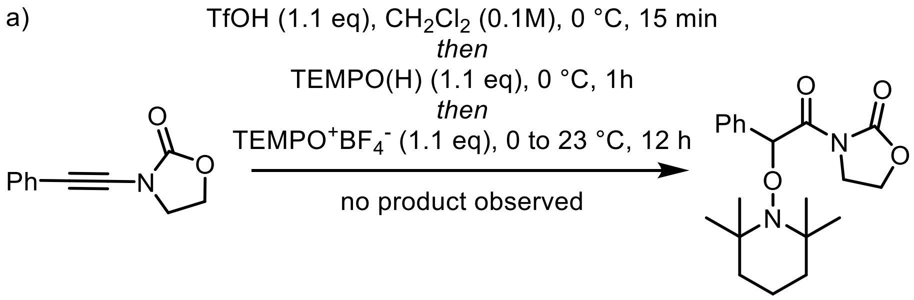

1a **2a**

To a solution of ynamide **1a** (1.0 equiv.) in dichloromethane (0.1 M) at 0 °C was added trifluoromethanesulfonic acid (1.1 equiv.). After 15 minutes at 0 °C, TEMPO^{-}Na^{+},[14] or TEMPOH[15] (1.1 equiv.) was added in one portion and the resulting reaction mixture was stirred at 0 °C for 1 h. After this time, TEMPO^{+}BF$_4^{-}$ (1.1 equiv.)[16] was added and the resulting mixture was allowed to warm to 23 °C over the course of 12 h. After this time, H_2O was added, the resulting biphasic mixture was diluted with dichloromethane and the phases were separated. The aqueous phase was extracted with dichloromethane and the combined organic phases were dried over anhydrous sodium sulfate. The dried solution was filtered and the filtrate was concentrated under reduced pressure on a rotary evaporator. NMR-analysis of the resulting crude mixture did not show any product formation.

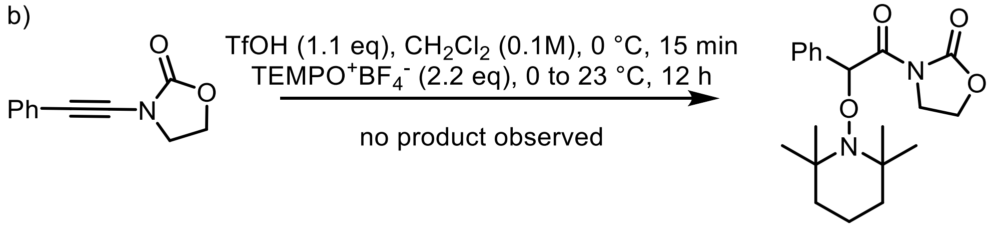

1a **2a**

To a solution of ynamide **1a** (1.0 equiv.) in dichloromethane (0.1 M) at 0 °C was added trifluoromethanesulfonic acid (1.1 equiv.). After 15 minutes at 0 °C, TEMPO^{+}BF$_4^{-}$,[16] (2.2 equiv.) was added and the resulting mixture was allowed to warm to 23 °C over the course of 12 h. After this time, H_2O was added, the resulting biphasic mixture was diluted with dichloromethane and the phases were separated. The aqueous phase was extracted with dichloromethane and the combined organic phases

[14] Prepared following the procedure reported in: L. Balloch, A. M. Drummond, P. García-Álvarez, D. V. Graham, A. R. Kennedy, J. Klett, R. E. Mulvey, C. T. O'Hara, P. J. A. Rodger, I. D. Rushworth, *Inorg. Chem*. **2009**, *48*, 6934–6944.
[15] Prepared following the procedure reported in: S. R. McCabe, P. Wipf, *Angew. Chem. Int. Ed*. **2017**, *56*, 324–327.
[16] Prepared following the procedure reported in: M. Shiyuba, M. Tomizawa, Y. Iwabuchi, *J. Org. Chem*. **2008**, *73*, 4750–4752.

were dried over anhydrous sodium sulfate. The dried solution was filtered and the filtrate was concentrated under reduced pressure on a rotary evaporator. NMR-analysis of the resulting crude mixture did not show any product formation.

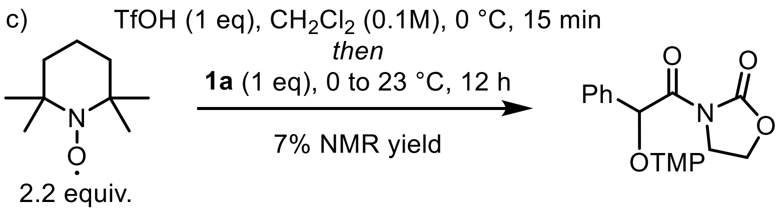

2a

To a solution of TEMPO (2.2 equiv.) in dichloromethane (0.1 M) at 0 °C was added trifluoromethanesulfonic acid (1.1 equiv.). After 15 minutes at 0 °C, ynamide **1a** (1.0 equiv.) was added and the resulting mixture was allowed to warm to 23 °C over the course of 12 h. After this time, H_2O was added, the resulting biphasic mixture was diluted with dichloromethane and the phases were separated. The aqueous phase was extracted with dichloromethane and the combined organic phases were dried over anhydrous sodium sulfate. The dried solution was filtered and the filtrate was concentrated under reduced pressure on a rotary evaporator. NMR-analysis of the resulting crude mixture, using 1,3,5-trimethoxybenzene as the internal standard, indicated a yield of 7% of the desired product **2a**.

Isotopic Labelling

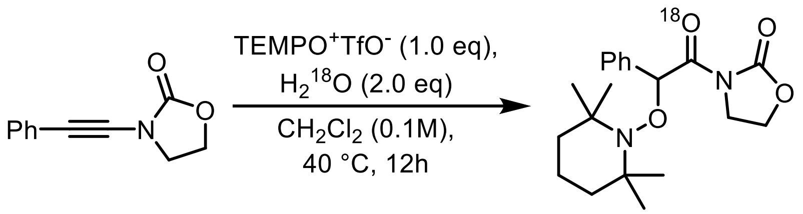

To a solution of the ynamide (1.0 equiv.) and Tempo$^+$OTf$^-$ (1.00 equiv.) in dichloromethane (0.1 M), water (2.0 equiv., $H_2{}^{18}O$ 83% isotopical label) was added and the resulting mixture was stirred at 40 °C for 12h. After this time, the organic solution was filtered and the filtrate was dried over anhydrous magnesium sulfate, filtered again and concentrated under reduced pressure. The resulting crude material was purified by flash column chromatography on silica gel (heptane/ethyl acetate) to afford the desired compound in 58% yield. ^{13}C NMR: in correspondence to the alpha carbonyl an isotopic peak is present.
HRMS-analysis: exact mass calculated for [M+H]$^+$ ($C_{20}H_{29}O_3N_2{}^{18}O$) requires *m/z* 363.2164, found *m/z* 363.2160.

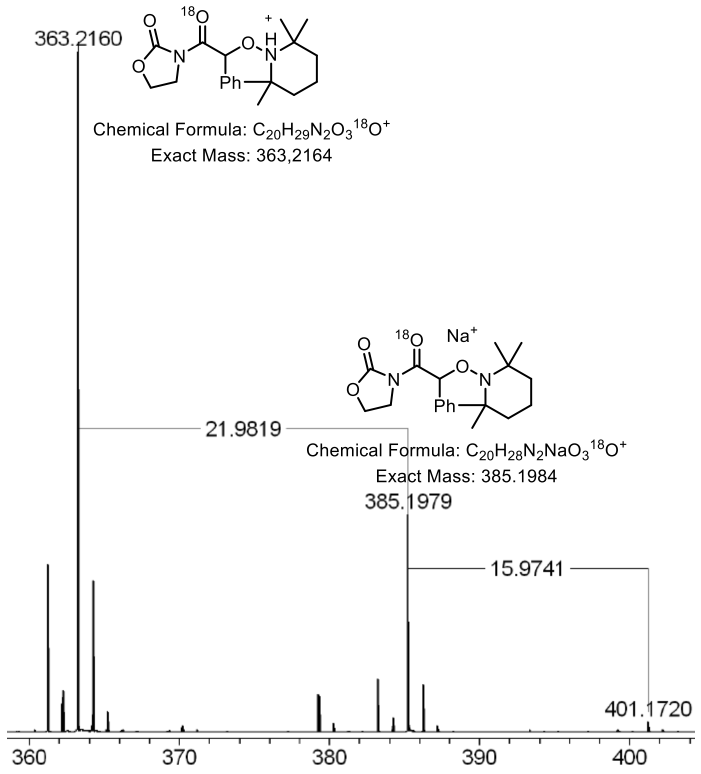

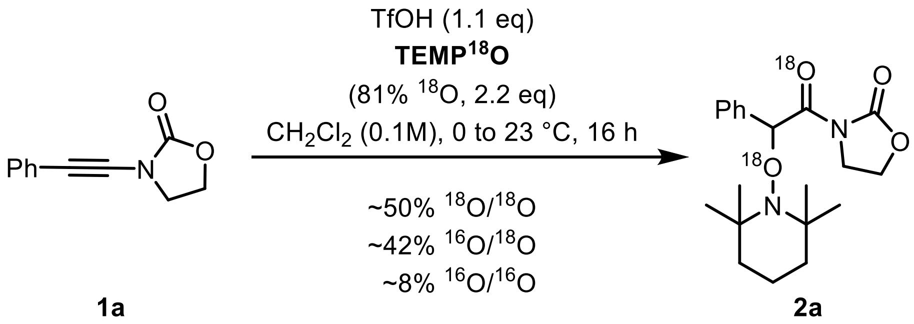

To a solution of ynamide **1a** (1.0 equiv.) in dichloromethane (0.1 M) at 0 °C was added trifluoromethanesulfonic acid (1.1 equiv.). After 15 minutes at 0 °C, TEMP^{18}O (2.20 equiv.)[17] was added in one portion and the resulting reaction mixture was allowed to warm to ambient temperature over the course of 16 h. After this, H_2O was added, the resulting biphasic mixture was diluted with dichloromethane and the phases were separated. The aqueous phase was extracted with dichloromethane and the combined organic phases were dried over anhydrous sodium sulfate. The dried solution was filtered and the filtrate was concentrated under reduced pressure on a rotary evaporator. HRMS-analysis of the crude mixture indicated that isotopic distribution amounted to approximately 50% ^{18}O/^{18}O, 42% ^{16}O/^{18}O and 8% ^{16}O/^{16}O. HRMS-analysis: exact mass calculated for $[M+H]^+$ ($C_{20}H_{29}O_2N_2{}^{18}O_2$) requires *m/z* 365.2207, found *m/z* 365.2205.

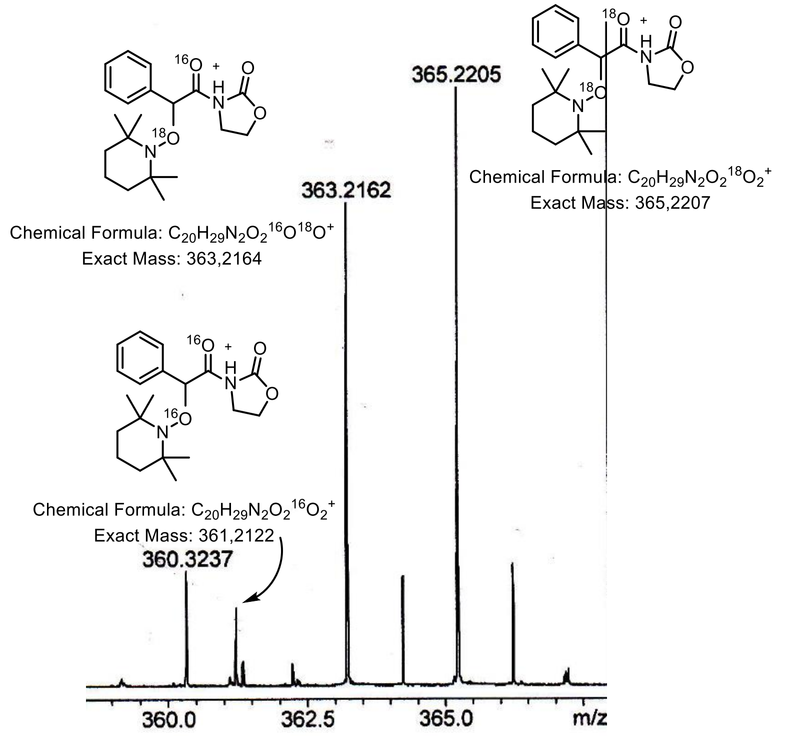

[17] Prepared following the procedure reported in: V. A. Golubev, V. D. Sen', É. G. Rozantsev, *Russ. Chem. Bull.* **1979**, *28*, 1927.

6. NMR-Spectra

6.1. Ynamides

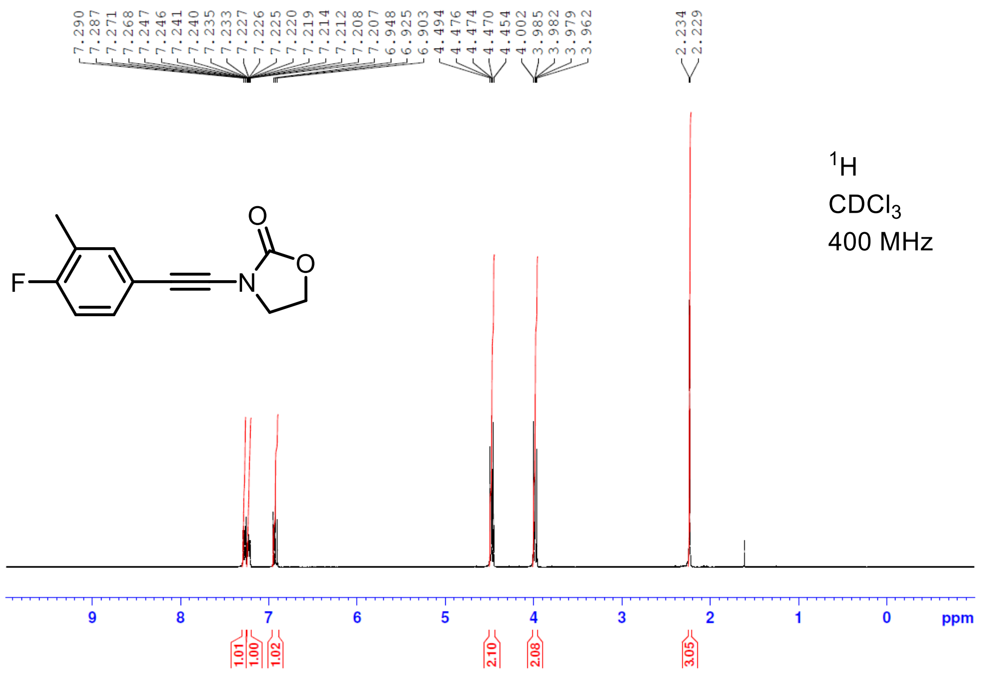

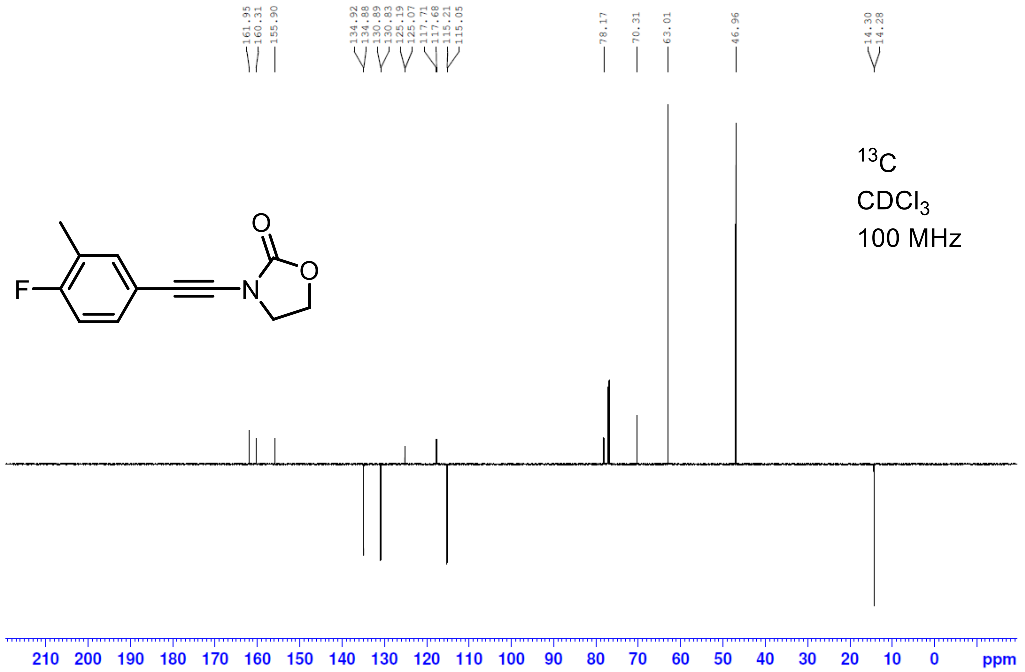

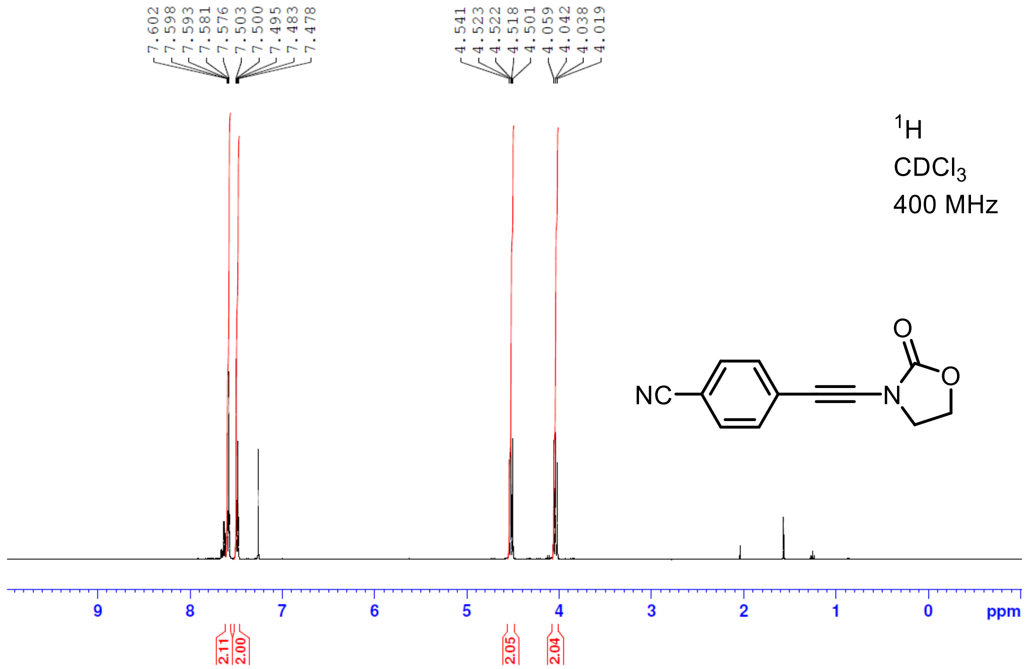
7.602
7.598
7.593
7.581
7.576
7.503
7.500
7.495
7.483
7.478
4.541
4.523
4.522
4.518
4.501
4.059
4.042
4.038
4.019
^{1}H
$CDCl_3$
400 MHz
NC
N
O
O
9
8
7
6
5
4
3
2
1
0
ppm
2.11
2.00
2.05
2.04

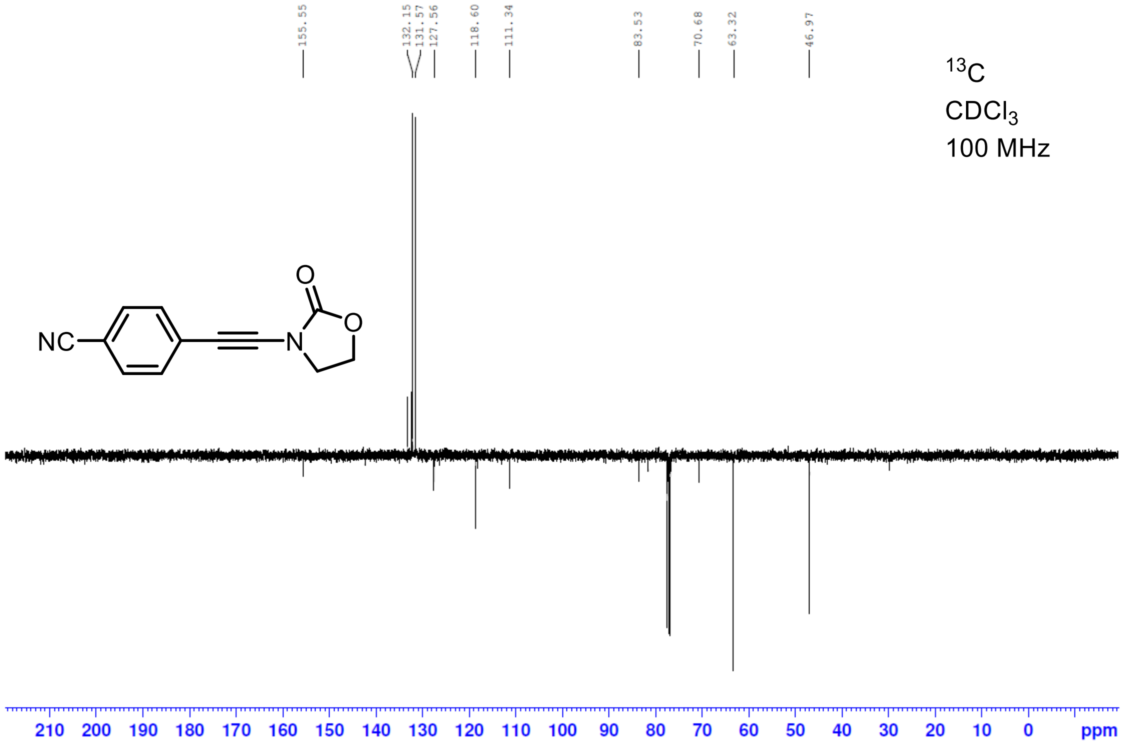
155.55
132.15
131.57
127.56
118.60
111.34
83.53
70.68
63.32
46.97
^{13}C
$CDCl_3$
100 MHz
NC
N
O
O
210
200
190
180
170
160
150
140
130
120
110
100
90
80
70
60
50
40
30
20
10
0
ppm

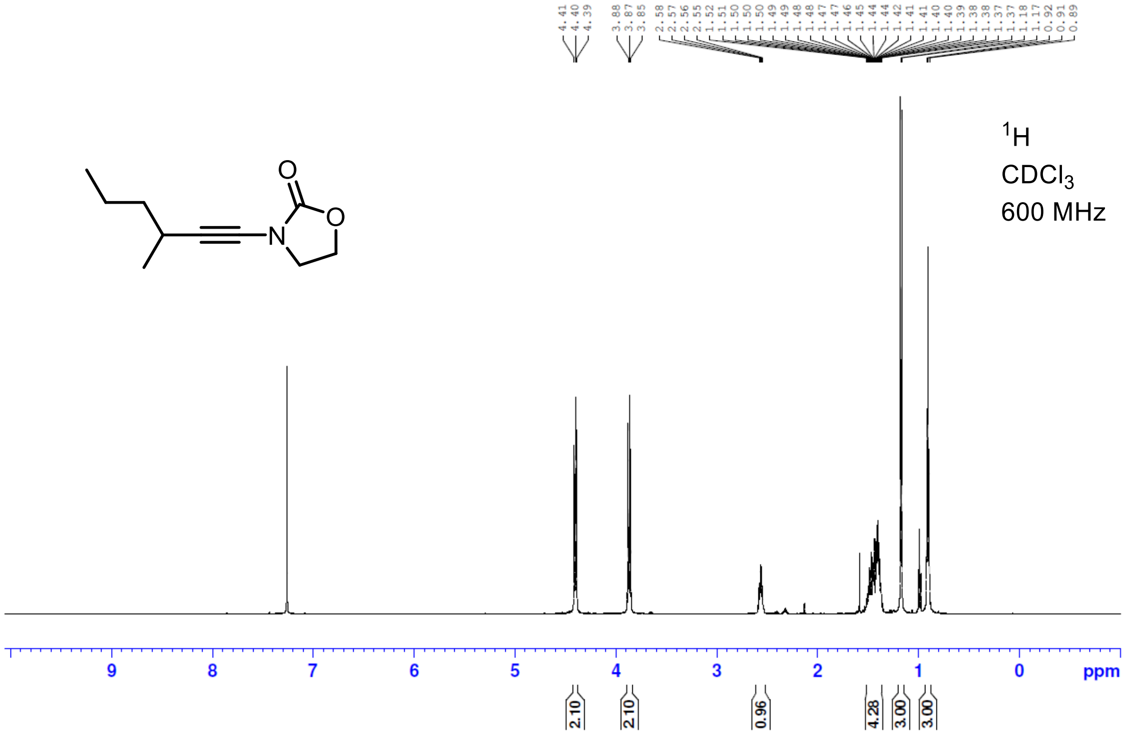
1H
CDCl3
600 MHz
ppm
2.10
2.10
0.96
4.28
3.00
3.00

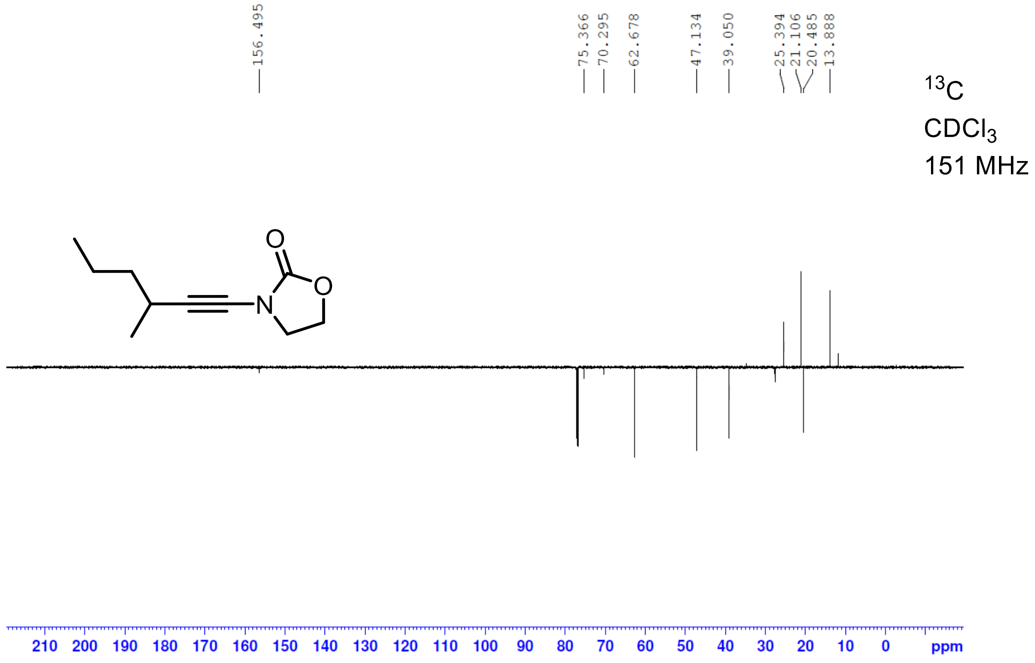
156.495
75.366
70.295
62.678
47.134
39.050
25.394
21.106
20.485
13.888
13C
CDCl3
151 MHz
ppm

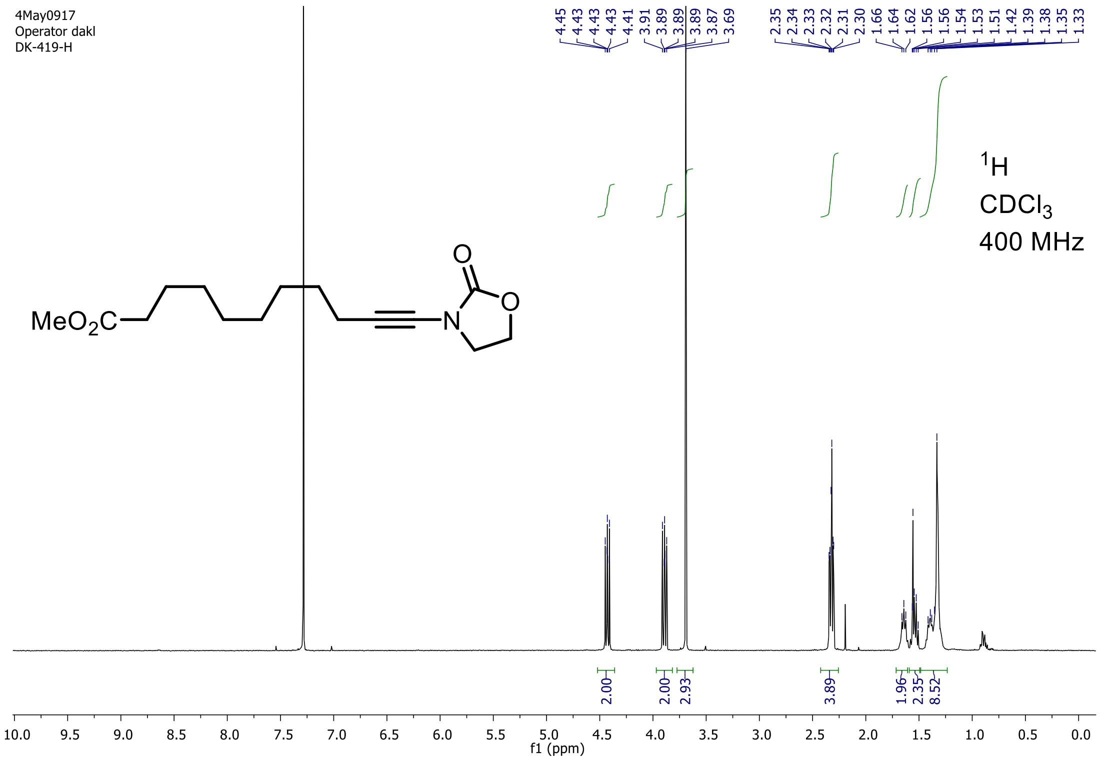
4May0917
Operator dakl
DK-419-H
4.45 4.43 4.43 4.43 4.41 3.91 3.89 3.89 3.89 3.87 3.69 2.35 2.34 2.33 2.32 2.31 2.30 1.66 1.64 1.62 1.56 1.56 1.54 1.53 1.51 1.42 1.39 1.38 1.35 1.33
^{1}H
$CDCl_3$
400 MHz
MeO_2C
N
O
O
2.00 2.00 2.93 3.89 1.96 2.35 8.52
10.0 9.5 9.0 8.5 8.0 7.5 7.0 6.5 6.0 5.5 5.0 4.5 4.0 3.5 3.0 2.5 2.0 1.5 1.0 0.5 0.0
f1 (ppm)

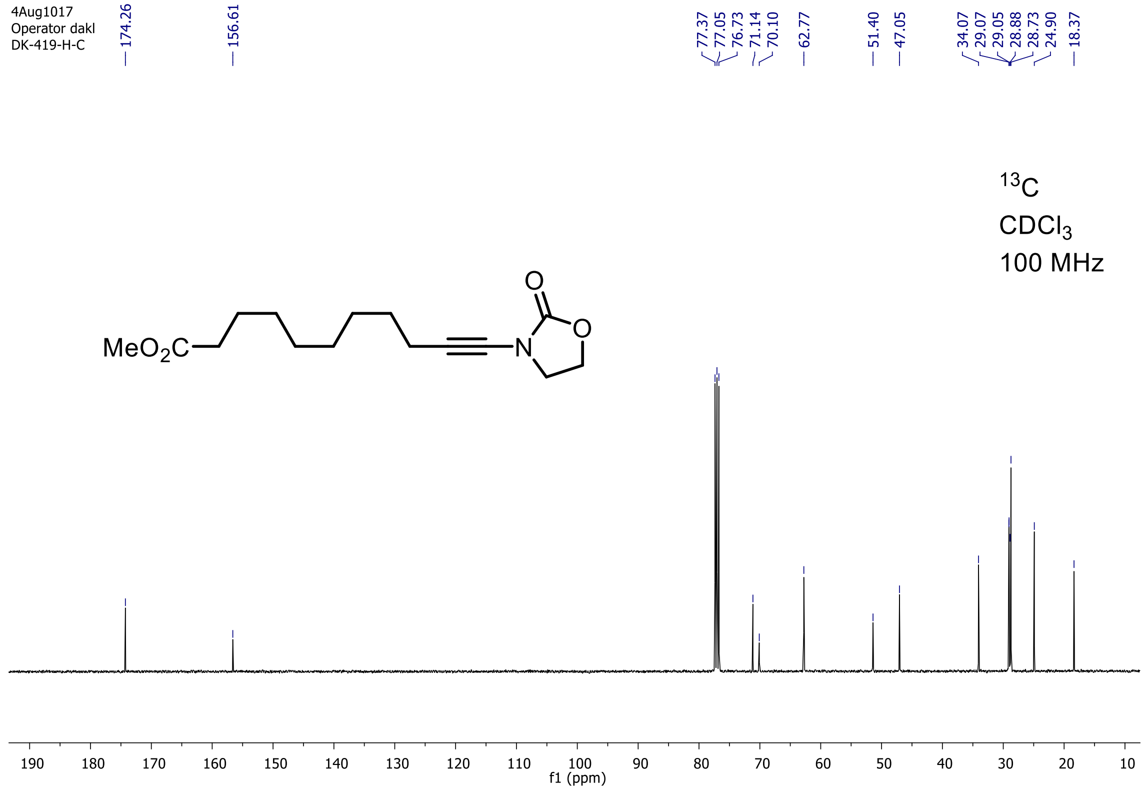
4Aug1017
Operator dakl
DK-419-H-C
174.26
156.61
77.37 77.05 76.73 71.14 70.10
62.77
51.40
47.05
34.07 29.07 29.05 28.88 28.73 24.90 18.37
^{13}C
$CDCl_3$
100 MHz
MeO_2C
N
O
O
190 180 170 160 150 140 130 120 110 100 90 80 70 60 50 40 30 20 10
f1 (ppm)

6.2. Products

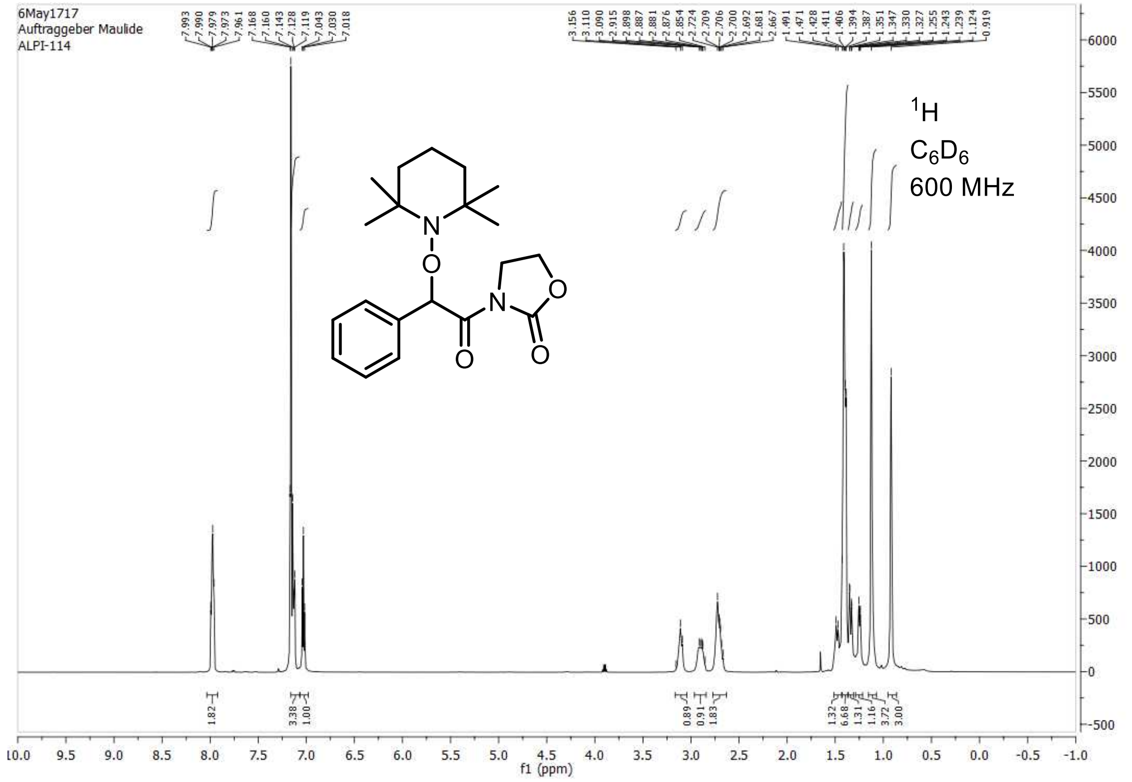

6May1717
Auftraggeber Maulide
ALPI-114
1H
C6D6
600 MHz
f1 (ppm)

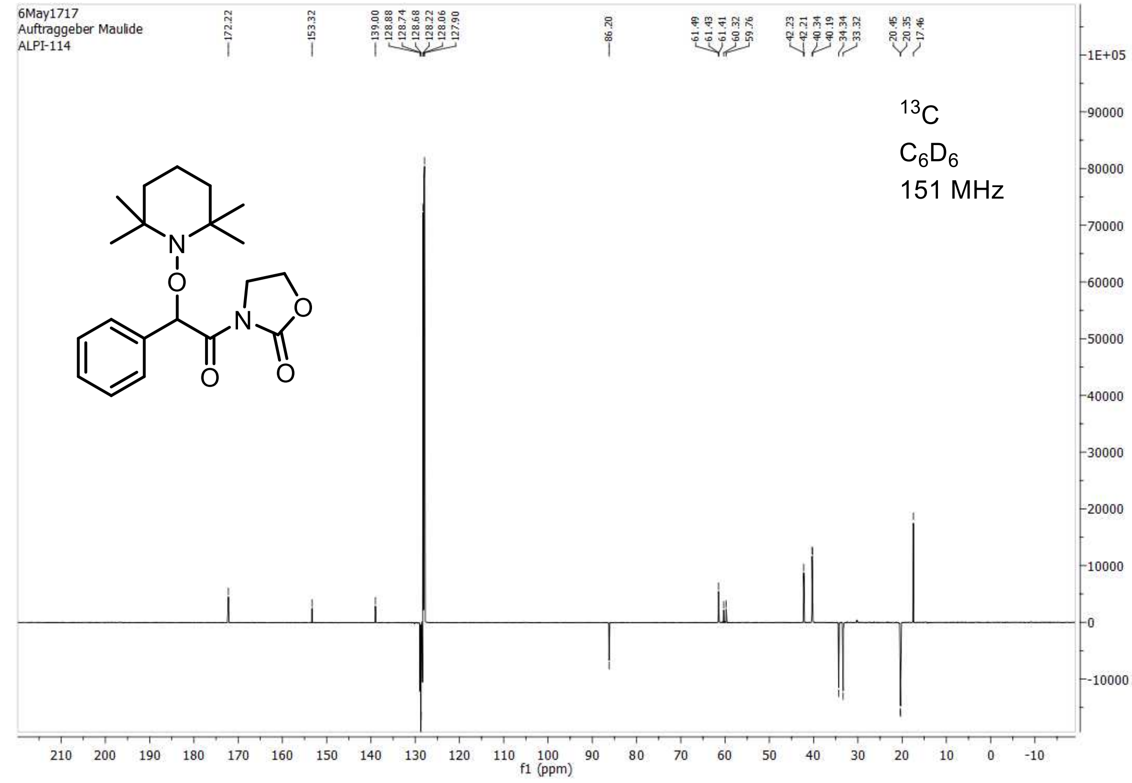

6May1717
Auftraggeber Maulide
ALPI-114
13C
C6D6
151 MHz
f1 (ppm)

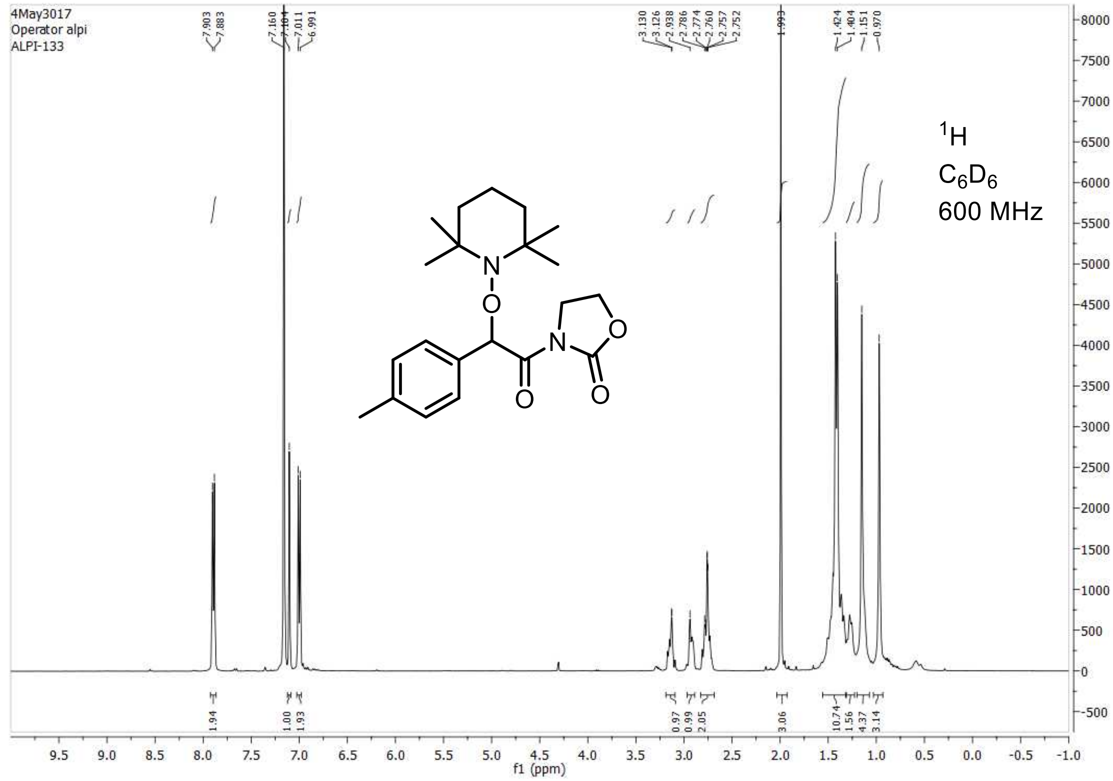
4May3017
Operator alpi
ALPI-133
1H
C6D6
600 MHz
f1 (ppm)

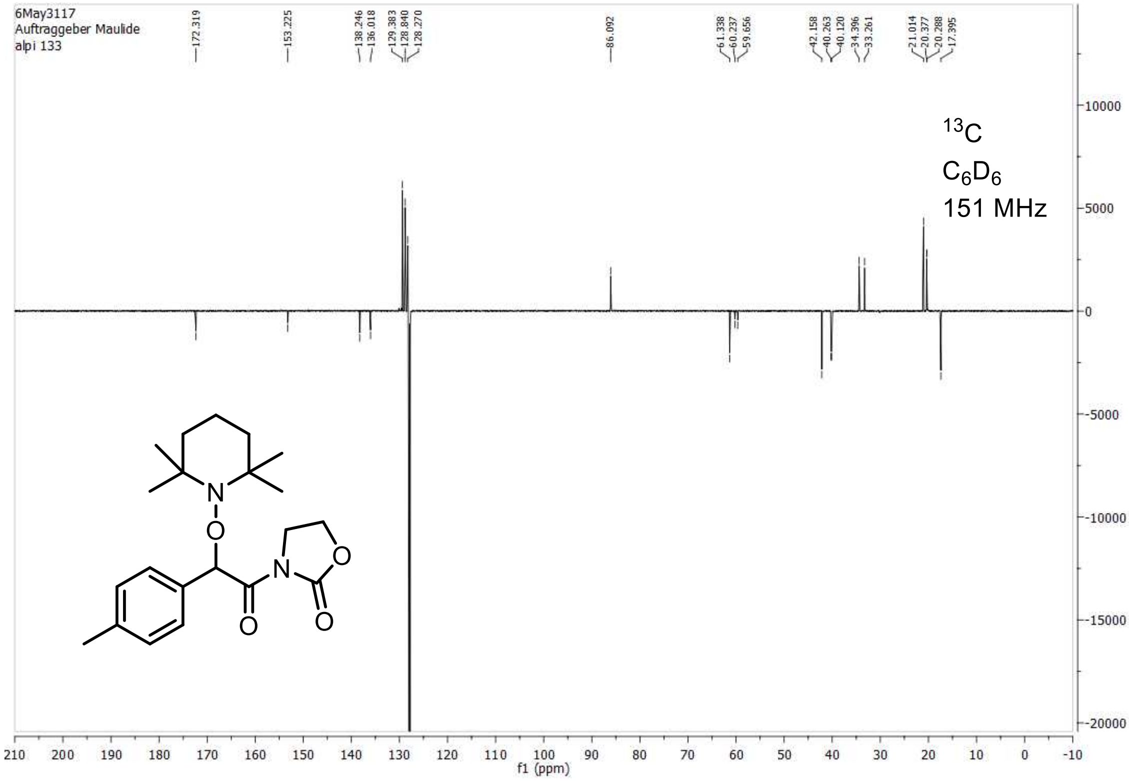
6May3117
Auftraggeber Maulide
alpi 133
13C
C6D6
151 MHz
f1 (ppm)

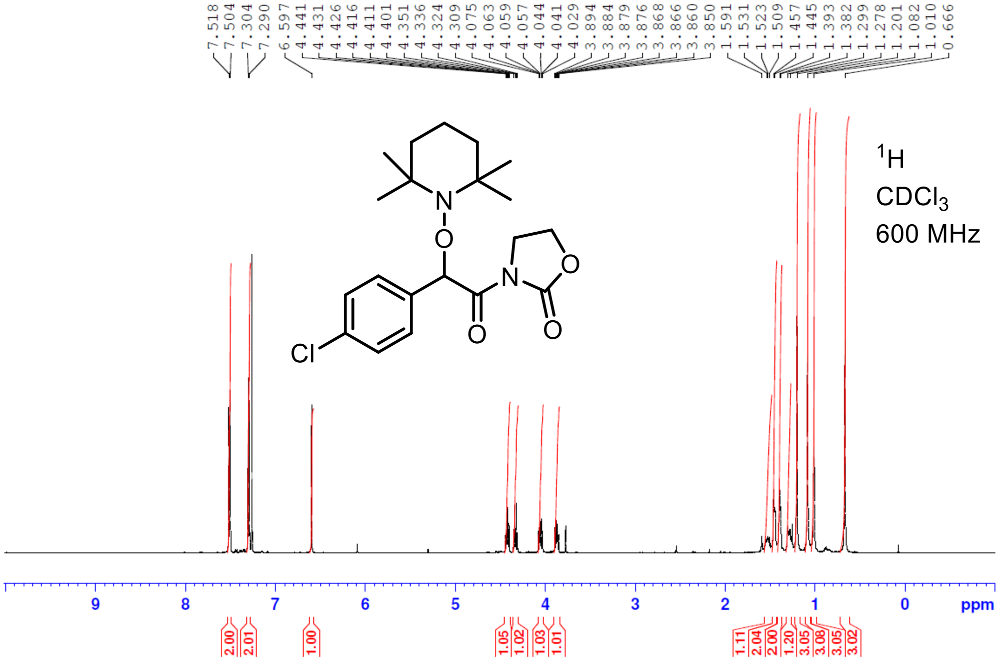
7.518
7.504
7.304
7.290
6.597
4.441
4.431
4.426
4.416
4.411
4.401
4.351
4.336
4.324
4.309
4.075
4.063
4.059
4.057
4.044
4.041
4.029
3.894
3.884
3.879
3.876
3.868
3.866
3.860
3.850
1.591
1.531
1.523
1.509
1.457
1.445
1.393
1.382
1.299
1.278
1.201
1.082
1.010
0.666
^1H
$CDCl_3$
600 MHz
Cl
N
O
9
8
7
6
5
4
3
2
1
0
ppm
2.00
2.01
1.00
1.05
1.02
1.03
1.01
1.11
2.04
2.00
1.20
3.05
3.08
3.05
3.02

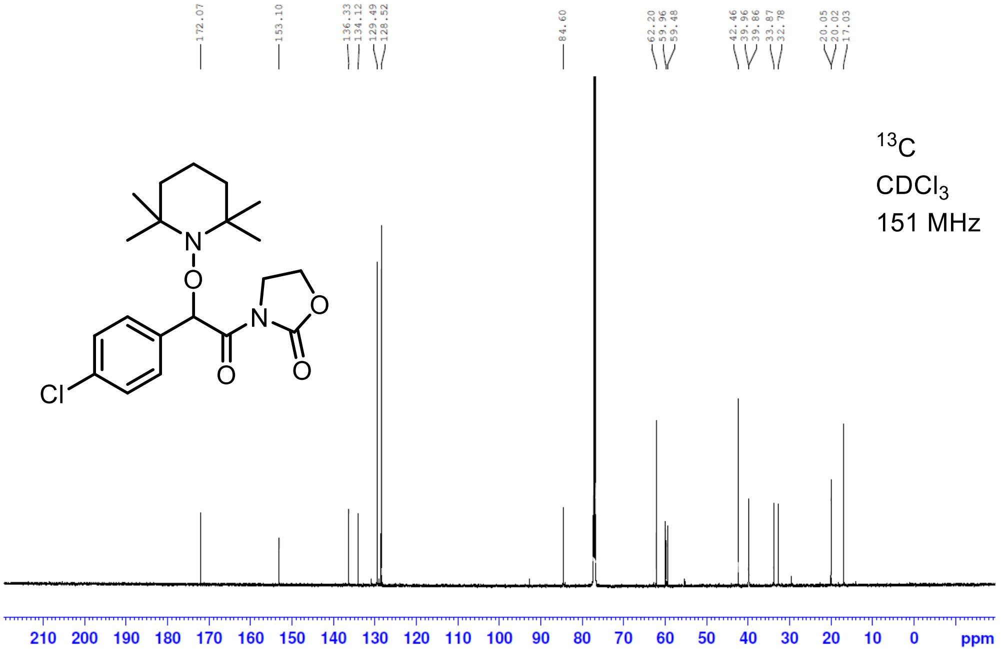
172.07
153.10
136.33
134.12
129.49
128.52
84.60
62.20
59.96
59.48
42.46
39.96
39.86
33.87
32.78
20.05
20.02
17.03
^{13}C
$CDCl_3$
151 MHz
Cl
N
O
210
200
190
180
170
160
150
140
130
120
110
100
90
80
70
60
50
40
30
20
10
0
ppm

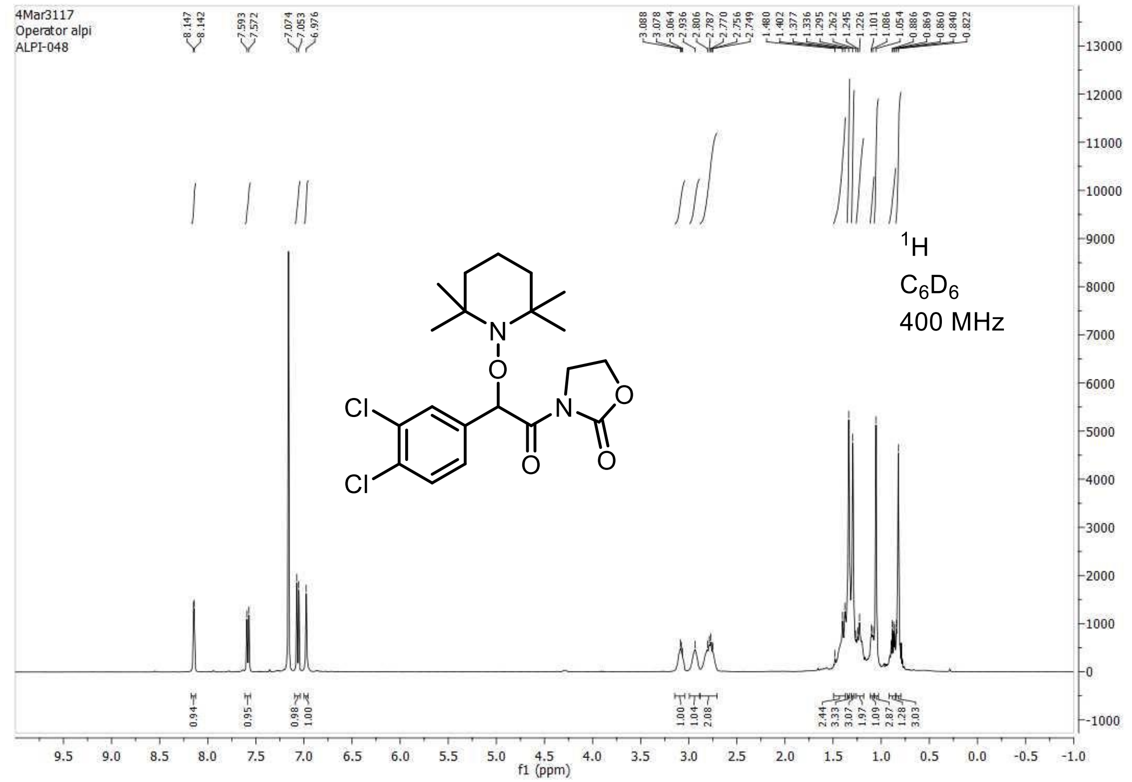

4Mar3117
Operator alpi
ALPI-048
1H
C6D6
400 MHz
Cl
Cl
N
O
O
N
O
O
f1 (ppm)

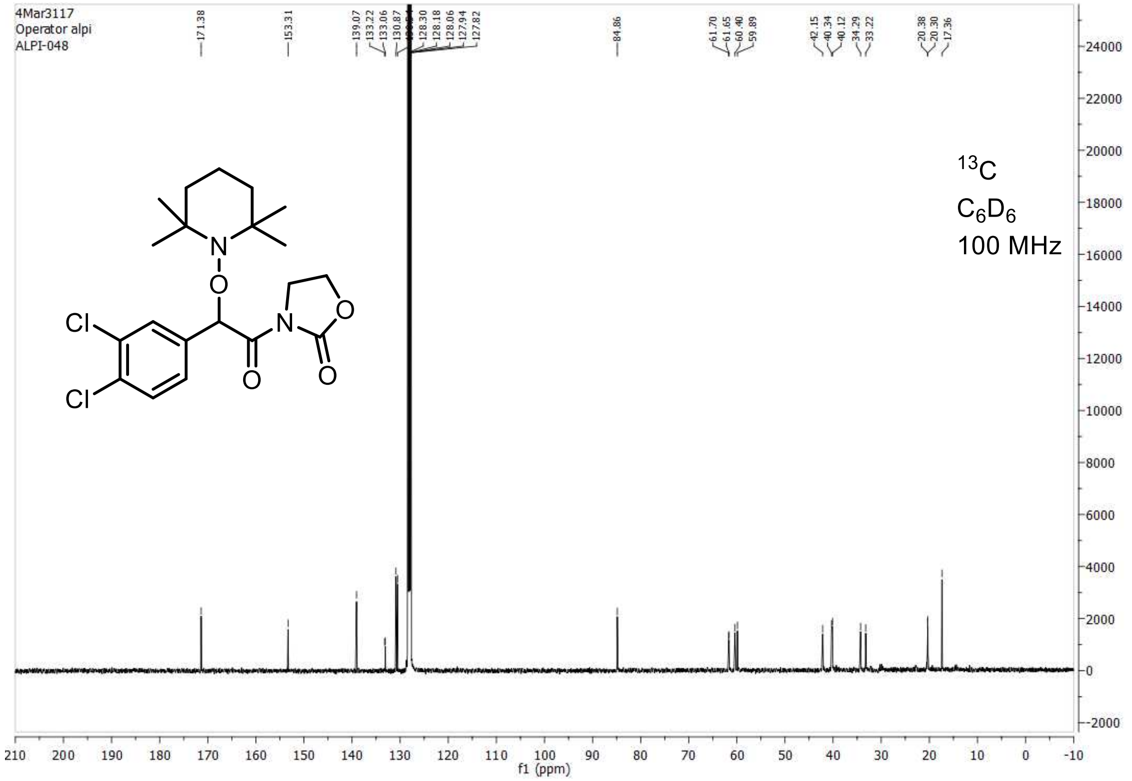

4Mar3117
Operator alpi
ALPI-048
13C
C6D6
100 MHz
Cl
Cl
N
O
O
N
O
O
f1 (ppm)

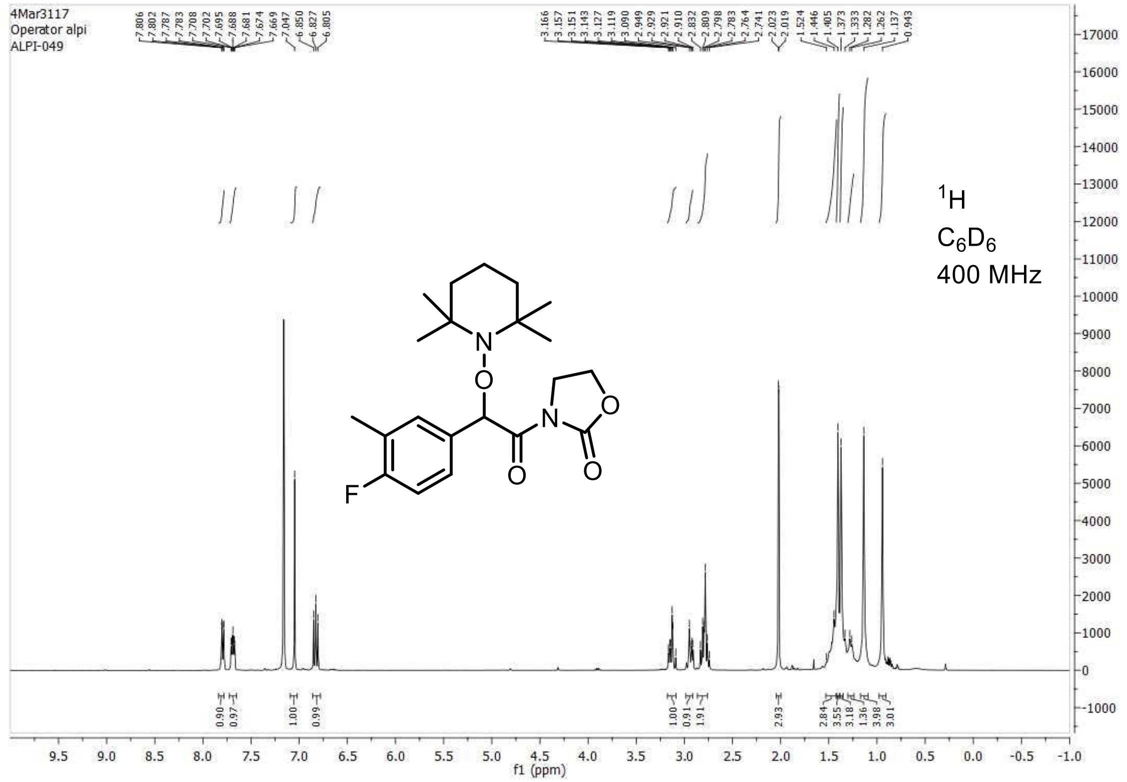
4Mar3117
Operator alpi
ALPI-049
1H
C6D6
400 MHz
f1 (ppm)
F
N
O

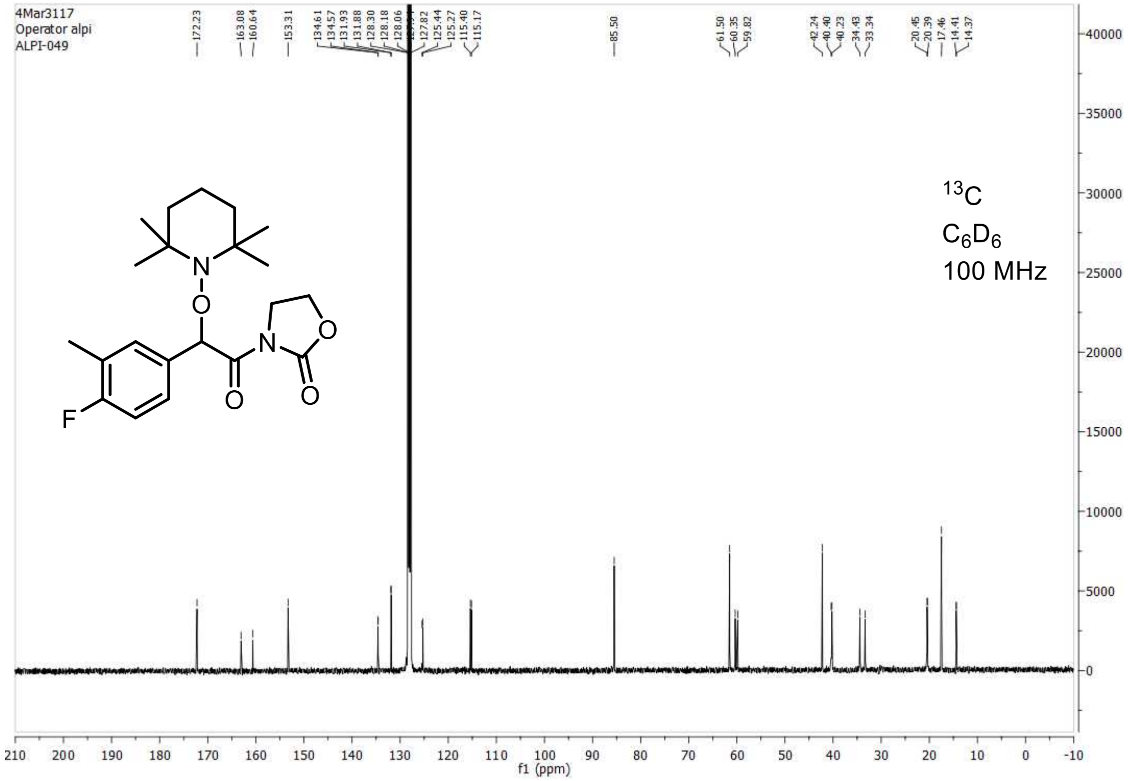
4Mar3117
Operator alpi
ALPI-049
13C
C6D6
100 MHz
f1 (ppm)
F
N
O

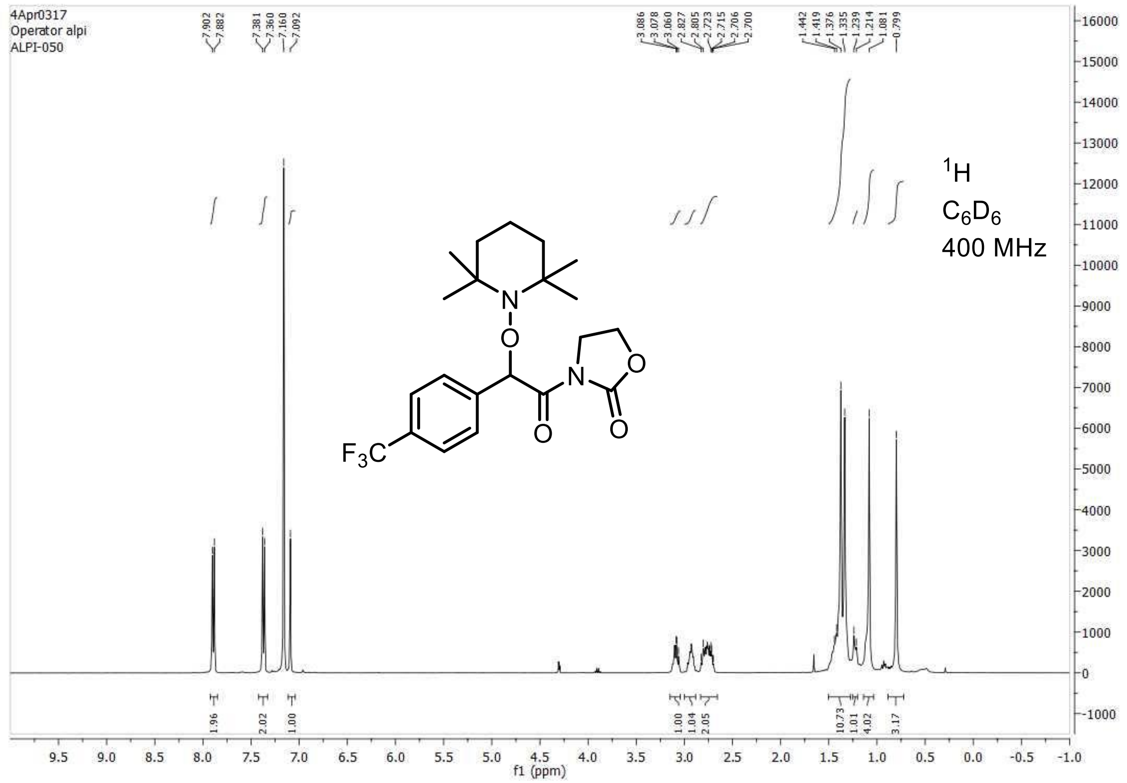
4Apr0317
Operator alpi
ALPI-050
¹H
C6D6
400 MHz
F3C
f1 (ppm)

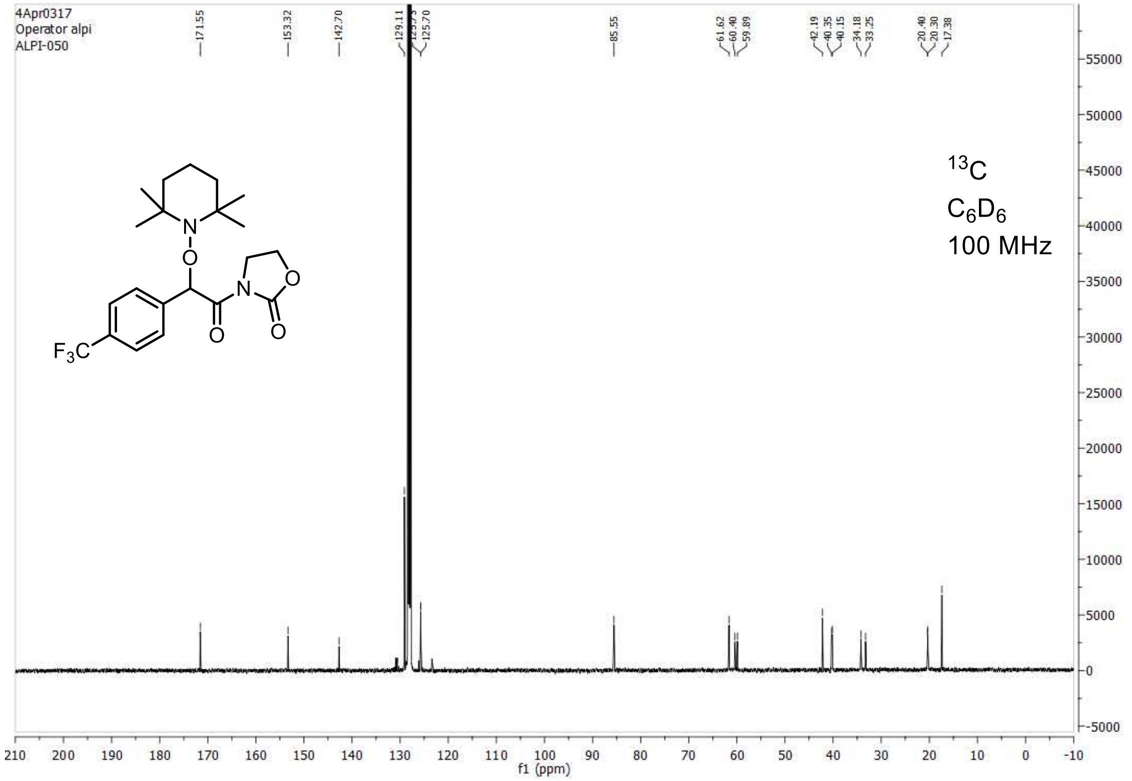
4Apr0317
Operator alpi
ALPI-050
¹³C
C6D6
100 MHz
F3C
f1 (ppm)

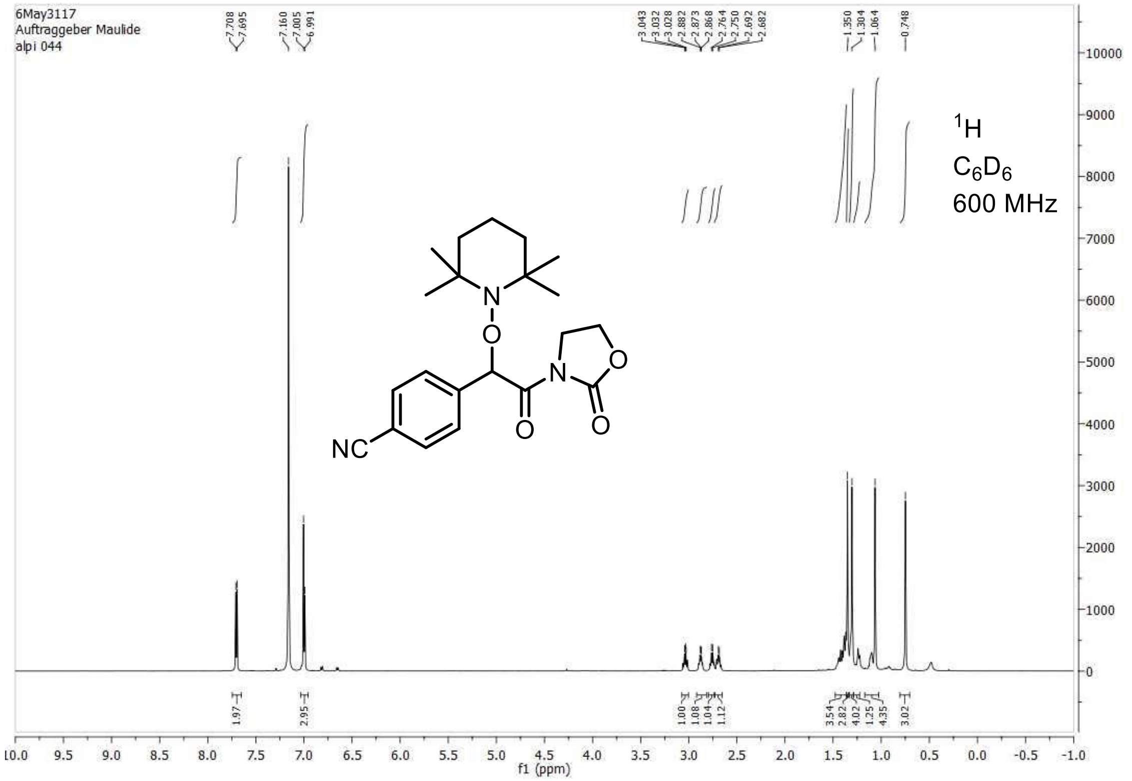

6May3117
Auftraggeber Maulide
alpi 044
1H
C6D6
600 MHz
NC
N
O
O
N
O
O
f1 (ppm)

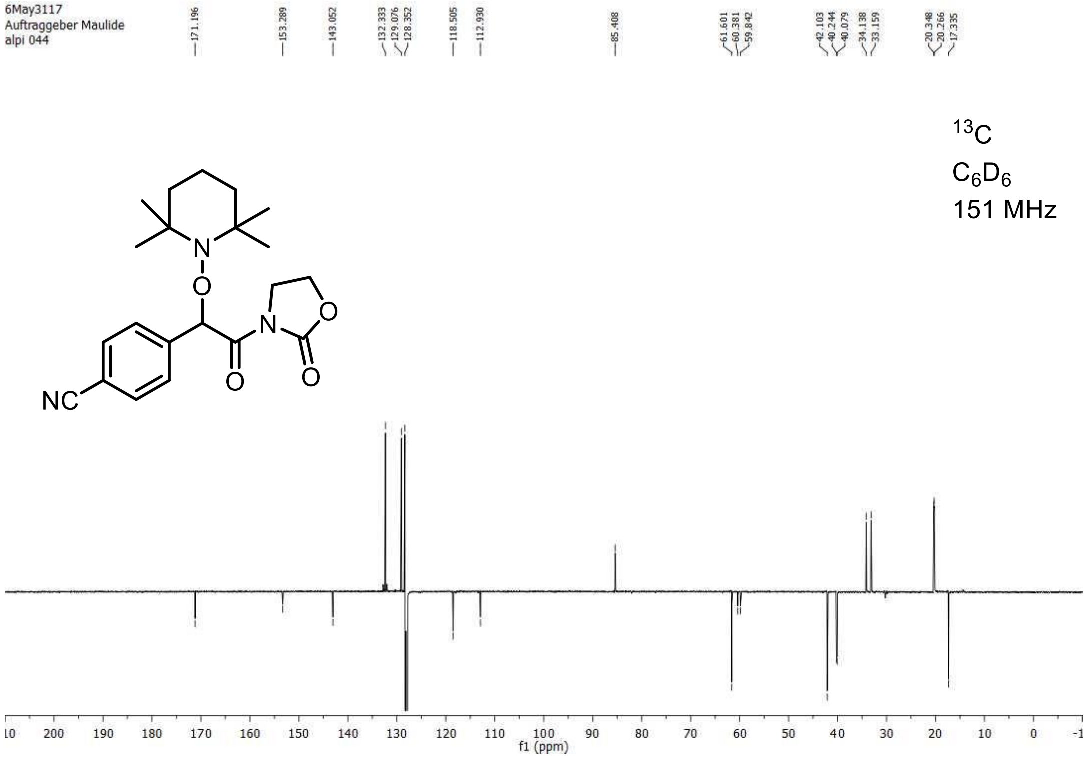

6May3117
Auftraggeber Maulide
alpi 044
13C
C6D6
151 MHz
NC
N
O
O
N
O
O
f1 (ppm)

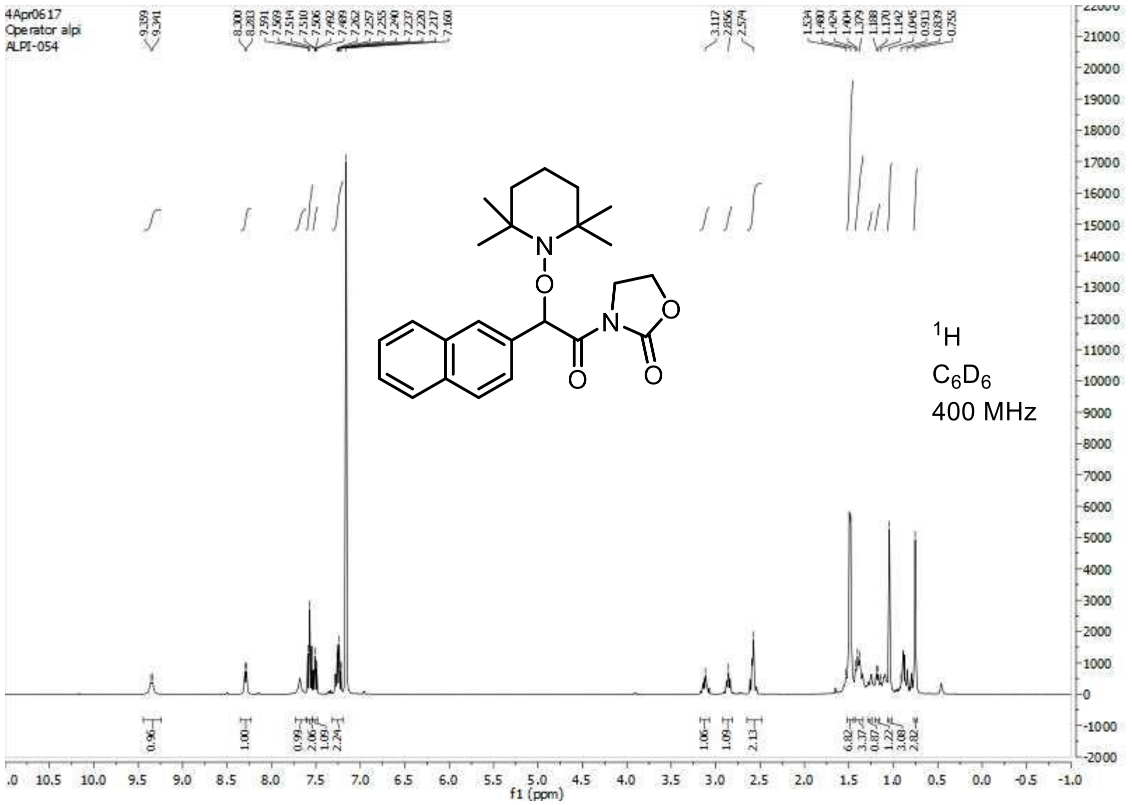
4Apr0617
Operator alpi
ALPI-054
1H
C6D6
400 MHz
f1 (ppm)

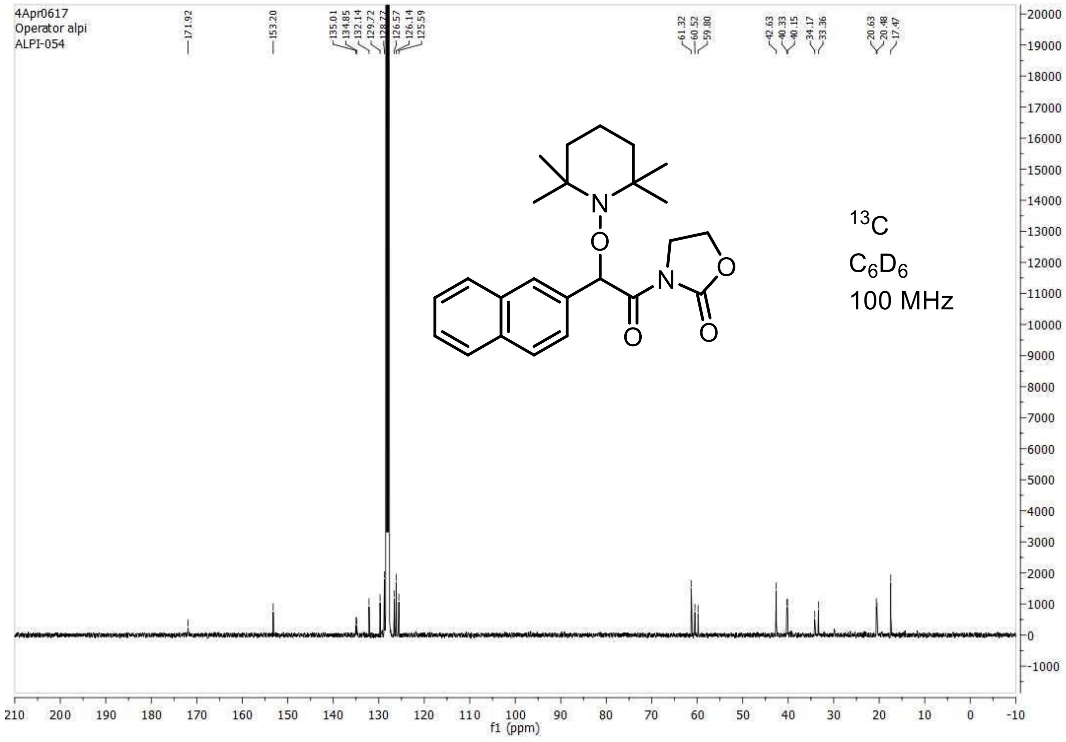
4Apr0617
Operator alpi
ALPI-054
13C
C6D6
100 MHz
f1 (ppm)

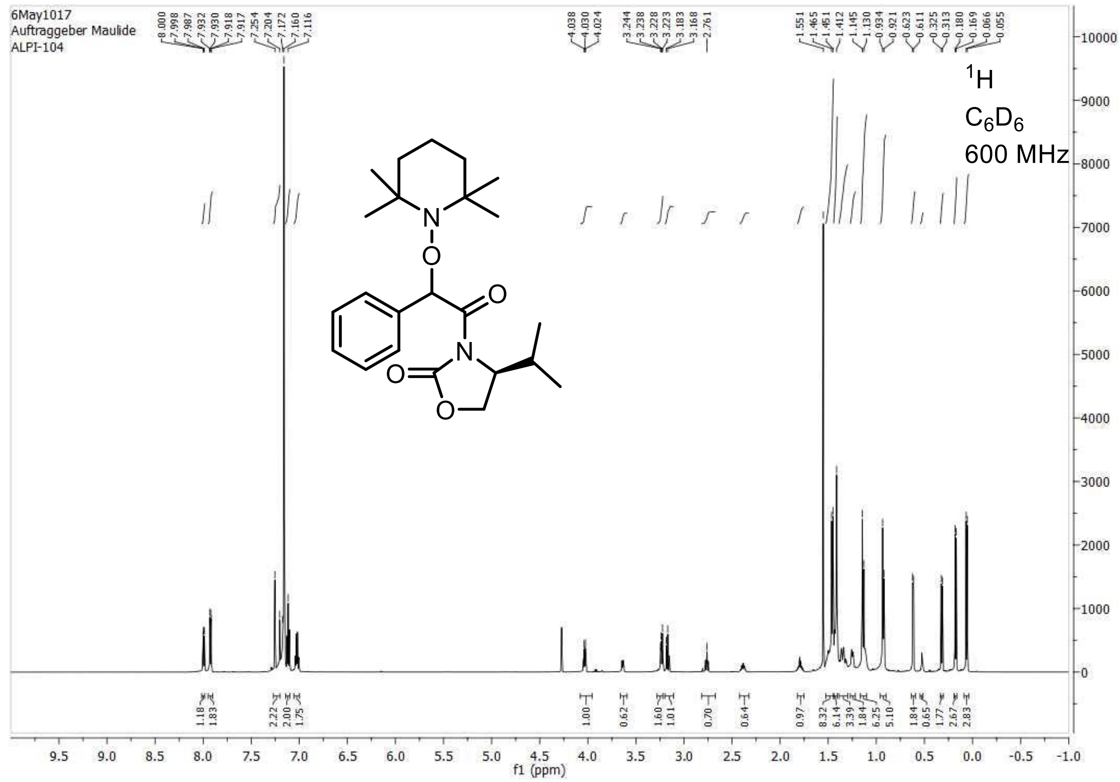
6May1017
Auftraggeber Maulide
ALPI-104
^{1}H
C_6D_6
600 MHz
f1 (ppm)

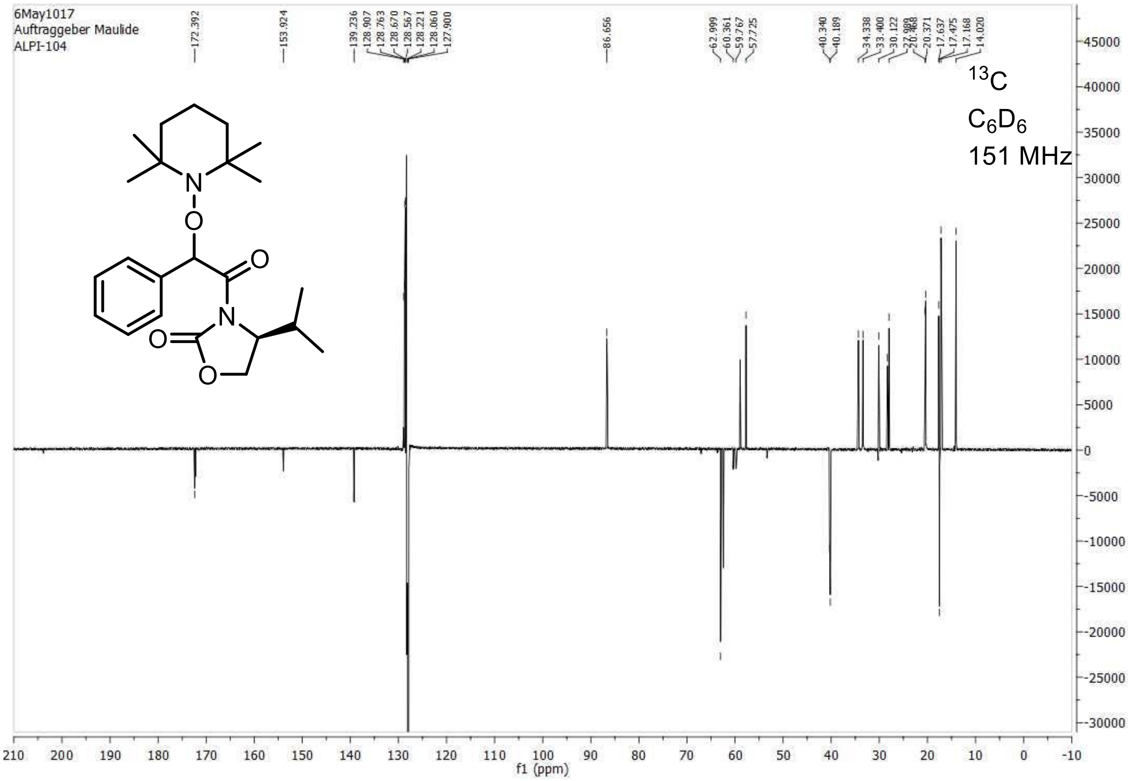
6May1017
Auftraggeber Maulide
ALPI-104
^{13}C
C_6D_6
151 MHz
f1 (ppm)

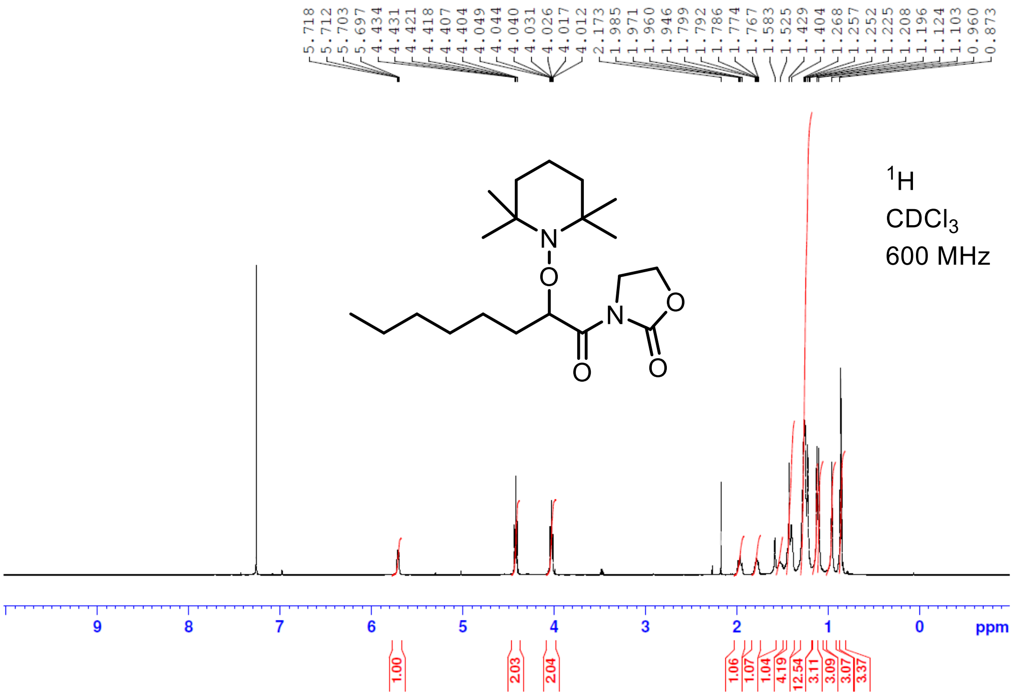

5.718
5.712
5.703
5.697
4.434
4.431
4.421
4.418
4.407
4.404
4.049
4.044
4.040
4.031
4.026
4.017
4.012
2.173
1.985
1.971
1.960
1.946
1.799
1.792
1.786
1.774
1.767
1.583
1.525
1.429
1.404
1.268
1.257
1.252
1.225
1.208
1.196
1.124
1.103
0.960
0.873
N
O
O
N
O
O
O
[1]H
$CDCl_3$
600 MHz
9
8
7
6
5
4
3
2
1
0
ppm
1.00
2.03
2.04
1.06
1.07
1.04
4.19
12.54
3.11
3.09
3.07
3.37

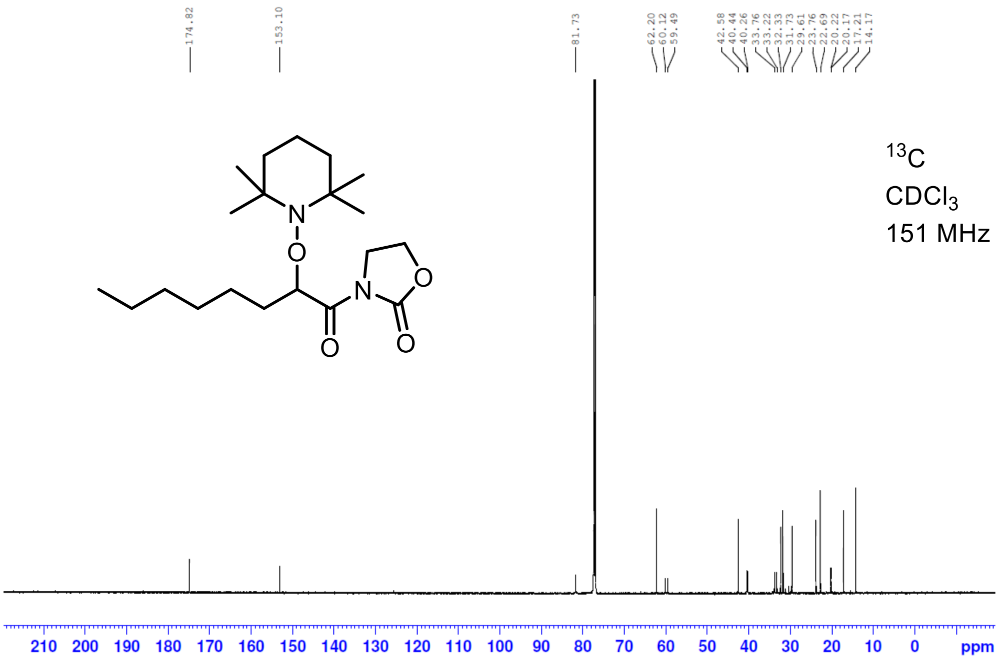

174.82
153.10
81.73
62.20
60.12
59.49
42.58
40.44
40.26
33.76
33.22
32.33
31.73
29.61
23.76
22.69
20.22
20.17
17.21
14.17
N
O
O
N
O
O
O
[13]C
$CDCl_3$
151 MHz
210
200
190
180
170
160
150
140
130
120
110
100
90
80
70
60
50
40
30
20
10
0
ppm

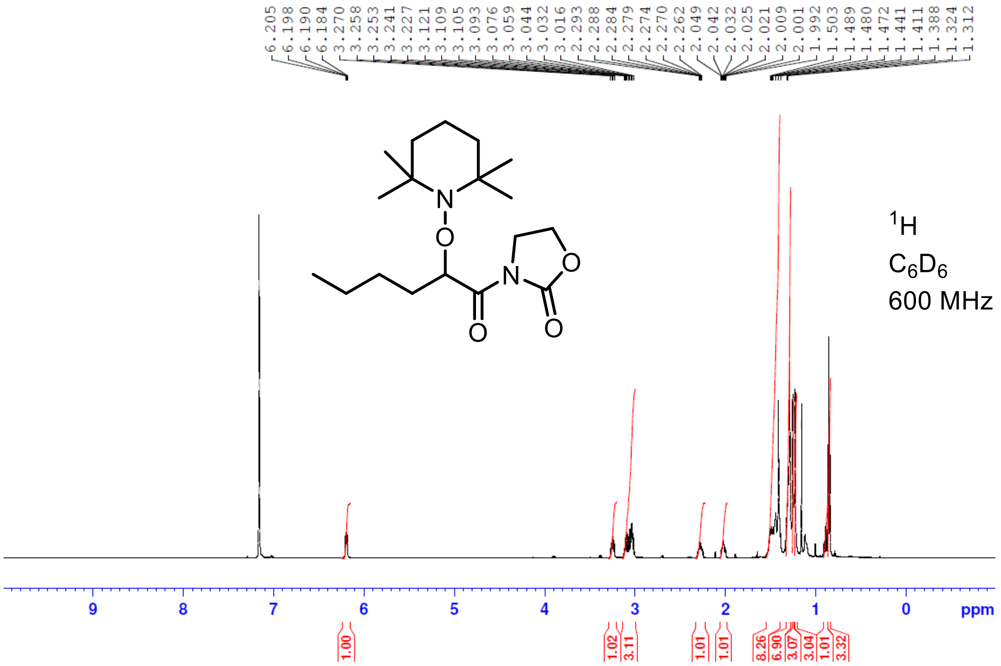
6.205
6.198
6.190
6.184
3.270
3.258
3.253
3.241
3.227
3.121
3.109
3.105
3.093
3.076
3.059
3.044
3.032
3.016
2.293
2.288
2.284
2.279
2.274
2.270
2.262
2.049
2.042
2.032
2.025
2.021
2.009
2.001
1.992
1.503
1.489
1.480
1.472
1.441
1.411
1.388
1.324
1.312
N
O
O
N
O
O
O
1H
C6D6
600 MHz
9 8 7 6 5 4 3 2 1 0 ppm
1.00
1.02
3.11
1.01
1.01
8.26
6.90
3.07
3.04
1.01
3.32

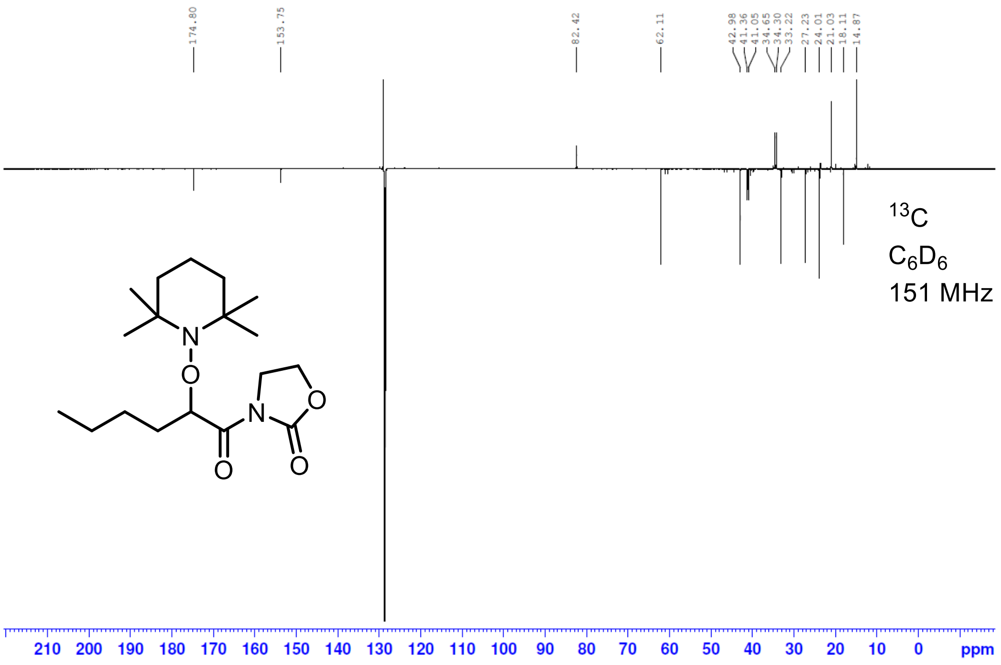
174.80
153.75
82.42
62.11
42.98
41.36
41.05
34.65
34.30
33.22
27.23
24.01
21.03
18.11
14.87
13C
C6D6
151 MHz
N
O
O
N
O
O
O
210 200 190 180 170 160 150 140 130 120 110 100 90 80 70 60 50 40 30 20 10 0 ppm

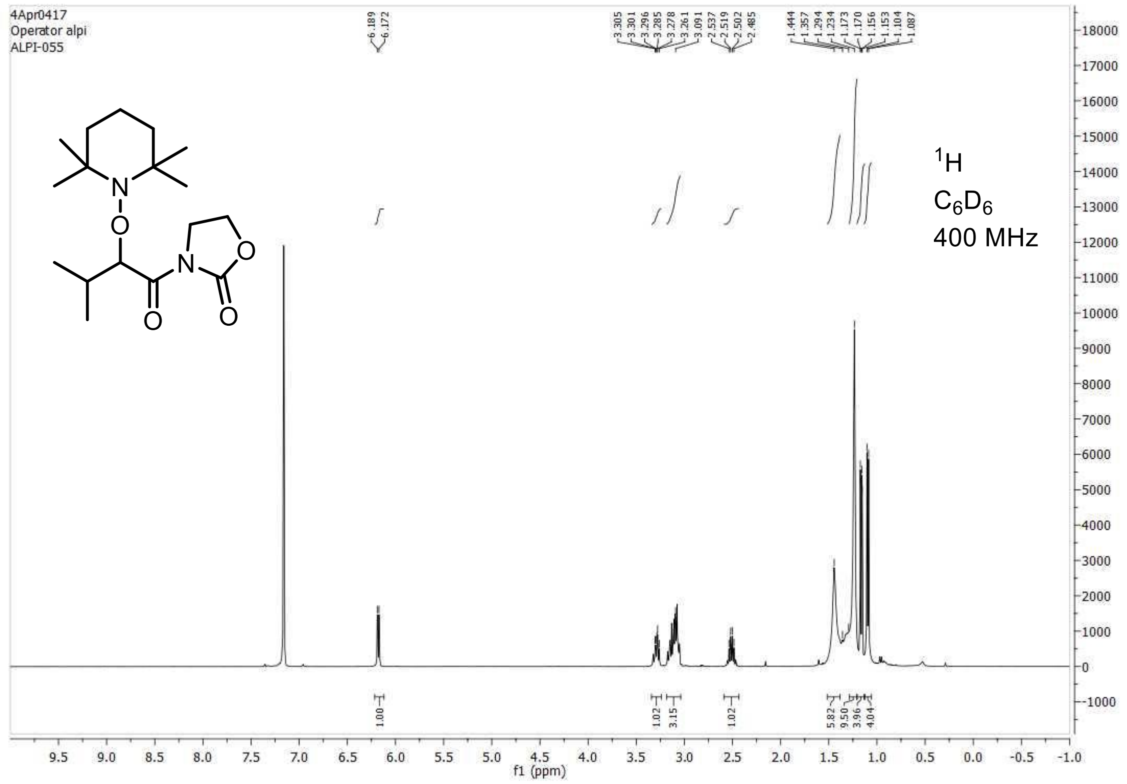
4Apr0417
Operator alpi
ALPI-055
N
O
O
N
O
O
O
^{1}H
C_6D_6
400 MHz
f1 (ppm)

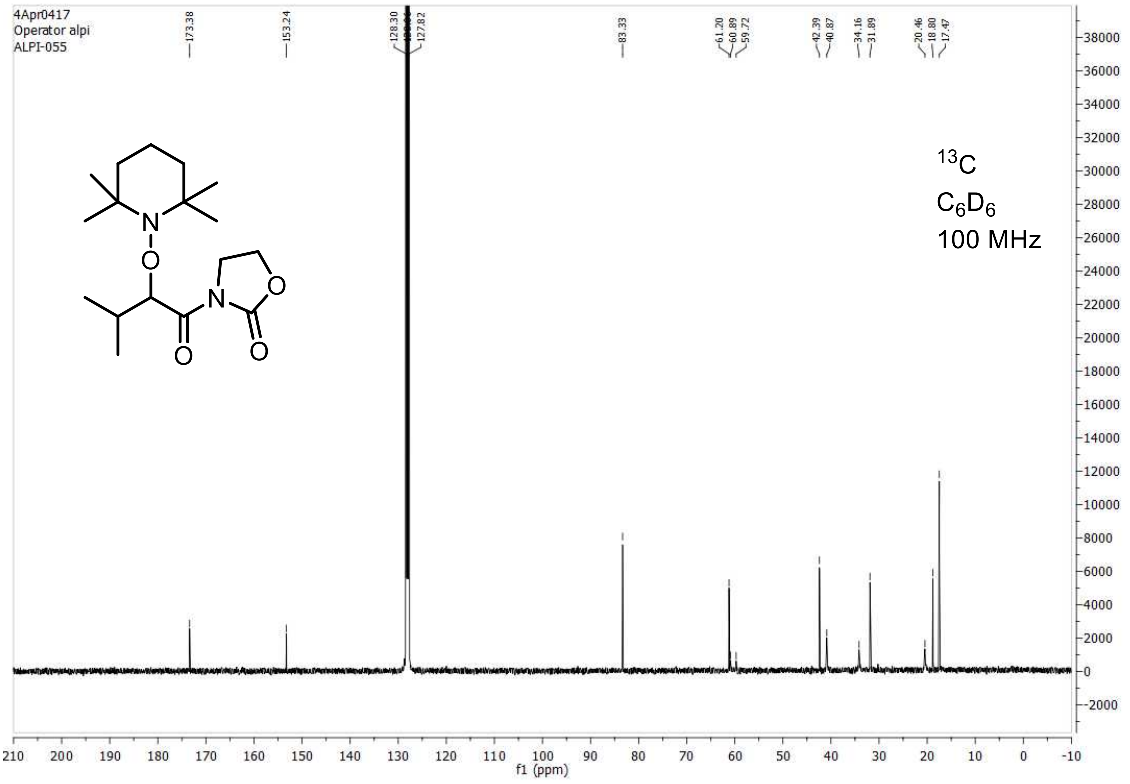
4Apr0417
Operator alpi
ALPI-055
N
O
O
N
O
O
O
^{13}C
C_6D_6
100 MHz
f1 (ppm)

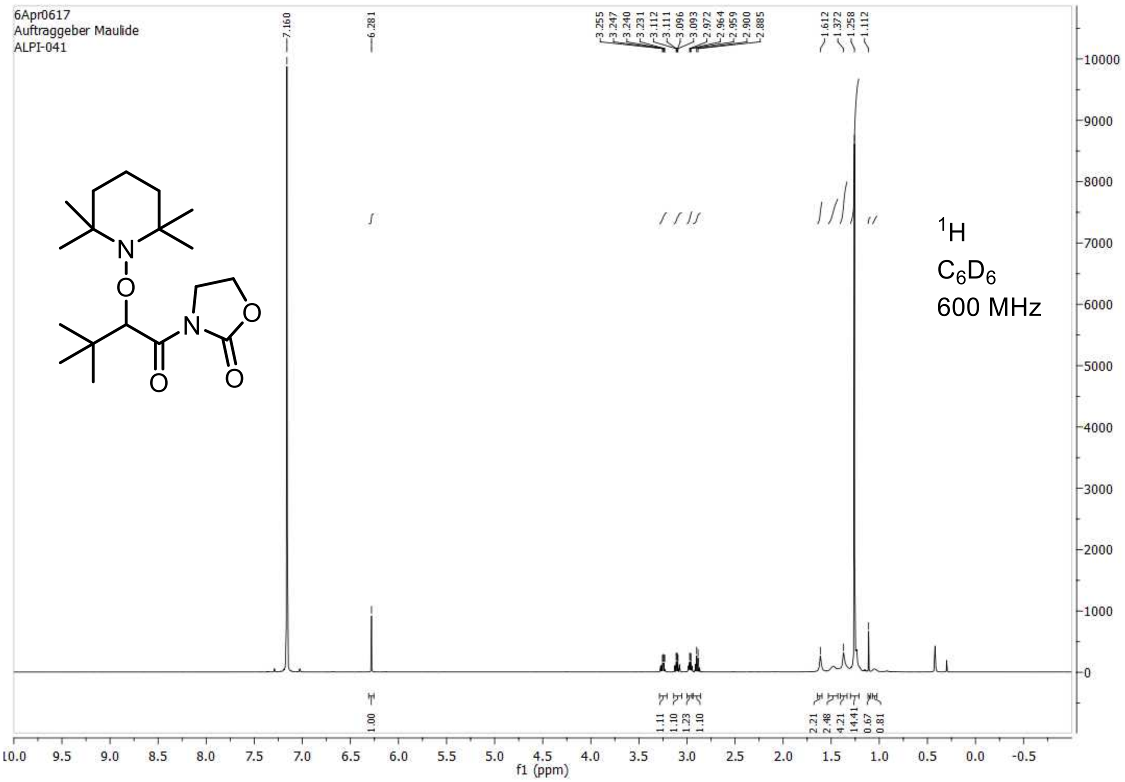
6Apr0617
Auftraggeber Maulide
ALPI-041
7.160
6.281
3.255
3.247
3.240
3.231
3.112
3.111
3.096
3.093
2.972
2.964
2.959
2.900
2.885
1.612
1.372
1.258
1.112
^{1}H
C_6D_6
600 MHz
1.00
1.11
1.10
1.23
1.10
2.21
2.48
4.21
14.41
0.67
0.81
f1 (ppm)

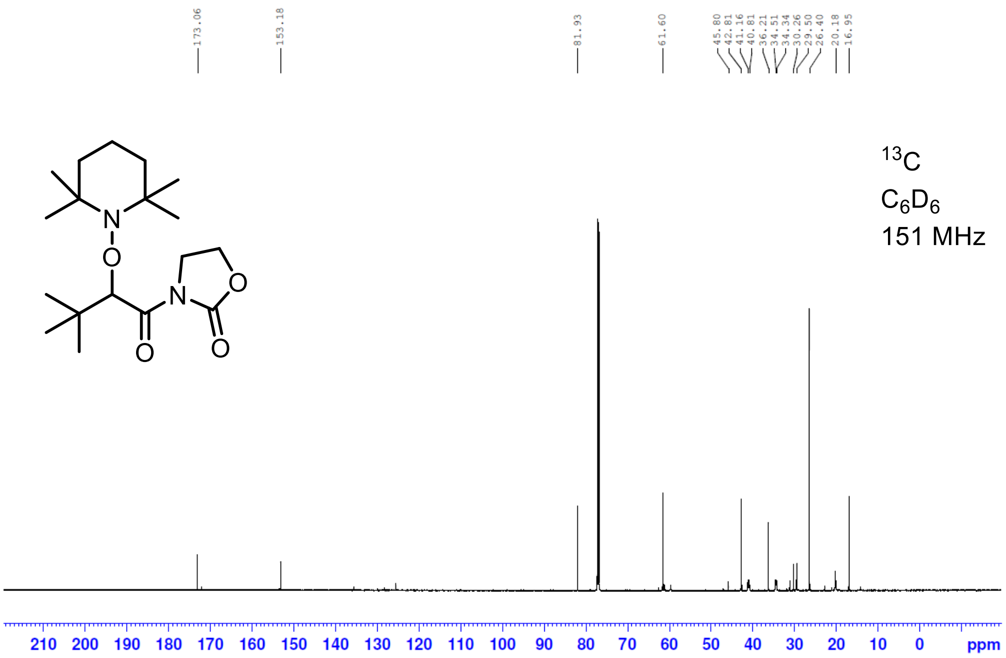
173.06
153.18
81.93
61.60
45.80
42.81
41.16
40.81
36.21
34.51
34.34
30.26
29.50
26.40
20.18
16.95
^{13}C
C_6D_6
151 MHz
ppm

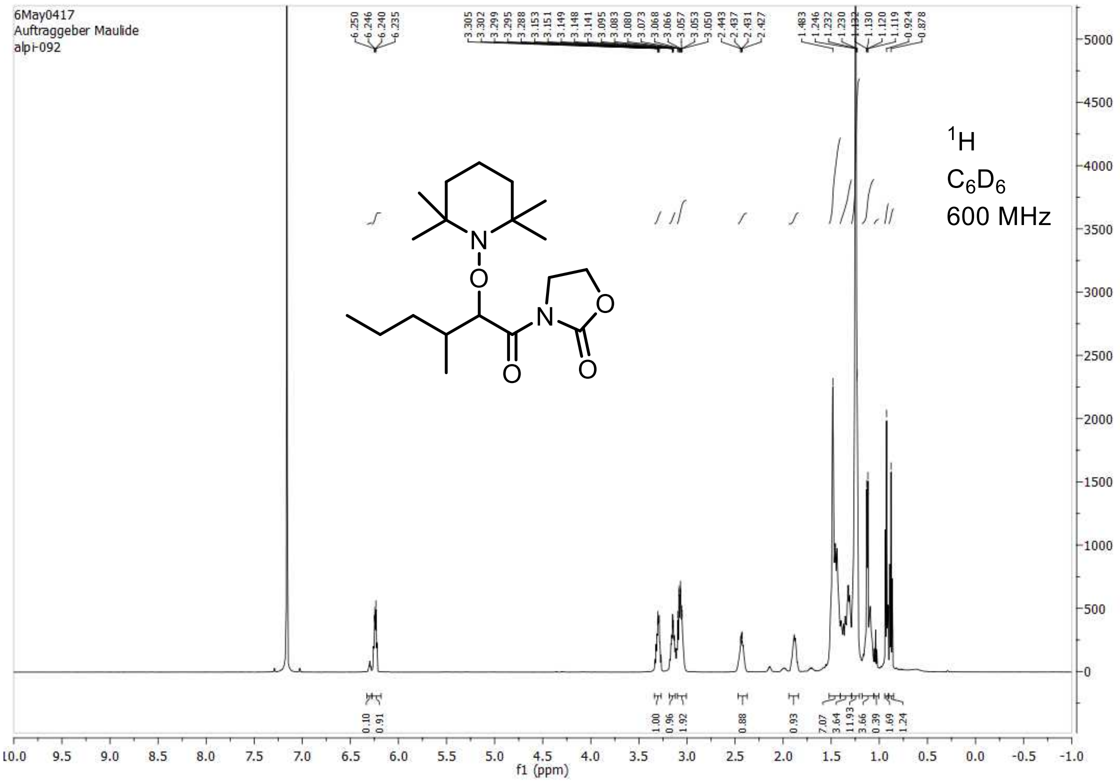
6May0417
Auftraggeber Maulide
alpi-092
^{1}H
C_6D_6
600 MHz
f1 (ppm)

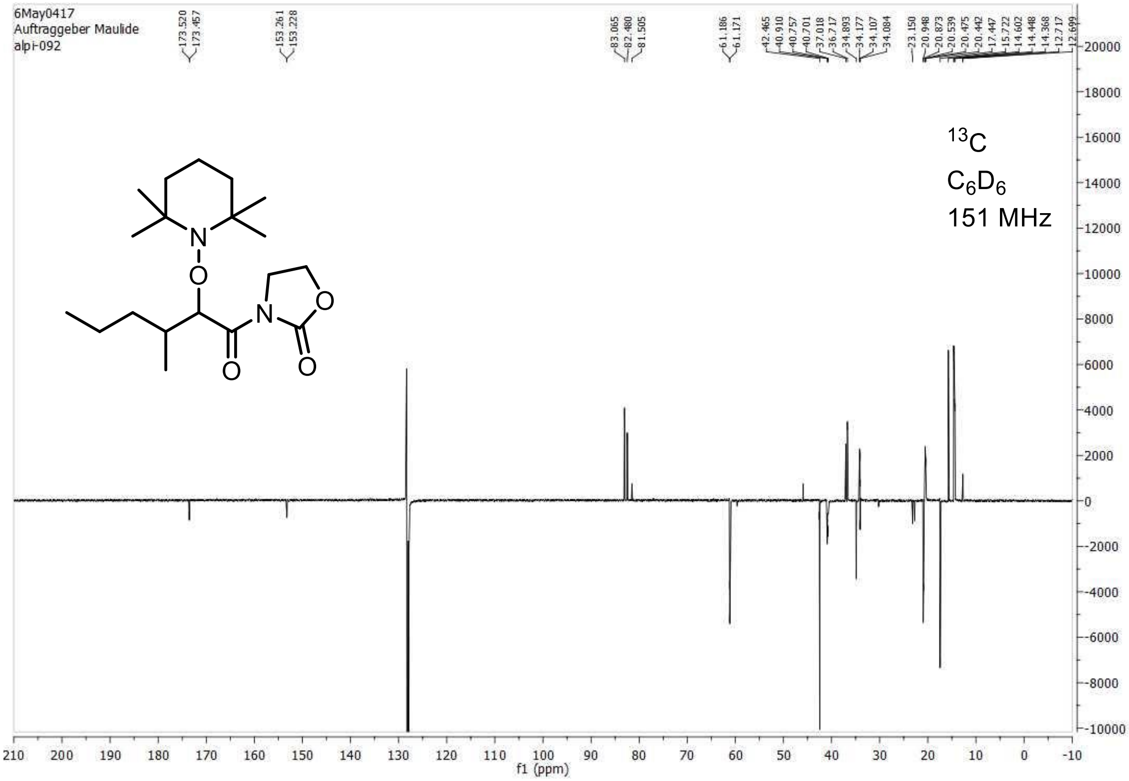
6May0417
Auftraggeber Maulide
alpi-092
^{13}C
C_6D_6
151 MHz
f1 (ppm)

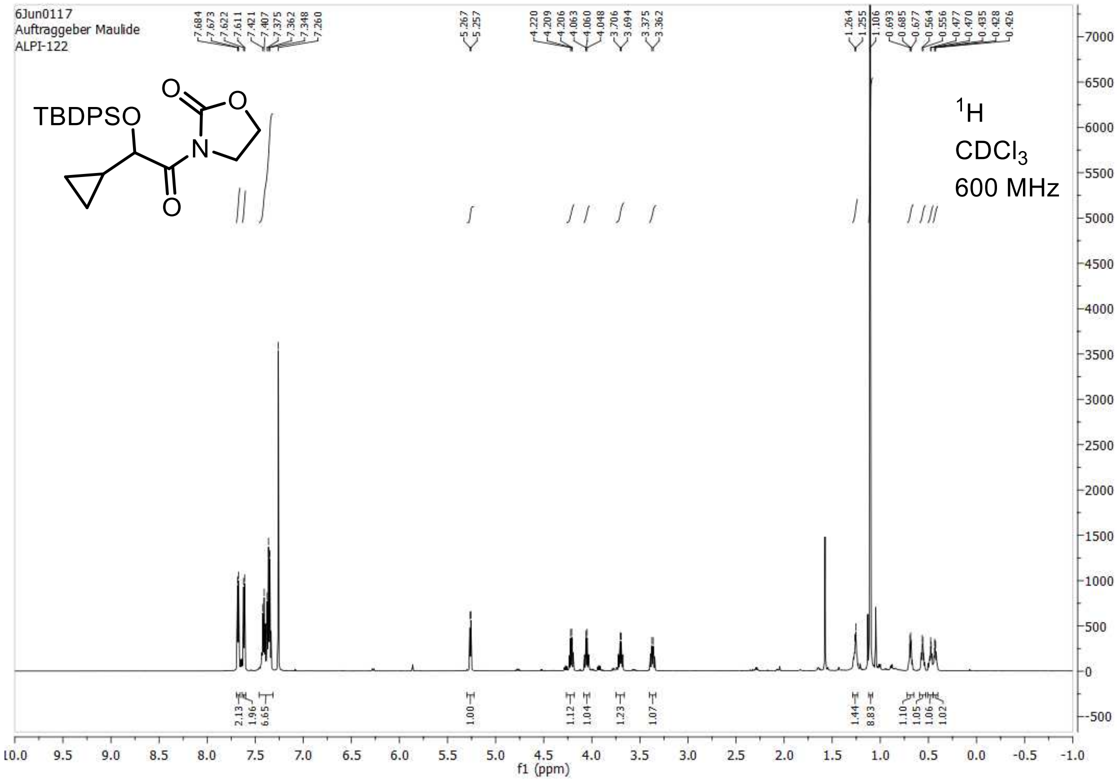

6Jun0117
Auftraggeber Maulide
ALPI-122
7.684
7.673
7.622
7.611
7.421
7.407
7.375
7.362
7.348
7.260
5.267
5.257
4.220
4.209
4.206
4.063
4.060
4.048
3.706
3.694
3.375
3.362
1.264
1.255
1.106
0.693
0.685
0.677
0.564
0.556
0.477
0.470
0.435
0.428
0.426
TBDPSO
O
O
N
O
O
1H
CDCl3
600 MHz
2.13
1.96
6.65
1.00
1.12
1.04
1.23
1.07
1.44
8.83
1.10
1.05
1.06
1.02
f1 (ppm)

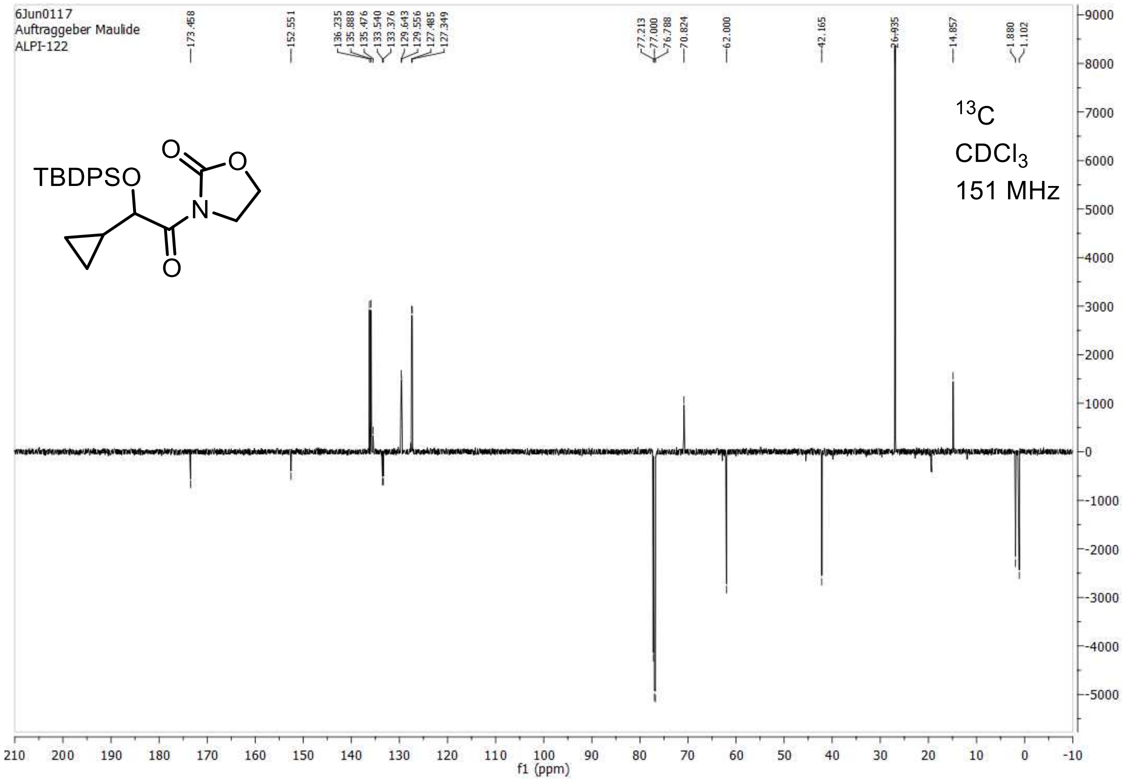

6Jun0117
Auftraggeber Maulide
ALPI-122
173.458
152.551
136.235
135.888
135.476
133.540
133.376
129.643
129.556
127.485
127.349
77.213
77.000
76.788
70.824
62.000
42.165
26.935
14.857
1.880
1.102
TBDPSO
O
O
N
O
O
13C
CDCl3
151 MHz
f1 (ppm)

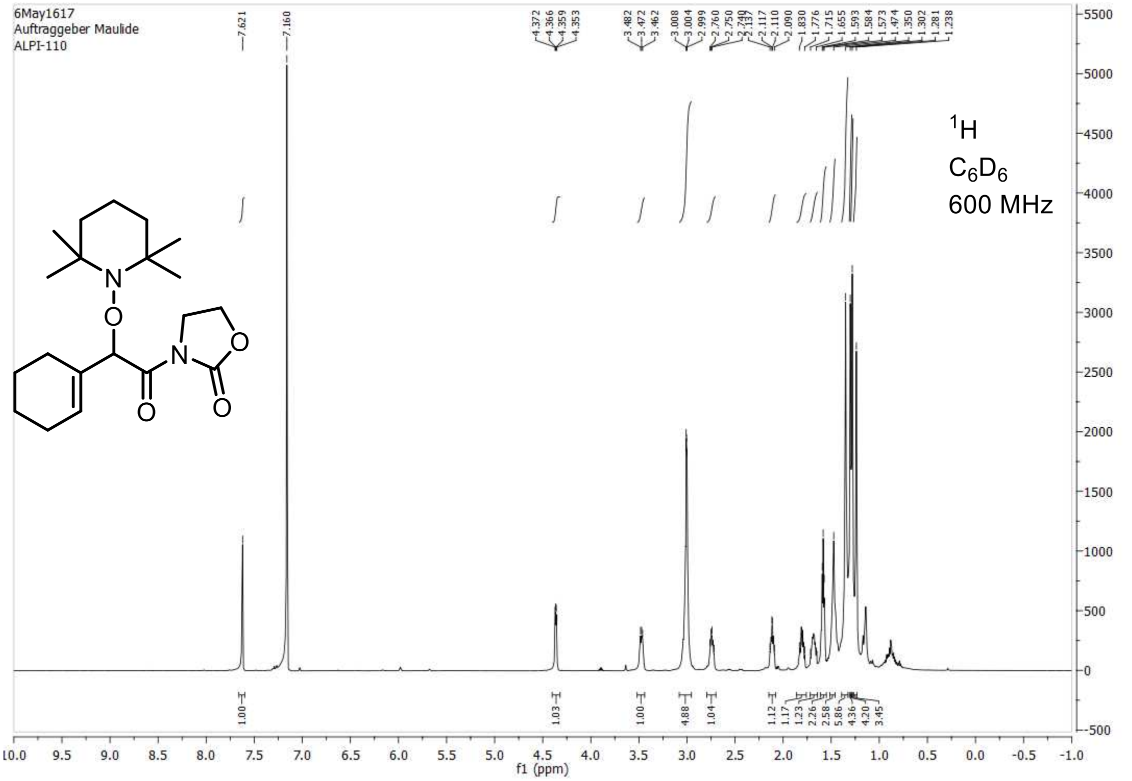
6May1617
Auftraggeber Maulide
ALPI-110
1H
C6D6
600 MHz
f1 (ppm)

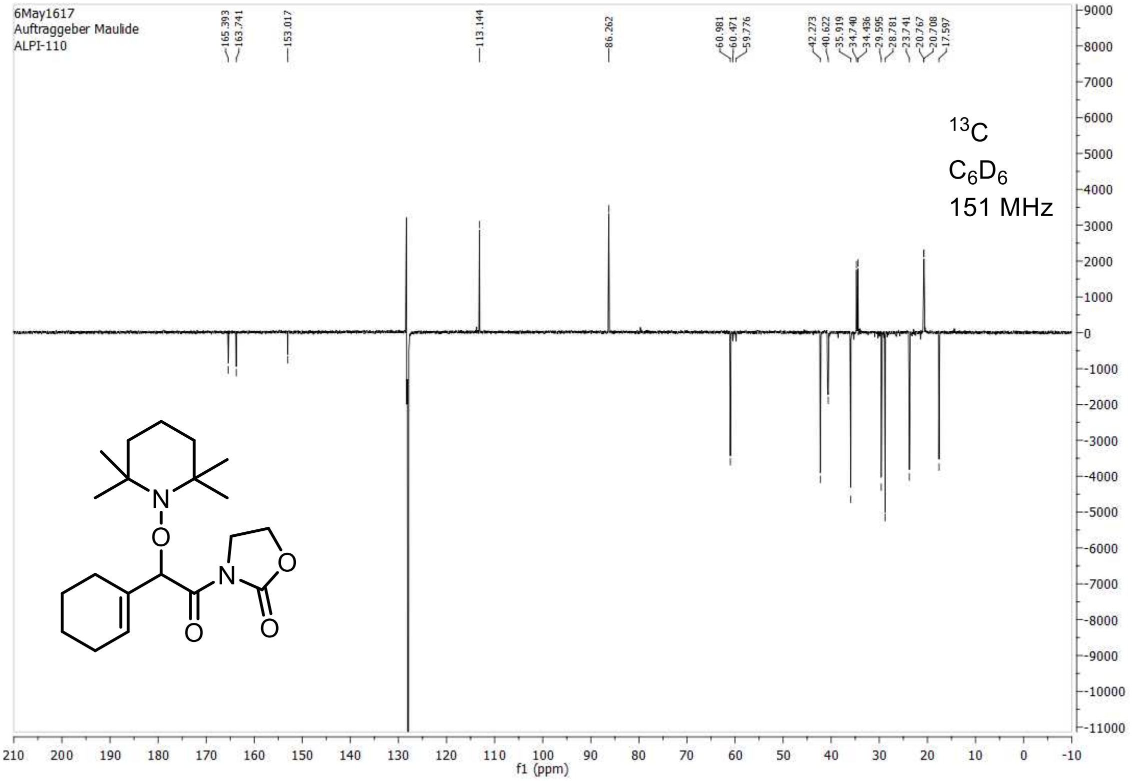
6May1617
Auftraggeber Maulide
ALPI-110
13C
C6D6
151 MHz
f1 (ppm)

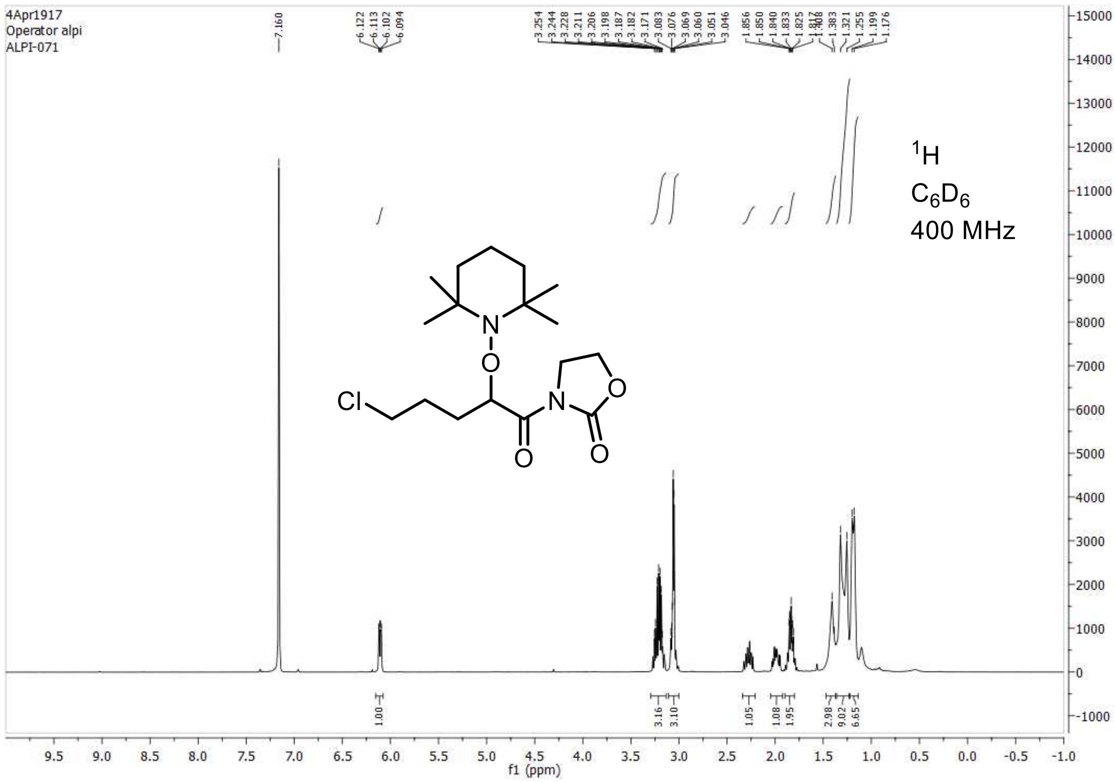
4Apr1917
Operator alpi
ALPI-071
^{1}H
C_6D_6
400 MHz
Cl
N
O
f1 (ppm)

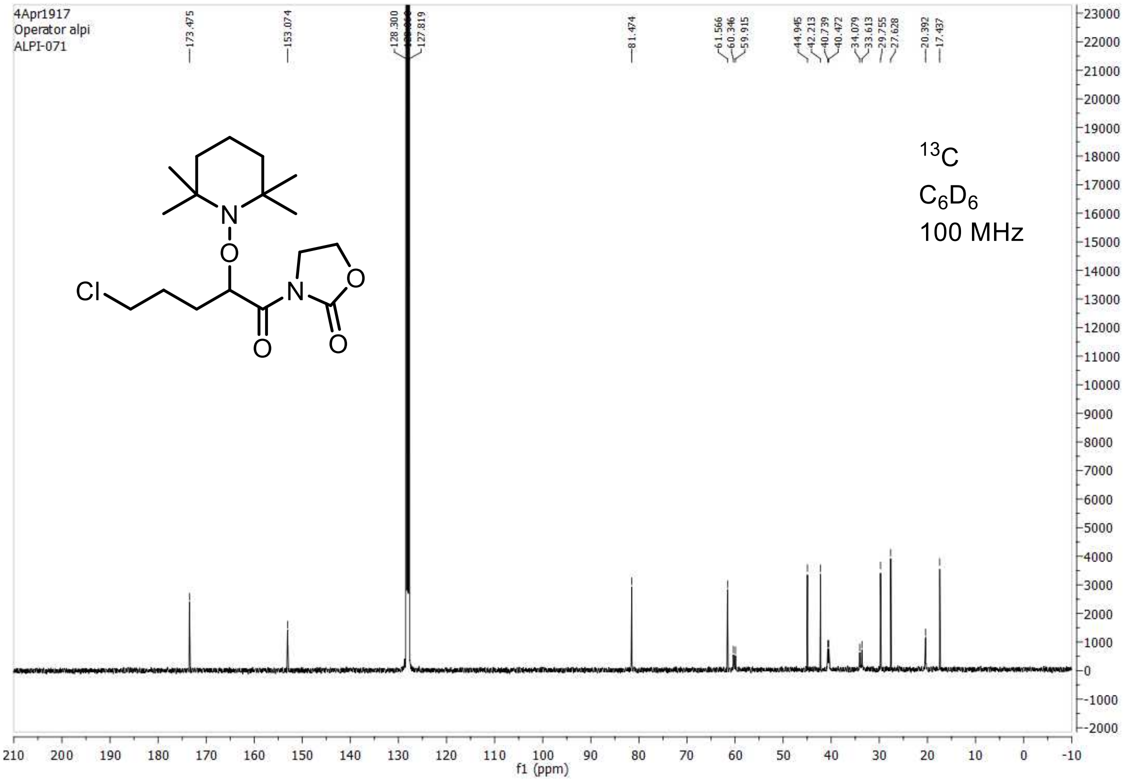
4Apr1917
Operator alpi
ALPI-071
^{13}C
C_6D_6
100 MHz
Cl
N
O
f1 (ppm)

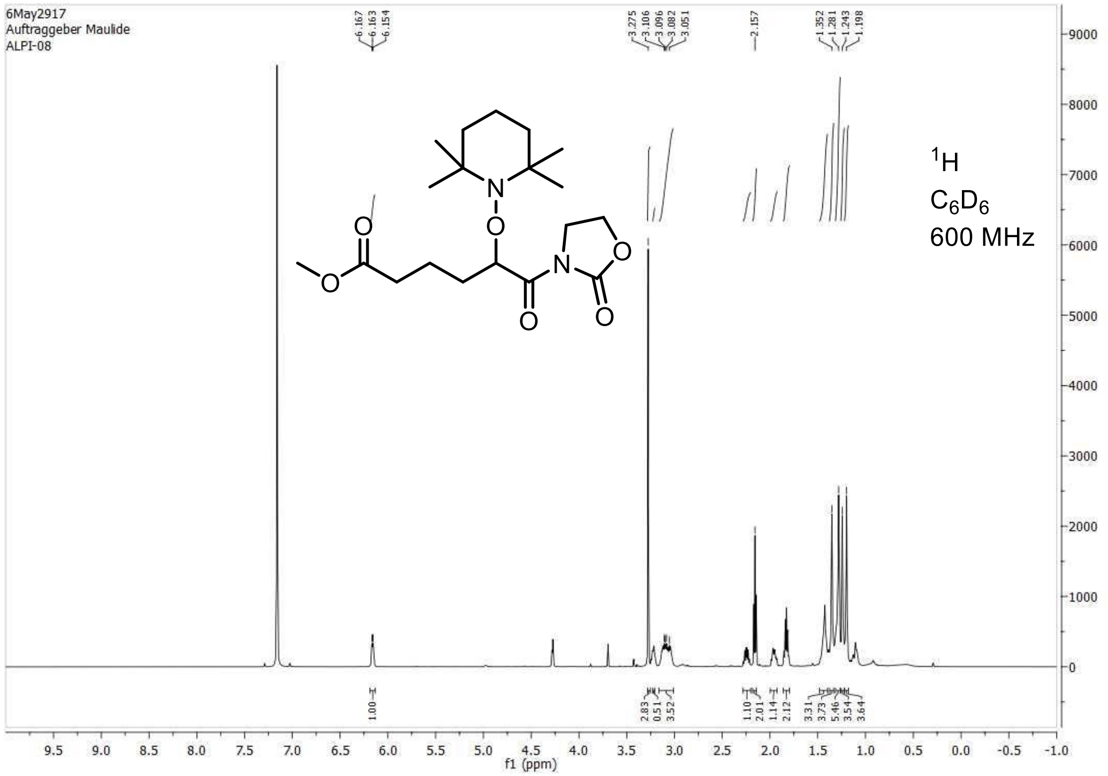

6May2917
Auftraggeber Maulide
ALPI-08
6.167
6.163
6.154
3.275
3.106
3.096
3.082
3.051
2.157
1.352
1.281
1.243
1.198
N
O
O
O
N
O
O
O
O
^{1}H
C_6D_6
600 MHz
1.00
2.83
0.51
3.52
1.10
2.01
1.14
2.12
3.31
3.73
5.46
3.54
3.64
f1 (ppm)

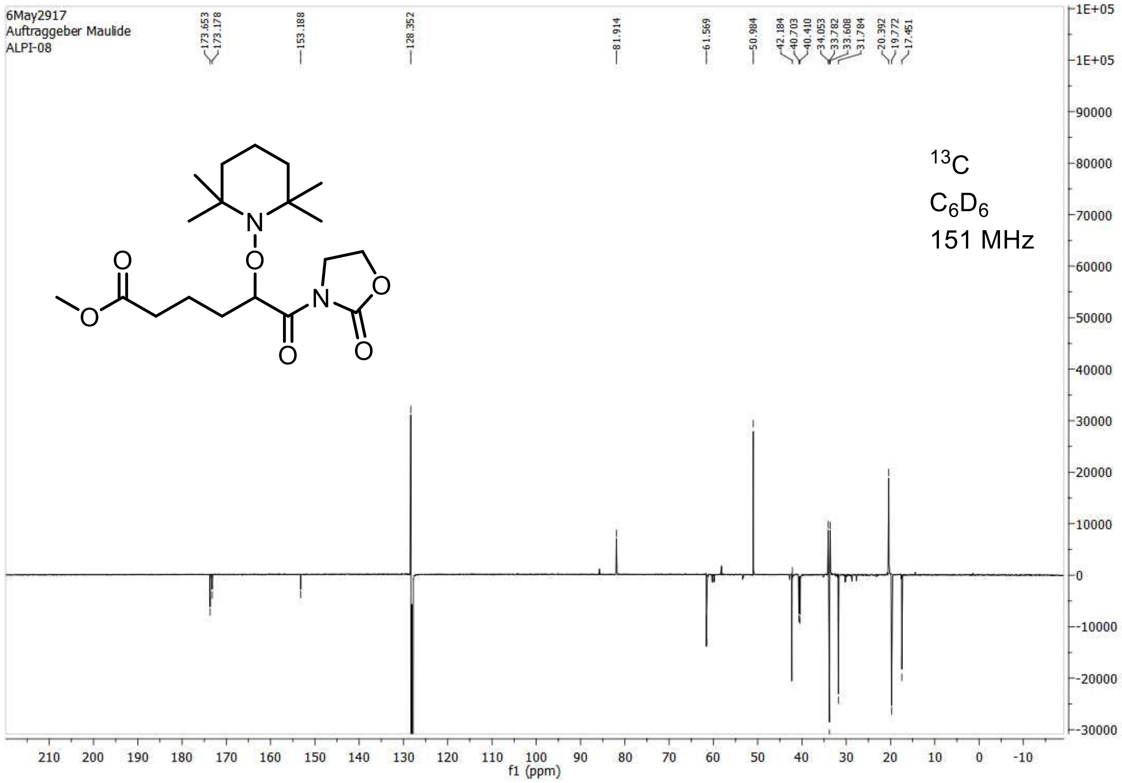

6May2917
Auftraggeber Maulide
ALPI-08
173.653
173.178
153.188
128.352
81.914
61.569
50.984
42.184
40.703
40.410
34.053
33.782
33.608
31.784
20.392
19.772
17.451
N
O
O
O
N
O
O
O
O
^{13}C
C_6D_6
151 MHz
f1 (ppm)

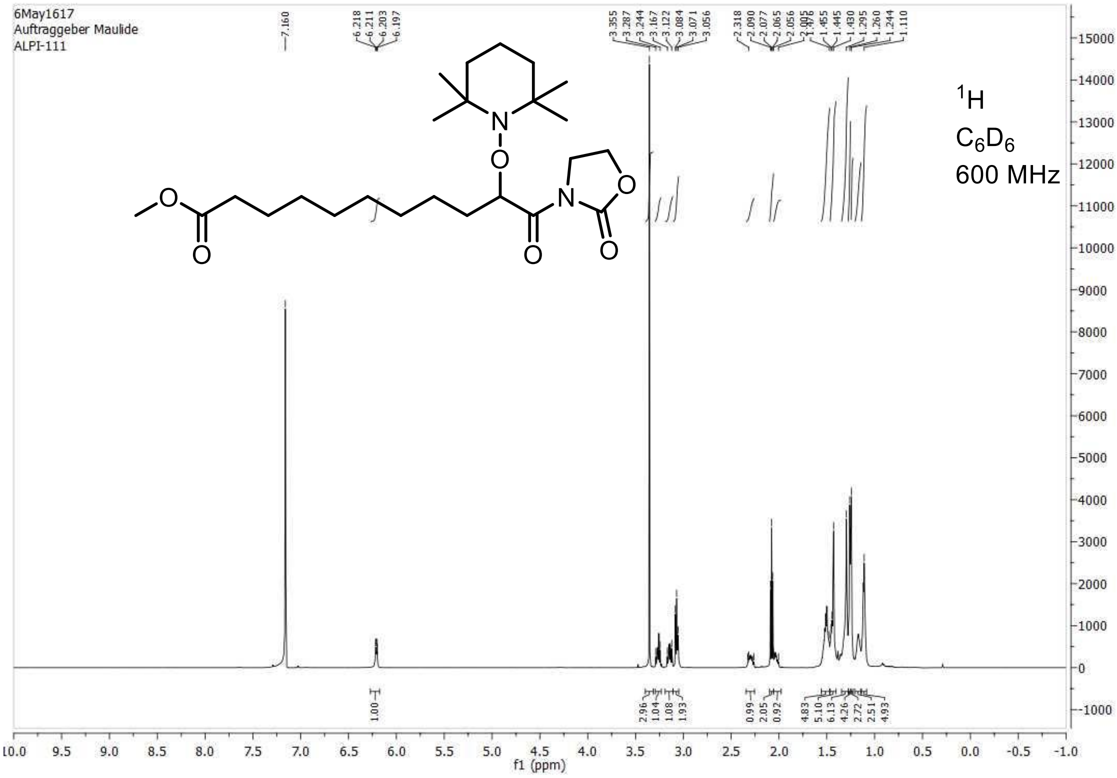
6May1617
Auftraggeber Maulide
ALPI-111
^1H
C_6D_6
600 MHz
f1 (ppm)

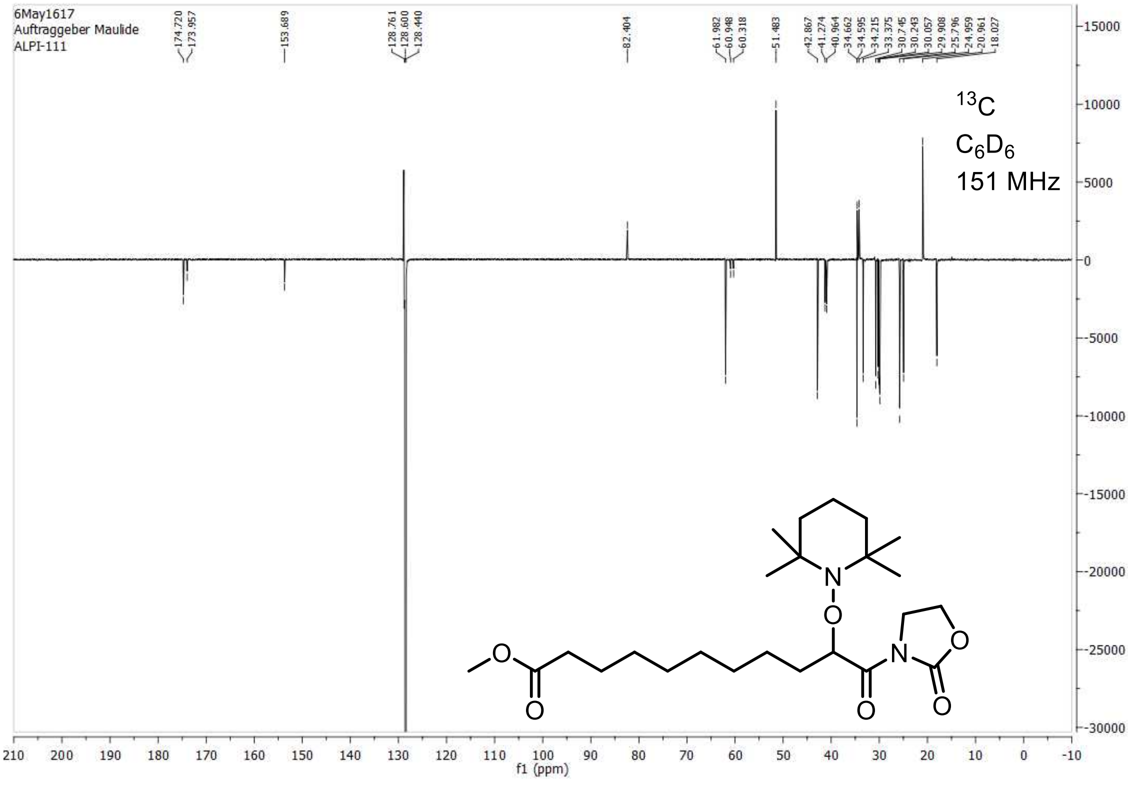
6May1617
Auftraggeber Maulide
ALPI-111
^{13}C
C_6D_6
151 MHz
f1 (ppm)

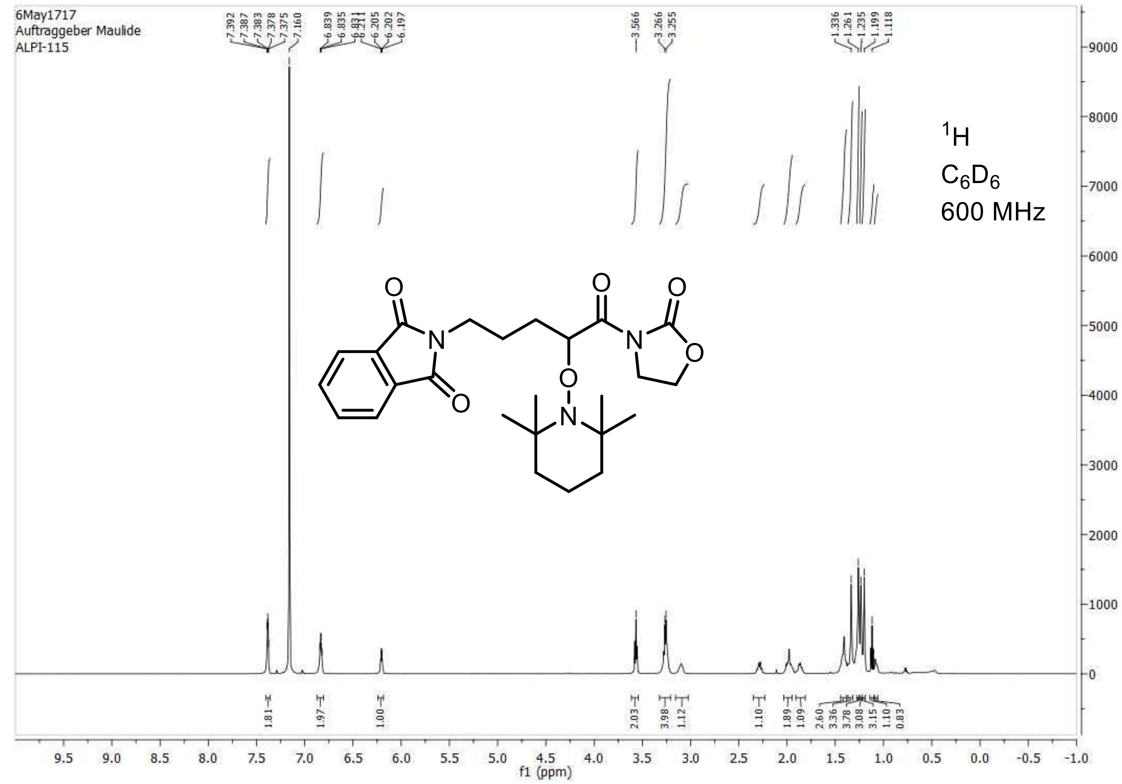
6May1717
Auftraggeber Maulide
ALPI-115
1H
C6D6
600 MHz
f1 (ppm)

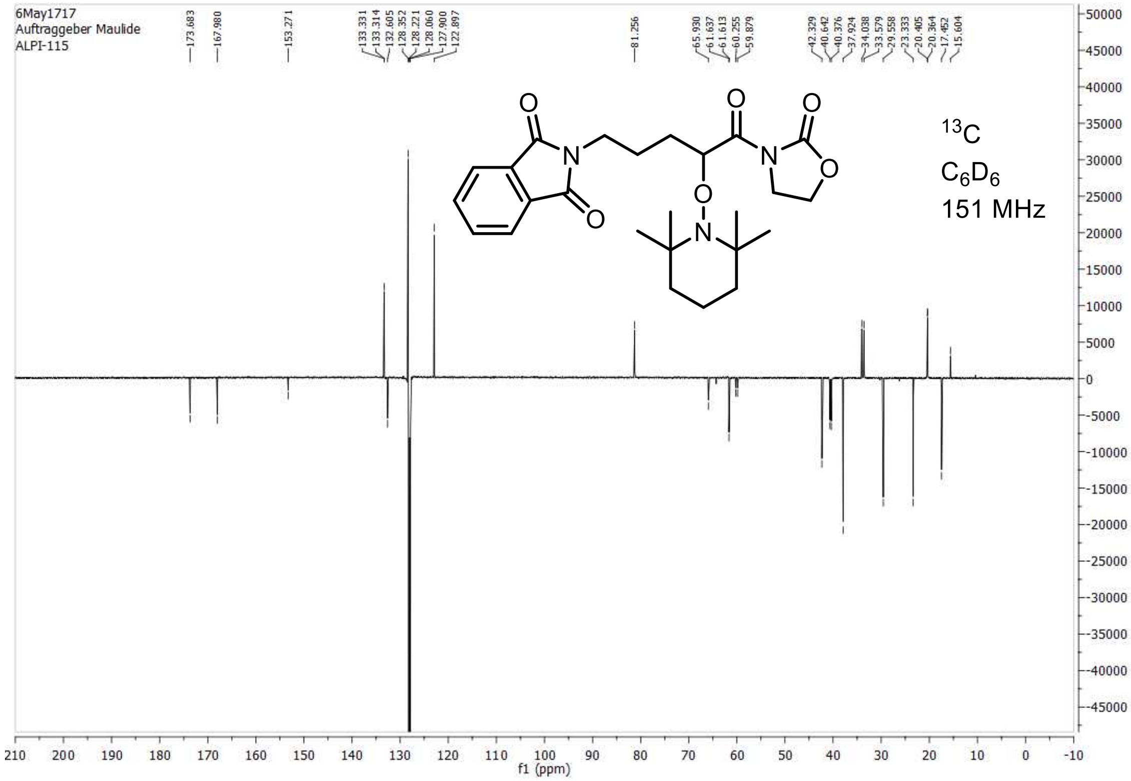
6May1717
Auftraggeber Maulide
ALPI-115
13C
C6D6
151 MHz
f1 (ppm)

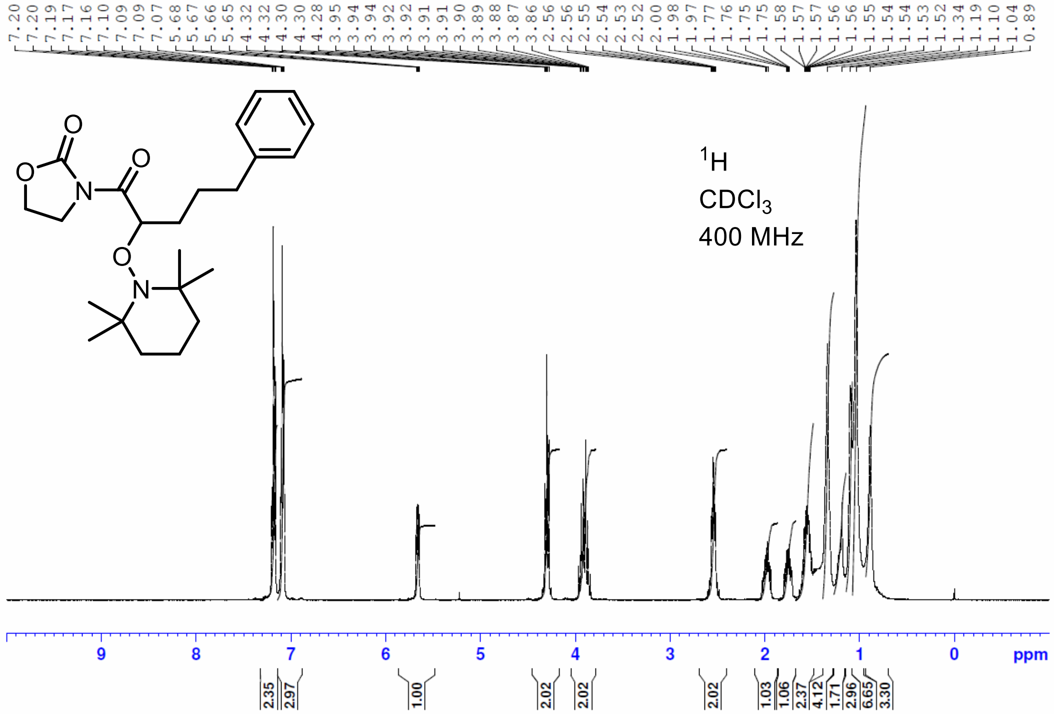

7.20
7.20
7.19
7.17
7.16
7.10
7.09
7.09
7.07
5.68
5.67
5.66
5.65
4.32
4.30
4.30
4.28
3.95
3.94
3.94
3.92
3.92
3.91
3.91
3.90
3.89
3.88
3.87
3.86
2.56
2.56
2.55
2.54
2.53
2.52
2.00
1.98
1.97
1.77
1.76
1.75
1.75
1.58
1.57
1.57
1.56
1.56
1.55
1.54
1.54
1.53
1.52
1.34
1.19
1.10
1.04
0.89
O
O
O
N
O
N
^{1}H
$CDCl_3$
400 MHz
9
8
7
6
5
4
3
2
1
0
ppm
2.35
2.97
1.00
2.02
2.02
2.02
1.03
1.06
2.37
4.12
1.71
2.96
6.65
3.30

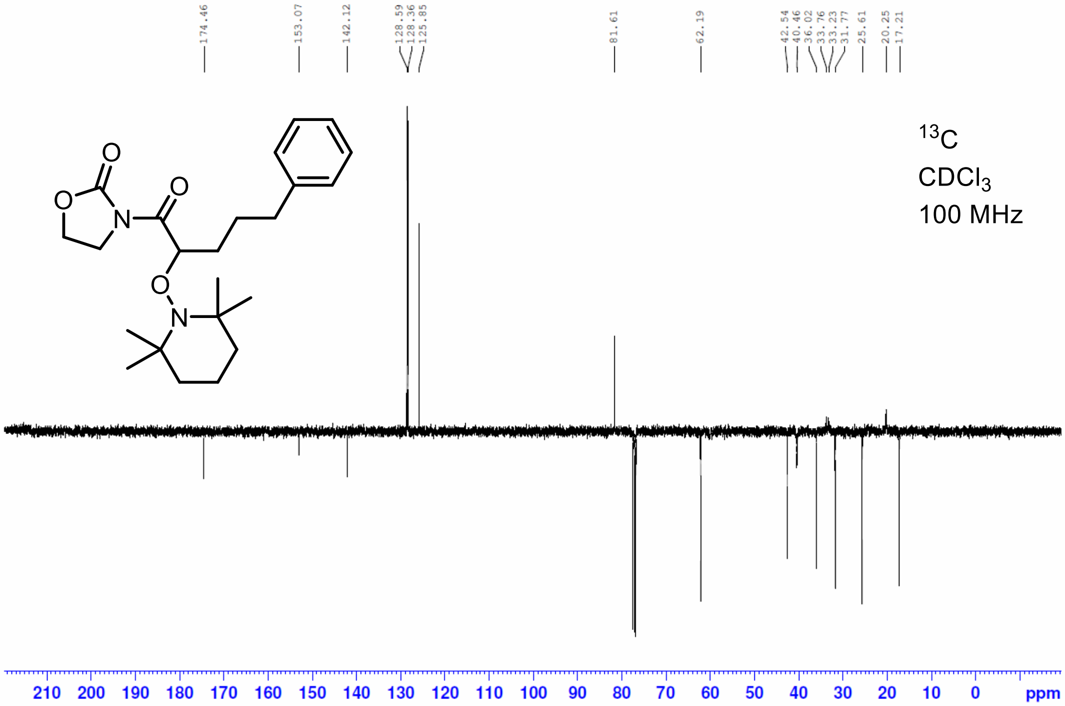

174.46
153.07
142.12
128.59
128.36
125.85
81.61
62.19
42.54
40.46
36.02
33.76
33.23
31.77
25.61
20.25
17.21
O
O
O
N
O
N
^{13}C
$CDCl_3$
100 MHz
210
200
190
180
170
160
150
140
130
120
110
100
90
80
70
60
50
40
30
20
10
0
ppm

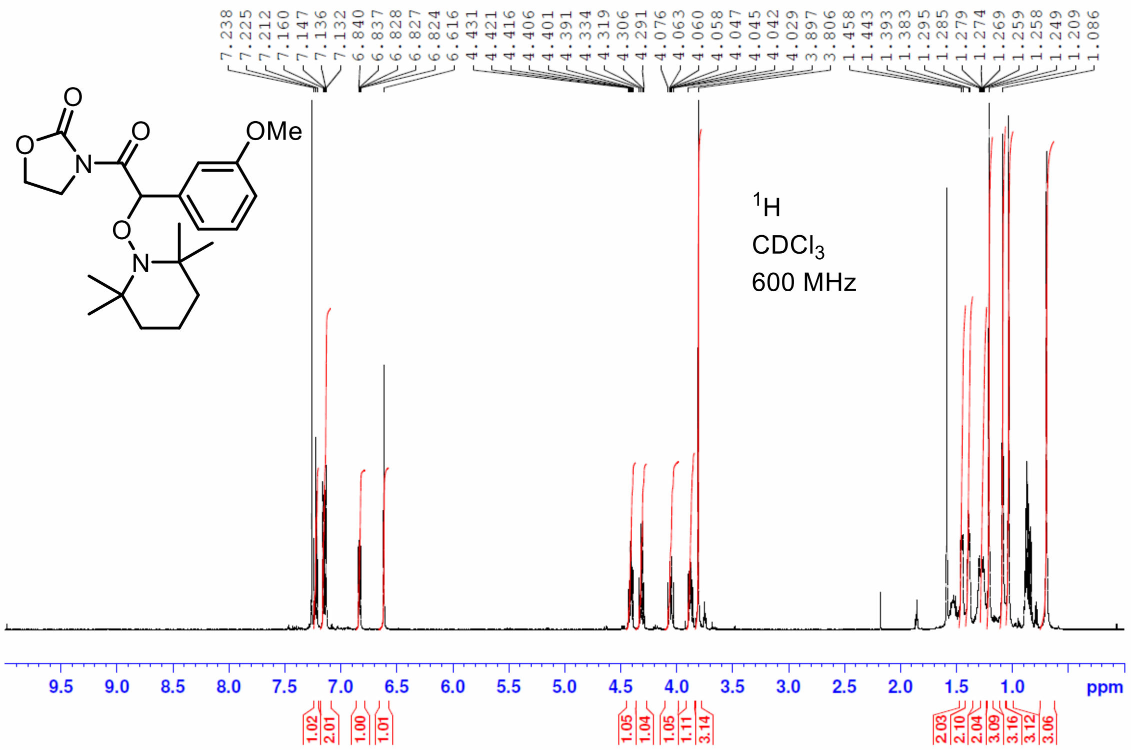

7.238
7.225
7.212
7.160
7.147
7.136
7.132
6.840
6.837
6.828
6.827
6.824
6.616
4.431
4.421
4.416
4.406
4.401
4.391
4.334
4.319
4.306
4.291
4.076
4.063
4.060
4.058
4.047
4.045
4.042
4.029
3.897
3.806
1.458
1.443
1.393
1.383
1.295
1.285
1.279
1.274
1.269
1.259
1.258
1.249
1.209
1.086
OMe
^{1}H
$CDCl_3$
600 MHz
ppm

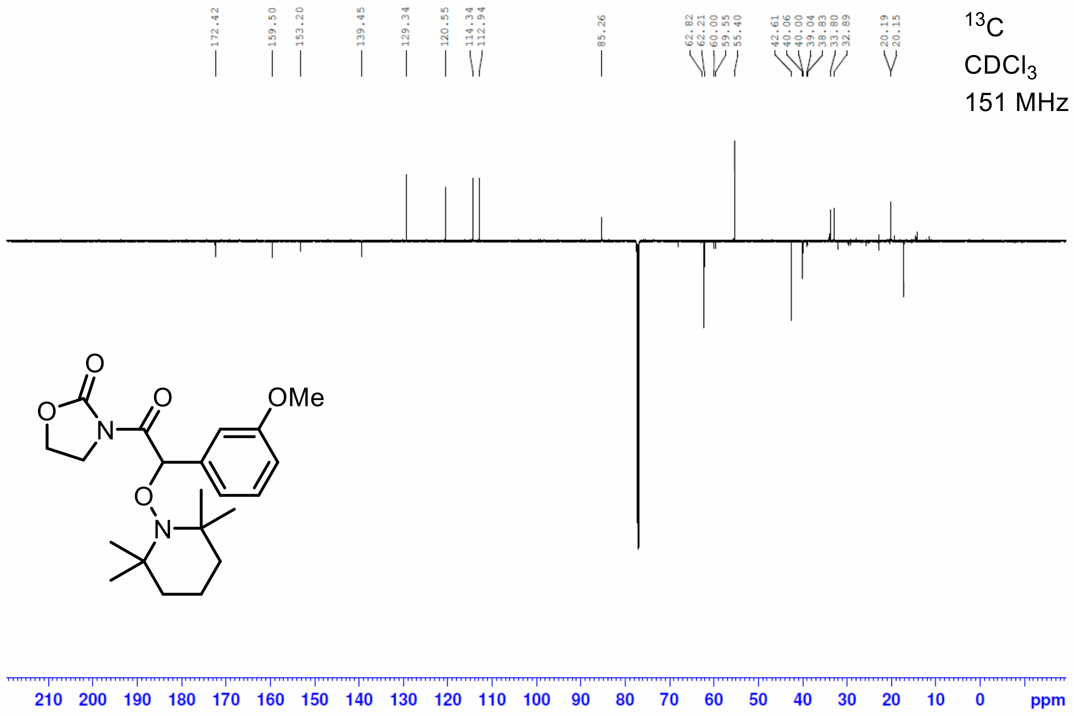

172.42
159.50
153.20
139.45
129.34
120.55
114.34
112.94
85.26
62.82
62.21
60.00
59.55
55.40
42.61
40.06
40.00
39.04
38.83
33.80
32.89
20.19
20.15
^{13}C
$CDCl_3$
151 MHz
OMe
ppm

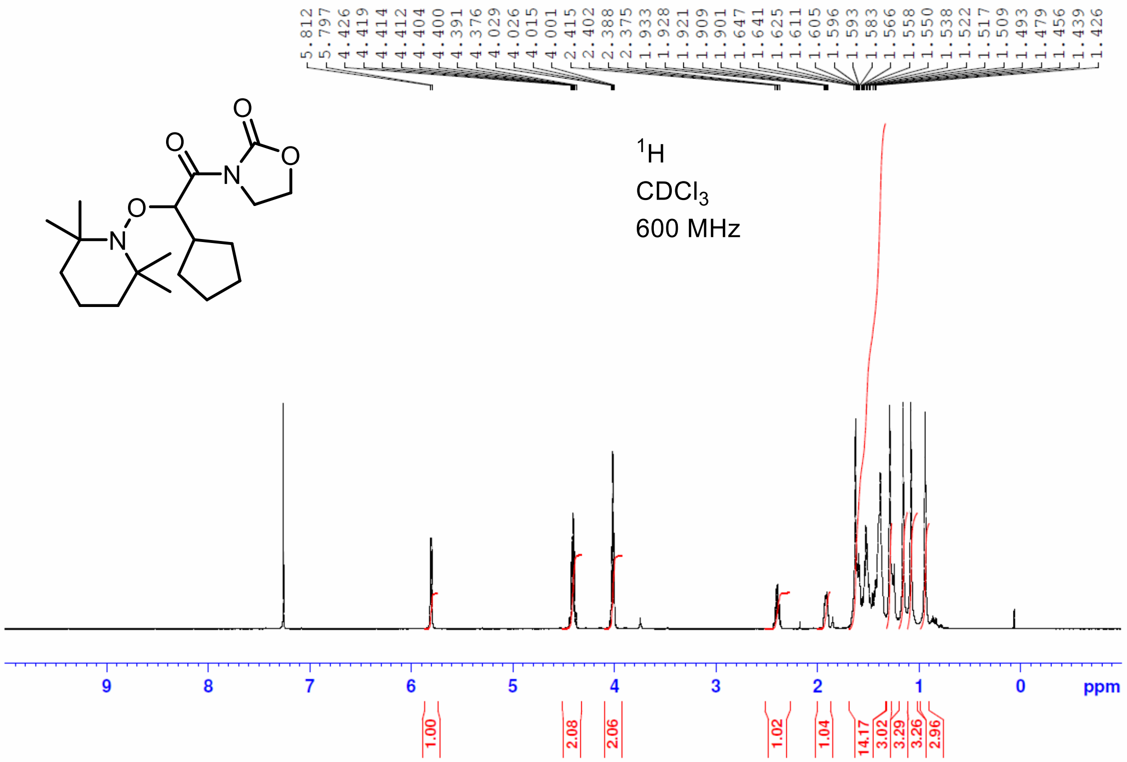
5.812
5.797
4.426
4.419
4.414
4.412
4.404
4.400
4.391
4.376
4.029
4.026
4.015
4.001
2.415
2.402
2.388
2.375
1.933
1.928
1.921
1.909
1.901
1.647
1.641
1.625
1.611
1.605
1.596
1.593
1.583
1.566
1.558
1.550
1.538
1.522
1.517
1.509
1.493
1.479
1.456
1.439
1.426
O
O
N
O
O
N
^{1}H
$CDCl_3$
600 MHz
9 8 7 6 5 4 3 2 1 0 ppm
1.00
2.08
2.06
1.02
1.04
14.17
3.02
3.29
3.26
2.96

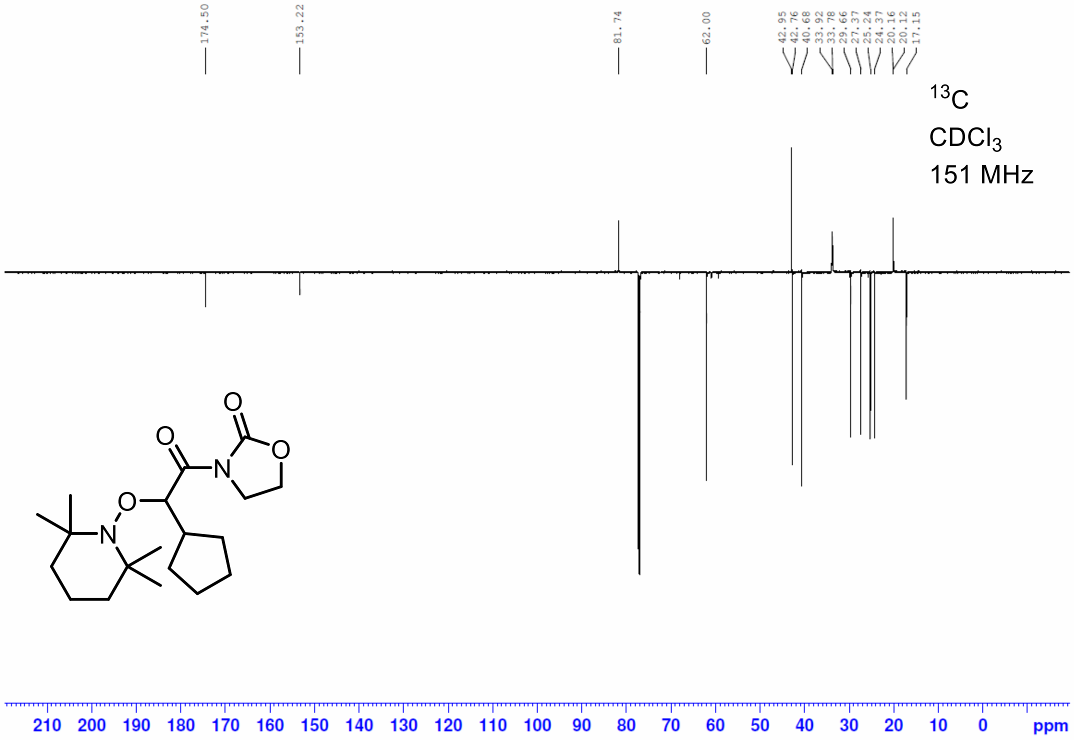
174.50
153.22
81.74
62.00
42.95
42.76
40.68
33.92
33.78
29.66
27.37
25.24
24.37
20.16
20.12
17.15
^{13}C
$CDCl_3$
151 MHz
O
O
N
O
O
N
210 200 190 180 170 160 150 140 130 120 110 100 90 80 70 60 50 40 30 20 10 0 ppm

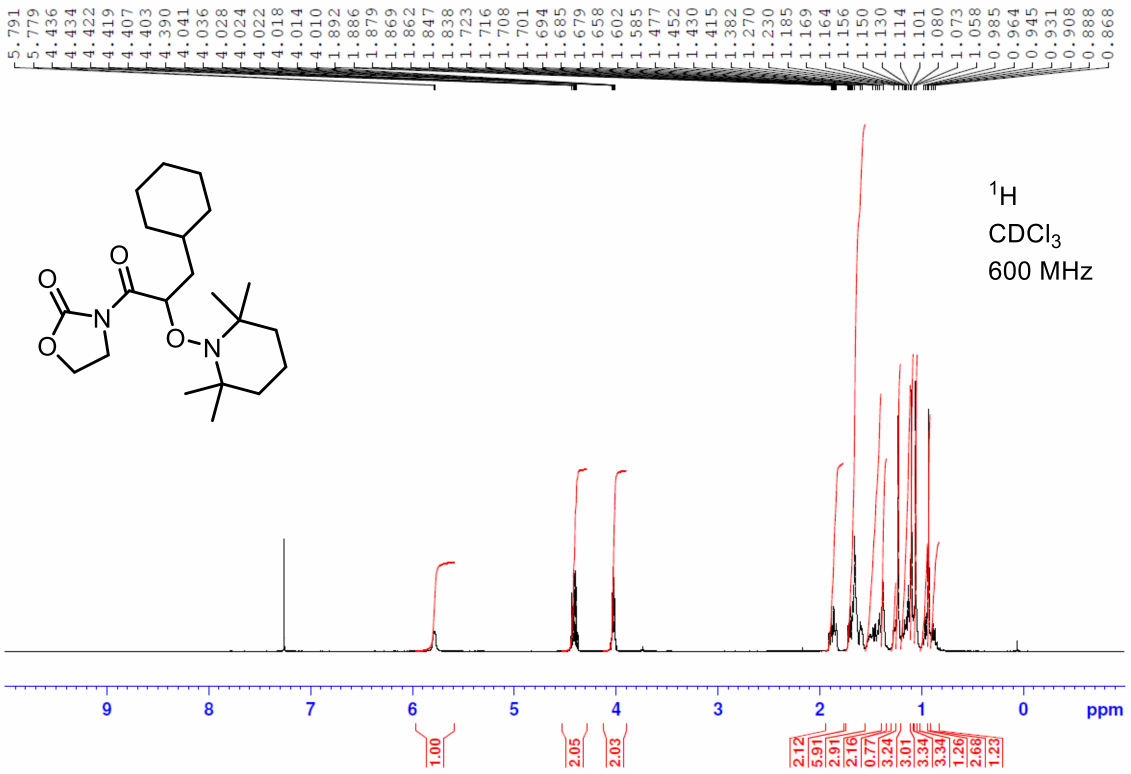

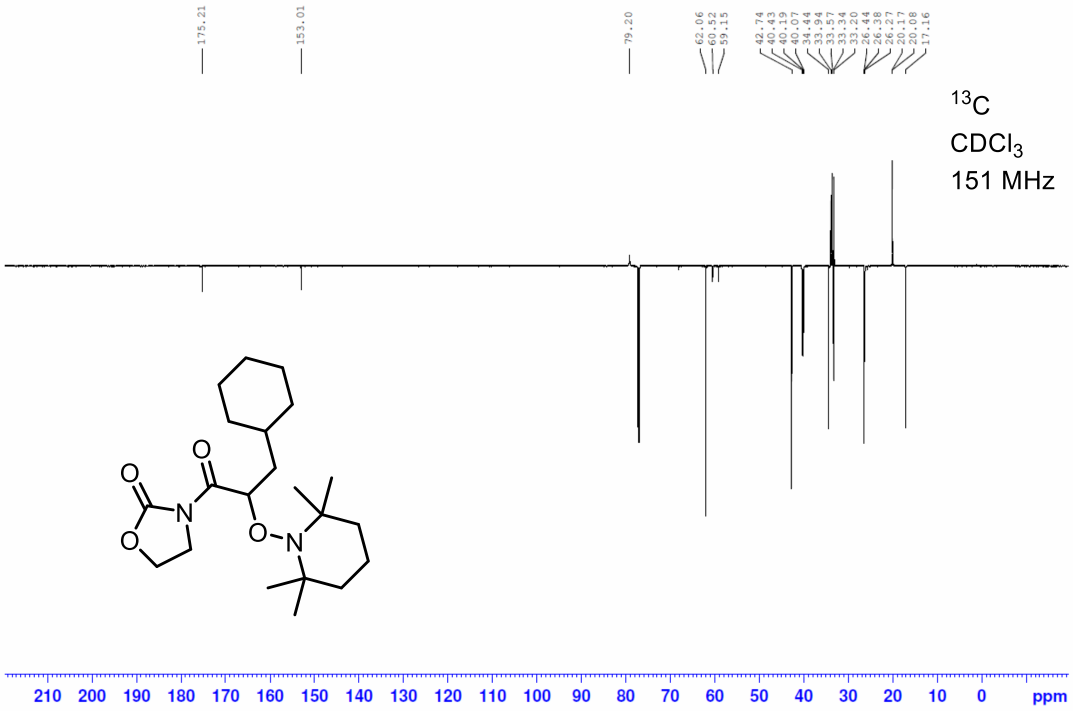

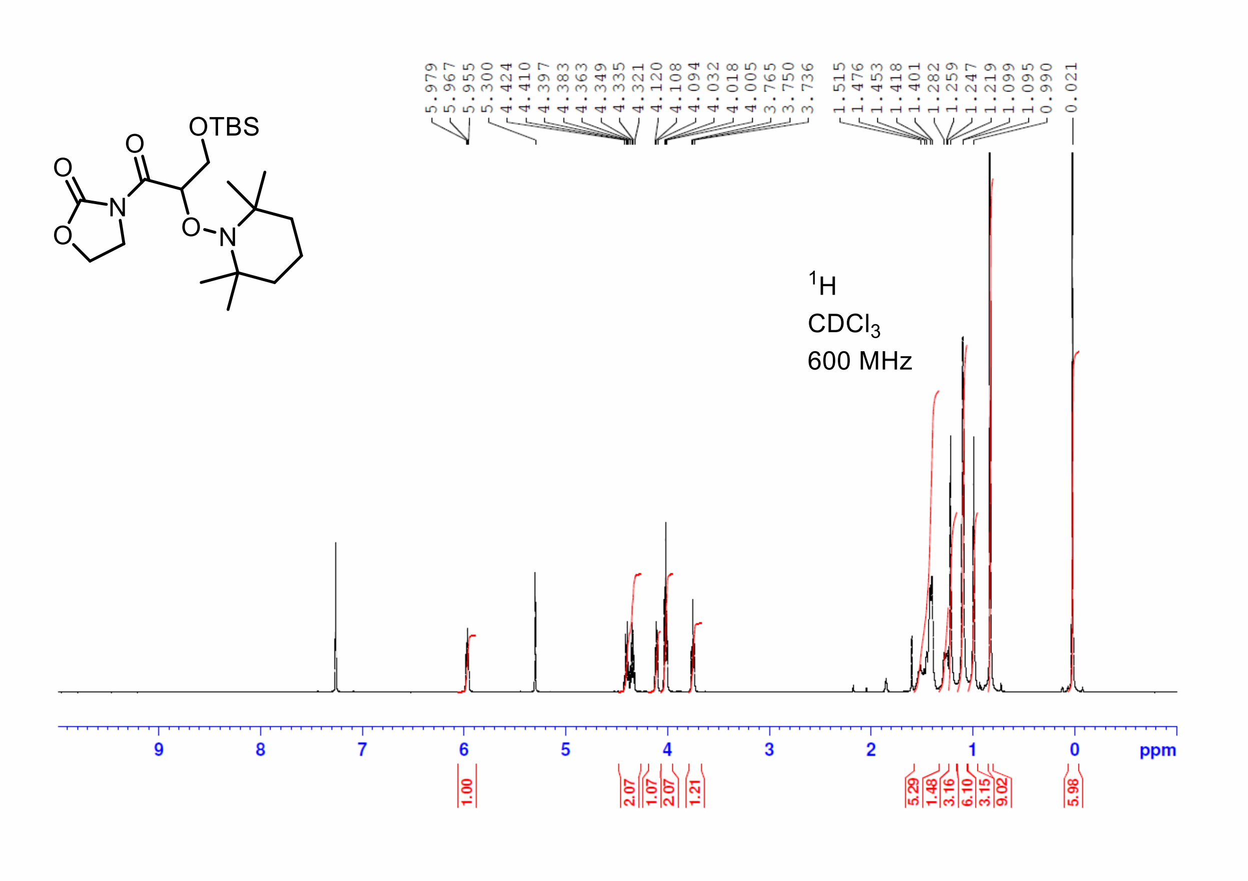
OTBS
O
O
N
O
O
N
1H
CDCl3
600 MHz
5.979
5.967
5.955
5.300
4.424
4.410
4.397
4.383
4.363
4.349
4.335
4.321
4.120
4.108
4.094
4.032
4.018
4.005
3.765
3.750
3.736
1.515
1.476
1.453
1.418
1.401
1.282
1.259
1.247
1.219
1.099
1.095
0.990
0.021
9
8
7
6
5
4
3
2
1
0
ppm
1.00
2.07
1.07
2.07
1.21
5.29
1.48
3.16
6.10
3.15
9.02
5.98

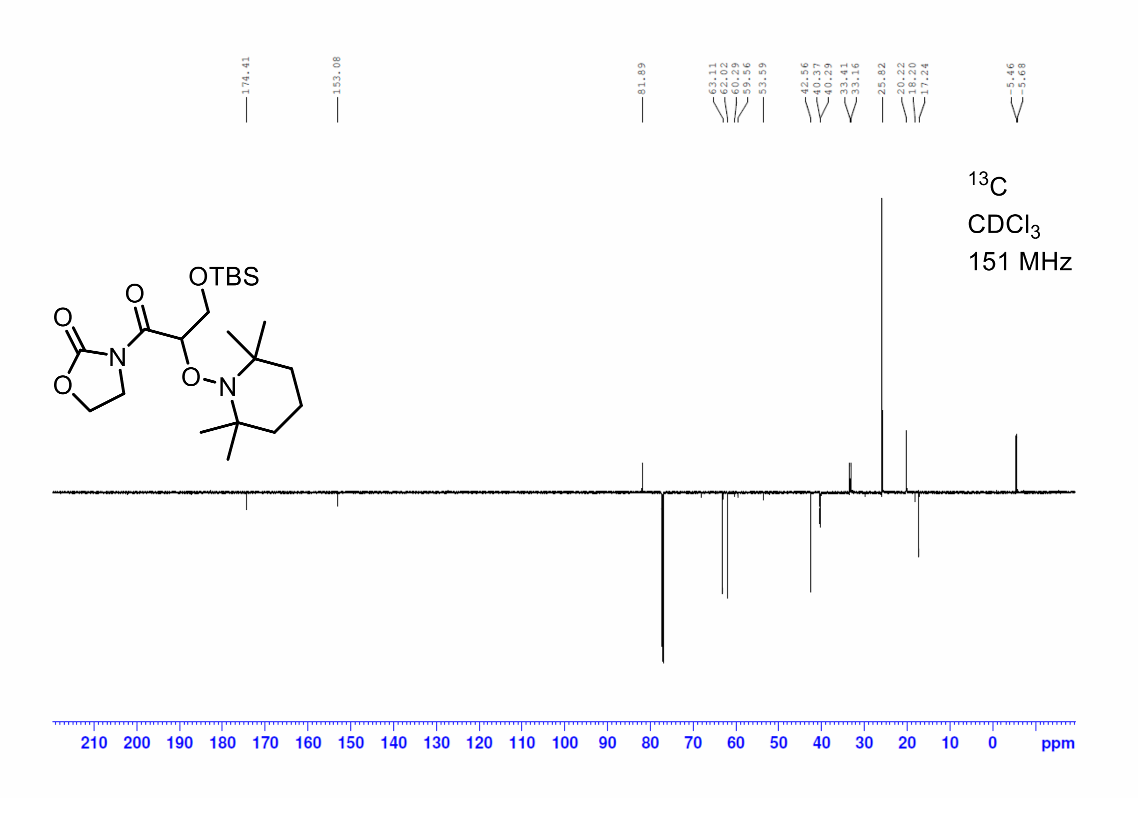
174.41
153.08
81.89
63.11
62.02
60.29
59.56
53.59
42.56
40.37
40.29
33.41
33.16
25.82
20.22
18.20
17.24
-5.46
-5.68
13C
CDCl3
151 MHz
OTBS
O
O
N
O
O
N
210
200
190
180
170
160
150
140
130
120
110
100
90
80
70
60
50
40
30
20
10
0
ppm

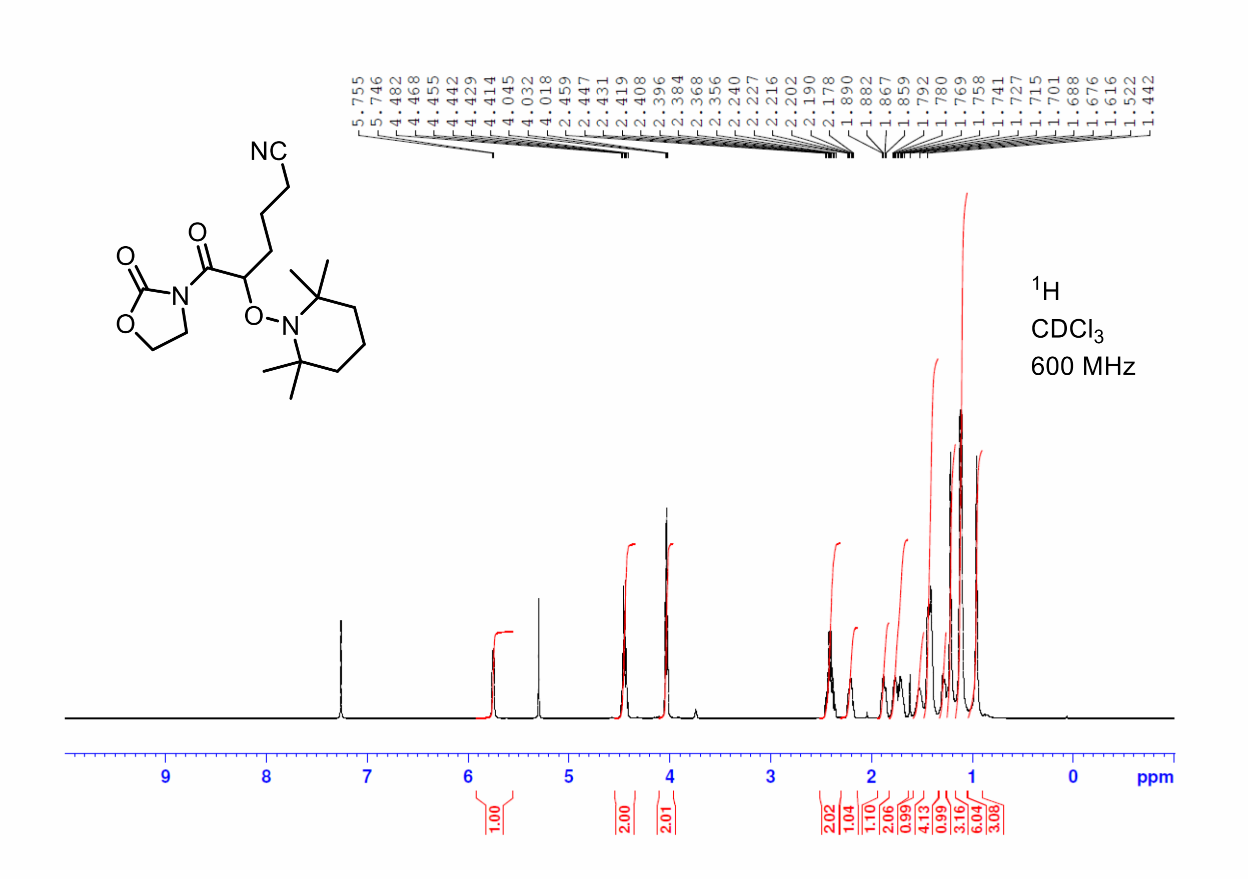
5.755 5.746 4.482 4.468 4.455 4.442 4.429 4.414 4.045 4.032 4.018 2.459 2.447 2.431 2.419 2.408 2.396 2.384 2.368 2.356 2.240 2.227 2.216 2.202 2.190 2.178 1.890 1.882 1.867 1.859 1.792 1.780 1.769 1.758 1.741 1.727 1.715 1.701 1.688 1.676 1.616 1.522 1.442
NC
O
O
N
O
O
N
1H
CDCl3
600 MHz
9 8 7 6 5 4 3 2 1 0 ppm
1.00 2.00 2.01 2.02 1.04 1.10 2.06 0.99 4.13 0.99 3.16 6.04 3.08

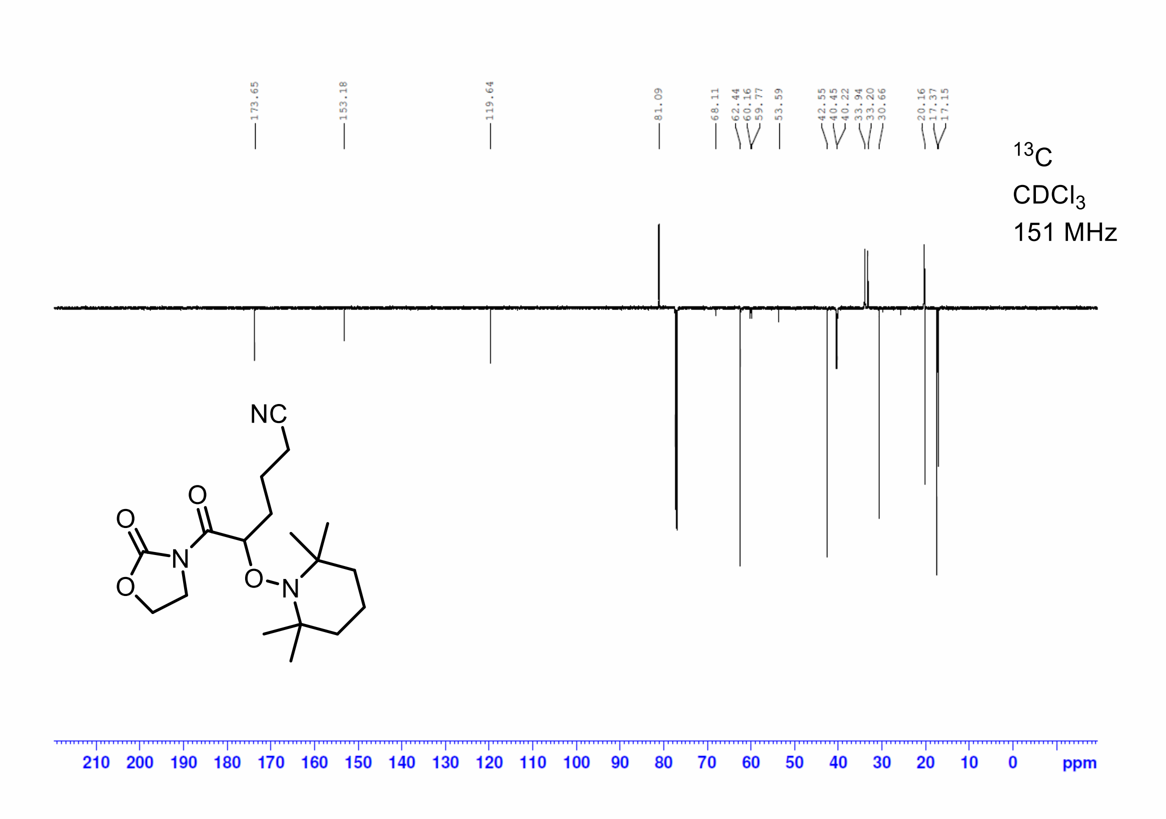
173.65 153.18 119.64 81.09 68.11 62.44 60.16 59.77 53.59 42.55 40.45 40.22 33.94 33.20 30.66 20.16 17.37 17.15
13C
CDCl3
151 MHz
NC
O
O
N
O
O
N
210 200 190 180 170 160 150 140 130 120 110 100 90 80 70 60 50 40 30 20 10 0 ppm

6.3. Derivatives

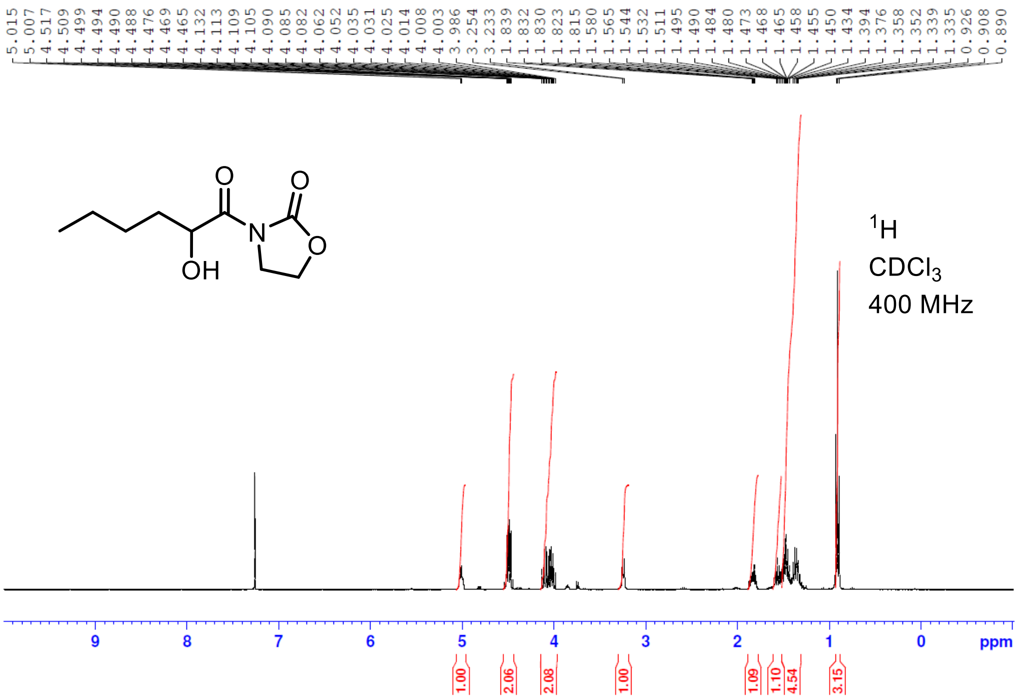

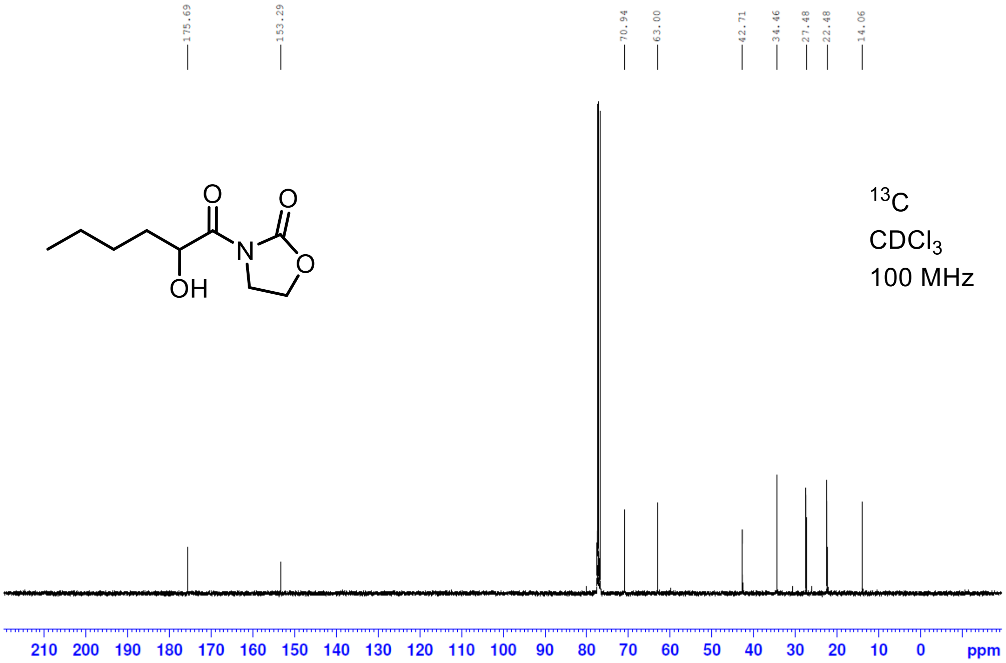

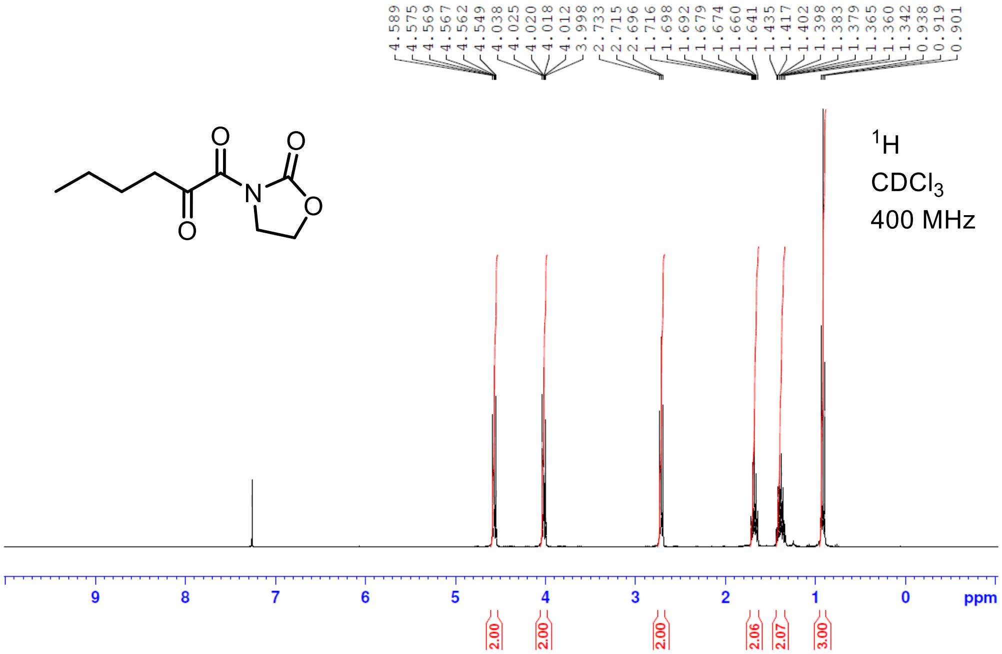
4.589
4.575
4.569
4.567
4.562
4.549
4.038
4.025
4.020
4.018
4.012
3.998
2.733
2.715
2.696
1.716
1.698
1.692
1.679
1.674
1.660
1.641
1.435
1.417
1.402
1.398
1.383
1.379
1.365
1.360
1.342
0.938
0.919
0.901
^{1}H
$CDCl_3$
400 MHz
9
8
7
6
5
4
3
2
1
0
ppm
2.00
2.00
2.00
2.06
2.07
3.00

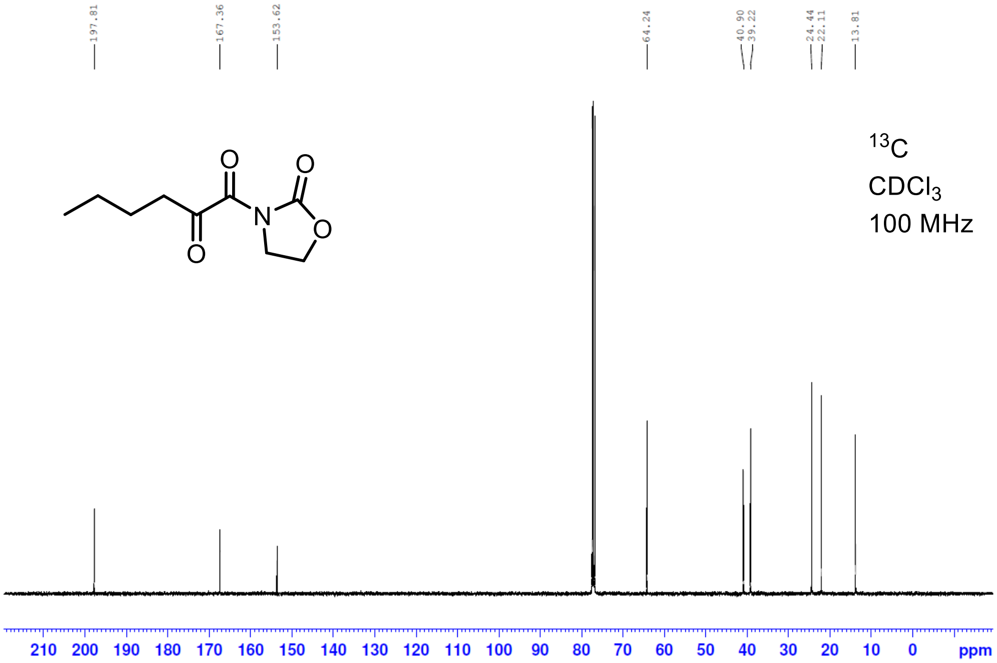
197.81
167.36
153.62
64.24
40.90
39.22
24.44
22.11
13.81
^{13}C
$CDCl_3$
100 MHz
210
200
190
180
170
160
150
140
130
120
110
100
90
80
70
60
50
40
30
20
10
0
ppm

7. DFT-Calculations

Computational details

The conformational space of all flexible molecules has been initially explored using OPLS_2005 force field[18] and the systematic search routine implemented in MACROMODEL 11.5.[19]

The structures found at force field level have then been reoptimized at the (U)PBE0-D3/6-31G(d) level of theory.[20] The nature of all stationary points (minima and transition states) was verified through computation of the vibrational frequencies. The thermal corrections to the Gibbs free energy were obtained at the same level of theory. All energies are reported in kcal mol^{-1}. The density-based solvation model SMD[21] was applied to consider solvent effects. All calculations have been performed with the Gaussian09 program package.[22]

The approximate spin-correction procedure proposed by Yamaguchi et al.[23] has been applied to compute spin-projected energies:

$$\Psi_{(UDFT)} = C_S\,{}^1\varphi + C_T\,{}^3\varphi$$

$${}^1E_{(SC)} = {}^1E_{(UDFT)} + f_{SC}[{}^1E_{(UDFT)} - {}^3E_{(UDFT)}]$$

$$f_{SC} = C_T^2/1-C_S^2 = {}^1\langle S^2\rangle\,({}^3\langle S^2\rangle - {}^1\langle S^2\rangle).$$

This approach was successfully applied by *e.g*. Bendikov for Diels-Alder reaction[24] and recently by Tantillo to describe intramolecular (2+2)-cycloaddition reaction which involves diradical intermediates.[25]

---

[18] J.L. Banks, H.S. Beard, Y. Cao, A.E. Cho, W. Damm, R. Farid, A.K. Felts, T.A. Halgren, D.T. Mainz, J.R. Maple, R. Murphy, D.M. Philipp, M.P. Repasky, L.Y. Zhang, B.J. Berne, R.A. Friesner, E. Gallicchio, R.M. Levy, *J. Comp. Chem*. **2005**, *26*, 1752.

[19] MacroModel, Schrödinger, LLC, New York, NY, **2017.**

[20] a) C. Adamo, V. Barone, *J. Chem. Phys*., **1999**, *110*, 6158; b) S. Grimme, J. Antony, S. Ehrlich, H. Krieg, *J. Chem. Phys.* **2010**, *132*, 154104; c) W. J. Hehre, R. Ditchfield, J. A. Pople, *J. Chem. Phys*. **1972**, *56*, 2257.

[21] A. V. Marenich, C. J. Cramer, D. G. Truhlar, *J. Phys. Chem. B* **2009**, *113*, 6378.

[22] (5) Gaussian 09, Revision D.01, Frisch, M. J.; Trucks, G. W.; Schlegel, H. B.; Scuseria, G. E.; Robb, M. A.; Cheeseman, J. R.; Scalmani, G.; Barone, V.; Mennucci, B.; Petersson, G. A.; Nakatsuji, H.; Caricato, M.; Li, X.; Hratchian, H. P.; Izmaylov, A. F.; Bloino, J.; Zheng, G.; Sonnenberg, J. L.; Hada, M.; Ehara, M.; Toyota, K.; Fukuda, R.; Hasegawa, J.; Ishida, M.; Nakajima, T.; Honda, Y.; Kitao, O.; Nakai, H.; Vreven, T.; Montgomery, J. A., Jr.; Peralta, J. E.; Ogliaro, F.; Bearpark, M.; Heyd, J. J.; Brothers, E.; Kudin, K. N.; Staroverov, V. N.; Kobayashi, R.; Normand, J.; Raghavachari, K.; Rendell, A.; Burant, J. C.; Iyengar, S. S.; Tomasi, J.; Cossi, M.; Rega, N.; Millam, J. M.; Klene, M.; Knox, J. E.; Cross, J. B.; Bakken, V.; Adamo, C.; Jaramillo, J.; Gomperts, R.; Stratmann, R. E.; Yazyev, O.; Austin, A. J.; Cammi, R.; Pomelli, C.; Ochterski, J. W.; Martin, R. L.; Morokuma, K.; Zakrzewski, V. G.; Voth, G. A.; Salvador, P.; Dannenberg, J. J.; Dapprich, S.; Daniels, A. D.; Farkas, Ö.; Foresman, J. B.; Ortiz, J. V.; Cioslowski, J.; Fox, D. J. Gaussian, Inc., Wallingford CT, **2013**.

[23] a) K. Yamaguchi, F. Jensen, A. Dorigo, K. N. Houk, *Chem. Phys. Lett*. **1988**, *149*, 537; b) S. Yamanaka, T. Kawakami, H. Nagao, K. Yamaguchi, *Chem. Phys. Lett*. **1994**, *231*, 25; c) E. Goldstein, B. Beno, K. N. Houk, *J. Am. Chem. Soc.* **1996**, *118*, 6036.

[24] A. Reddy, M. Bendikov, *Chem. Commun.* **2006**, 1179.

[25] Q. Nhu, N. Nguten, D. Tantillo. *J. Org. Chem.* **2016**, *81*, 5295.

Cartesian coordinates (the most stable ($\Delta G_{298,DCM}$) conformations as computed at the (U)PBE0-D3-SMD/6-31G(d) level of theory

A’_singlet

N	0.35920	0.23124	0.58020
C	0.45806	-0.65357	1.74967
H	1.35892	-0.38738	2.30185
H	-0.43737	-0.53996	2.35864
C	0.55816	-2.03102	1.08958
H	1.44231	-2.58086	1.40861
H	-0.34388	-2.62486	1.23236
O	0.69049	-1.79956	-0.32766
C	0.57475	-0.54226	-0.66366
O	0.60915	-0.05050	-1.74767
C	0.08830	1.45096	0.55896
C	-0.18123	2.72153	0.49482
O	-2.13313	-0.77286	0.28448
C	-3.52092	-1.45626	-1.48597
C	-4.17011	-1.63626	1.07002
C	-5.01895	-1.58560	-1.78442
C	-5.62667	-1.74142	0.60486
C	-5.77638	-2.40712	-0.75387
H	-5.12011	-2.01033	-2.78995
H	-5.45650	-0.57810	-1.81837
H	-6.18368	-2.28030	1.38006
H	-6.05283	-0.72998	0.54976
H	-6.83649	-2.46038	-1.02716
H	-5.40756	-3.44019	-0.72643
N	-3.31026	-1.14370	-0.04374
C	-2.94782	-0.29900	-2.30155
H	-3.40610	0.65080	-2.00523
H	-1.86545	-0.21598	-2.18486
H	-3.16910	-0.46940	-3.36056
C	-2.76500	-2.74633	-1.82212
H	-1.72237	-2.66616	-1.49871
H	-2.77812	-2.90593	-2.90569
H	-3.21241	-3.62295	-1.34493
C	-4.07392	-0.62499	2.21027
H	-4.37220	0.37349	1.87216
H	-4.74935	-0.92969	3.01658
H	-3.05876	-0.56775	2.61056
C	-3.63824	-2.99314	1.54367
H	-3.77360	-3.77671	0.79307
H	-2.57154	-2.91885	1.77806
H	-4.17070	-3.29723	2.45133
H	0.69508	3.37377	0.52702
C	-1.50750	3.33113	0.39564
C	-2.67396	2.55215	0.36421
C	-1.59457	4.72683	0.33389
C	-3.91174	3.17187	0.27169
H	-2.60392	1.46793	0.41058
C	-2.84035	5.33899	0.24121
H	-0.68777	5.32571	0.35781
C	-3.99804	4.56466	0.21019
H	-4.81433	2.56688	0.24607
H	-2.90549	6.42220	0.19309
H	-4.96991	5.04499	0.13761
O	3.01471	-0.37617	0.46135
N	4.24284	-0.12473	0.22807
C	5.22107	-1.08074	0.81766
C	4.52594	0.77255	-0.92548
C	5.97398	1.26975	-0.84830
H	6.20352	1.77623	-1.79310
H	6.04342	2.02562	-0.05361
C	6.62905	-0.47630	0.78063
H	7.33959	-1.26970	1.04076
H	6.70271	0.28952	1.56520
C	6.97461	0.16178	-0.55570
H	7.98843	0.57664	-0.51833
H	6.97238	-0.58681	-1.35768
C	4.80969	-1.32077	2.26900
H	3.85479	-1.84879	2.33209
H	5.57529	-1.93031	2.76052
H	4.72262	-0.37288	2.81131
C	5.15243	-2.40233	0.04423
H	5.71312	-3.17374	0.58365
H	4.11079	-2.72785	-0.04474
H	5.57599	-2.31776	-0.96031
C	3.57110	1.96190	-0.82734
H	2.53931	1.65961	-1.02251
H	3.62895	2.42758	0.16285
H	3.85554	2.70877	-1.57627
C	4.25839	0.01839	-2.23317
H	4.29397	0.72304	-3.07143
H	4.99709	-0.76552	-2.42013
H	3.26285	-0.43556	-2.20922

A’_triplet

N	0.35906	0.23079	0.57989

C	0.45769	-0.65375	1.74958
H	1.35857	-0.38755	2.30176
H	-0.43777	-0.53981	2.35845
C	0.55765	-2.03137	1.08982
H	1.44160	-2.58133	1.40919
H	-0.34453	-2.62500	1.23254
O	0.69033	-1.80025	-0.32743
C	0.57466	-0.54301	-0.66375
O	0.60911	-0.05153	-1.74788
C	0.08842	1.45056	0.55838
C	-0.18067	2.72123	0.49430
O	-2.13363	-0.77211	0.28456
C	-3.52112	-1.45621	-1.48589
C	-4.17042	-1.63603	1.07005
C	-5.01912	-1.58567	-1.78443
C	-5.62697	-1.74130	0.60485
C	-5.77660	-2.40711	-0.75384
H	-5.12020	-2.01053	-2.78991
H	-5.45673	-0.57819	-1.81853
H	-6.18398	-2.28015	1.38006
H	-6.05317	-0.72987	0.54966
H	-6.83670	-2.46039	-1.02719
H	-5.40779	-3.44017	-0.72629
N	-3.31062	-1.14336	-0.04369
C	-2.94804	-0.29909	-2.30167
H	-3.40647	0.65073	-2.00568
H	-1.86569	-0.21592	-2.18488
H	-3.16917	-0.46980	-3.36066
C	-2.76512	-2.74631	-1.82172
H	-1.72247	-2.66599	-1.49839
H	-2.77828	-2.90622	-2.90524
H	-3.21243	-3.62281	-1.34423
C	-4.07433	-0.62480	2.21033
H	-4.37286	0.37363	1.87231
H	-4.74963	-0.92969	3.01669
H	-3.05915	-0.56735	2.61054
C	-3.63849	-2.99289	1.54368
H	-3.77370	-3.77644	0.79302
H	-2.57184	-2.91856	1.77822
H	-4.17105	-3.29707	2.45126
H	0.69589	3.37313	0.52642
C	-1.50670	3.33138	0.39531
C	-2.67347	2.55288	0.36393
C	-1.59320	4.72713	0.33378
C	-3.91101	3.17312	0.27170
H	-2.60385	1.46862	0.41015
C	-2.83874	5.33981	0.24139
H	-0.68615	5.32562	0.35766
C	-3.99675	4.56597	0.21043
H	-4.81386	2.56853	0.24613
H	-2.90344	6.42306	0.19345
H	-4.96843	5.04671	0.13808
O	3.01456	-0.37614	0.46163
N	4.24271	-0.12493	0.22820
C	5.22088	-1.08116	0.81749
C	4.52580	0.77253	-0.92522
C	5.97385	1.26971	-0.84795
H	6.20340	1.77636	-1.79266
H	6.04330	2.02545	-0.05313
C	6.62887	-0.47673	0.78060
H	7.33939	-1.27021	1.04054
H	6.70253	0.28889	1.56537
C	6.97446	0.16167	-0.55556
H	7.98828	0.57652	-0.51806
H	6.97226	-0.58673	-1.35771
C	4.80952	-1.32159	2.26876
H	3.85469	-1.84977	2.33172
H	5.57519	-1.93115	2.76013
H	4.72230	-0.37384	2.81130
C	5.15221	-2.40253	0.04369
H	5.71282	-3.17412	0.58294
H	4.11056	-2.72798	-0.04544
H	5.57585	-2.31773	-0.96079
C	3.57096	1.96186	-0.82692
H	2.53919	1.65960	-1.02222
H	3.62874	2.42735	0.16337
H	3.85545	2.70887	-1.57568
C	4.25826	0.01859	-2.23304
H	4.29382	0.72338	-3.07117
H	4.99698	-0.76527	-2.42014
H	3.26273	-0.43539	-2.20915

TS_A’-B_singlet

N	1.26461	-2.36887	-0.01850
C	-0.06913	-1.99744	0.47154
H	-0.15351	-2.21991	1.53485
H	-0.22895	-0.93487	0.28643
C	-0.95547	-2.87650	-0.40467
H	-1.25837	-3.80235	0.08804
H	-1.82056	-2.33585	-0.78351
O	-0.13108	-3.24119	-1.53789
C	1.14939	-3.05010	-1.31155
O	2.07510	-3.34112	-2.00175
C	2.38260	-1.99189	0.43419

C	3.58345	-1.96568	0.93475
O	2.06384	0.17254	-0.58823
C	1.35611	2.00729	0.71117
C	0.60820	1.56363	-1.78049
C	0.11088	2.89107	0.83977
C	-0.58835	2.47719	-1.49645
C	-0.29644	3.55659	-0.46592
H	0.31019	3.63188	1.62299
H	-0.72344	2.26781	1.18930
H	-0.90990	2.91003	-2.45130
H	-1.41197	1.85335	-1.12690
H	-1.19375	4.16547	-0.30493
H	0.48632	4.23981	-0.81847
N	1.22499	1.13164	-0.48942
C	1.47041	1.10901	1.93995
H	0.61667	0.42683	2.01188
H	1.47642	1.73789	2.83645
H	2.39300	0.52454	1.92489
C	2.63603	2.84139	0.58402
H	3.48861	2.18978	0.36817
H	2.82618	3.35694	1.53175
H	2.56699	3.59769	-0.20198
C	0.12430	0.30949	-2.50677
H	-0.62413	-0.22090	-1.90958
H	-0.34852	0.60873	-3.44828
H	0.95219	-0.36570	-2.73835
C	1.67634	2.26049	-2.63002
H	1.96399	3.23340	-2.22290
H	2.57078	1.63275	-2.69723
H	1.28773	2.41966	-3.64156
H	3.74783	-2.83365	1.58401
C	4.67822	-1.00584	0.80272
C	4.89198	-0.28331	-0.37892
C	5.55731	-0.85709	1.88362
C	5.96106	0.60041	-0.45917
H	4.23111	-0.42776	-1.22551
C	6.61479	0.04116	1.79953
H	5.40005	-1.43595	2.78996
C	6.81713	0.77097	0.62929
H	6.12976	1.15492	-1.37812
H	7.28698	0.16374	2.64394
H	7.64926	1.46641	0.56061
O	-2.46205	-0.15314	-0.06631
N	-3.66423	0.06894	0.30150
C	-4.68338	0.11212	-0.78497
C	-3.96489	-0.20083	1.73602
C	-5.32685	0.39414	2.11209
H	-5.59538	0.01105	3.10378
H	-5.21831	1.48317	2.20974
C	-5.99760	0.68956	-0.24990
H	-6.76732	0.52876	-1.01380
H	-5.88242	1.77633	-0.13736
C	-6.41486	0.10413	1.09024
H	-7.35934	0.55564	1.41516
H	-6.59723	-0.97473	1.00744
C	-4.14461	1.02111	-1.88756
H	-3.24652	0.60220	-2.34765
H	-4.91080	1.13782	-2.66162
H	-3.90374	2.01280	-1.48940
C	-4.87909	-1.30071	-1.34461
H	-3.91557	-1.71939	-1.65253
H	-5.33774	-1.97702	-0.61821
H	-5.52952	-1.25912	-2.22505
C	-2.87407	0.47094	2.56759
H	-1.89685	0.01723	2.38496
H	-2.81184	1.53964	2.33574
H	-3.11498	0.36341	3.63061
C	-3.92880	-1.71430	1.97855
H	-2.98884	-2.13256	1.60248
H	-3.98805	-1.91586	3.05374
H	-4.75845	-2.23478	1.49158

TS_A’-B_triplet

N	1.26513	-2.36844	-0.01746
C	-0.06830	-1.99641	0.47303
H	-0.15236	-2.21874	1.53639
H	-0.22770	-0.93377	0.28790
C	-0.95538	-2.87523	-0.40274
H	-1.25880	-3.80067	0.09040
H	-1.82020	-2.33417	-0.78164
O	-0.13143	-3.24096	-1.53593
C	1.14918	-3.05017	-1.31020
O	2.07454	-3.34175	-2.00062
C	2.38343	-1.99207	0.43487
C	3.58454	-1.96611	0.93483
O	2.06490	0.17312	-0.58761
C	1.35808	2.00814	0.71180
C	0.60834	1.56394	-1.77925
C	0.11294	2.89196	0.84120
C	-0.58832	2.47709	-1.49423
C	-0.29573	3.55693	-0.46433
H	0.31302	3.63310	1.62392
H	-0.72104	2.26888	1.19186
H	-0.91106	2.90951	-2.44887

H	-1.41136	1.85305	-1.12366
H	-1.19304	4.16565	-0.30272
H	0.48654	4.24022	-0.81783
N	1.22624	1.13234	-0.48860
C	1.47320	1.10994	1.94057
H	0.61934	0.42798	2.01337
H	1.48025	1.73891	2.83701
H	2.39561	0.52520	1.92469
C	2.63792	2.84222	0.58373
H	3.49036	2.19057	0.36741
H	2.82870	3.35790	1.53127
H	2.56838	3.59841	-0.20234
C	0.12423	0.30963	-2.50511
H	-0.62418	-0.22058	-1.90776
H	-0.34873	0.60868	-3.44661
H	0.95205	-0.36564	-2.73669
C	1.67559	2.26104	-2.62969
H	1.96351	3.23394	-2.22271
H	2.57007	1.63343	-2.69774
H	1.28607	2.42029	-3.64086
H	3.74896	-2.83383	1.58442
C	4.67951	-1.00665	0.80177
C	4.89273	-0.28465	-0.38028
C	5.55926	-0.85769	1.88210
C	5.96195	0.59881	-0.46150
H	4.23135	-0.42934	-1.22643
C	6.61690	0.04029	1.79703
H	5.40240	-1.43614	2.78877
C	6.81869	0.76960	0.62639
H	6.13023	1.15292	-1.38077
H	7.28963	0.16305	2.64098
H	7.65093	1.46484	0.55694
O	-2.46393	-0.15356	-0.06703
N	-3.66614	0.06868	0.30062
C	-4.68518	0.11131	-0.78596
C	-3.96684	-0.20044	1.73524
C	-5.32880	0.39477	2.11090
H	-5.59741	0.01228	3.10281
H	-5.22025	1.48385	2.20791
C	-5.99951	0.68890	-0.25129
H	-6.76916	0.52755	-1.01514
H	-5.88447	1.77574	-0.13939
C	-6.41678	0.10420	1.08916
H	-7.36126	0.55590	1.41380
H	-6.59917	-0.97470	1.00696
C	-4.14628	1.01985	-1.88887
H	-3.24797	0.60086	-2.34849
H	-4.91226	1.13602	-2.66321
H	-3.90567	2.01178	-1.49115
C	-4.88071	-1.30180	-1.34497
H	-3.91707	-1.72063	-1.65231
H	-5.33961	-1.97773	-0.61838
H	-5.53083	-1.26065	-2.22566
C	-2.87600	0.47170	2.56651
H	-1.89879	0.01785	2.38411
H	-2.81371	1.54028	2.33414
H	-3.11693	0.36471	3.62959
C	-3.93076	-1.71379	1.97849
H	-2.99062	-2.13214	1.60295
H	-3.99039	-1.91485	3.05375
H	-4.76017	-2.23456	1.49143

B_singlet

N	0.79410	-1.60630	-0.92424
C	1.10942	-2.64495	0.05162
H	2.19023	-2.72782	0.18237
H	0.63595	-2.43526	1.00908
C	0.49936	-3.85768	-0.63873
H	1.09099	-4.76556	-0.52238
H	-0.53245	-4.03944	-0.32236
O	0.48435	-3.50712	-2.03211
C	0.51122	-2.17371	-2.16557
O	0.34180	-1.60544	-3.21516
C	0.70160	-0.25679	-0.67574
C	-0.27357	0.50378	-1.28601
O	1.50645	0.31804	0.22005
C	3.75856	0.26206	-0.58128
C	3.08803	-0.02967	1.89547
C	5.15435	-0.26145	-0.19843
C	4.51088	-0.54086	2.16819
C	5.55066	0.05273	1.23319
H	5.86726	0.16332	-0.91479
H	5.16613	-1.35075	-0.34124
H	4.74300	-0.32618	3.21803
H	4.51513	-1.63360	2.05515
H	6.53669	-0.37430	1.45012
H	5.63956	1.13543	1.38368
N	2.81155	-0.28262	0.44743
C	3.40297	-0.35436	-1.93741
H	3.30810	-1.44246	-1.86549
H	2.47988	0.05420	-2.36190
H	4.20639	-0.12840	-2.64694
C	3.76404	1.78624	-0.74314
H	4.25132	2.31067	0.08044

H	2.74848	2.17787	-0.85094
H	4.31232	2.03738	-1.65765
C	2.11706	-0.87991	2.71584
H	2.31704	-0.72785	3.78178
H	1.07650	-0.59880	2.52784
H	2.24472	-1.94414	2.49958
C	2.93705	1.42749	2.35373
H	1.95196	1.82570	2.09610
H	3.02809	1.46013	3.44487
H	3.69622	2.09425	1.94418
H	-0.90429	-0.04150	-1.97600
C	-0.47958	1.93348	-1.24808
C	-1.35982	2.47730	-2.20863
C	0.09218	2.81499	-0.30662
C	-1.64635	3.83473	-2.23739
H	-1.80658	1.81506	-2.94646
C	-0.19697	4.17321	-0.34159
H	0.75392	2.42962	0.45802
C	-1.06500	4.69182	-1.30292
H	-2.32343	4.22633	-2.99176
H	0.25510	4.83268	0.39462
H	-1.28898	5.75489	-1.32119
O	-1.58508	-0.87204	0.38703
N	-2.74445	-0.57414	0.63835
C	-3.01351	0.26802	1.85193
C	-3.80773	-1.04906	-0.31142
C	-5.03406	-1.48588	0.50629
H	-5.84674	-1.65560	-0.20861
H	-4.80759	-2.45286	0.97440
C	-4.27801	-0.27169	2.53906
H	-4.53958	0.44053	3.32926
H	-4.02542	-1.22119	3.02898
C	-5.44021	-0.49319	1.58446
H	-6.29603	-0.89209	2.13963
H	-5.76969	0.45478	1.14329
C	-1.80892	0.15065	2.77488
H	-1.58419	-0.89448	3.00956
H	-2.04731	0.67106	3.70735
H	-0.92117	0.61512	2.33935
C	-3.17959	1.73234	1.42100
H	-3.23723	2.33503	2.33324
H	-2.31553	2.06660	0.84080
H	-4.08810	1.91014	0.84411
C	-3.24123	-2.23233	-1.08679
H	-2.44668	-1.93154	-1.77456
H	-2.85744	-3.00426	-0.41244
H	-4.05577	-2.66463	-1.67574
C	-4.14427	0.08423	-1.28959
H	-4.75317	-0.34483	-2.09169
H	-4.71221	0.89299	-0.82804
H	-3.23525	0.49848	-1.73290

B_triplet

N	0.51963	-1.17481	-1.03227
C	0.70848	-2.41434	-0.28024
H	1.73251	-2.77541	-0.37745
H	0.46881	-2.25399	0.76781
C	-0.31389	-3.32339	-0.95925
H	0.06821	-4.32607	-1.14860
H	-1.25480	-3.36362	-0.40861
O	-0.57283	-2.70491	-2.23025
C	-0.21998	-1.42621	-2.21406
O	-0.44086	-0.64293	-3.09880
C	0.83668	0.08043	-0.64822
C	-0.00433	1.16190	-0.99749
O	1.88255	0.35815	0.09932
C	3.96092	-0.50746	-0.69488
C	3.22084	-0.53144	1.78943
C	5.03718	-1.53931	-0.31665
C	4.33917	-1.54955	2.06022
C	5.52351	-1.40850	1.11759
H	5.86120	-1.42159	-1.02962
H	4.62272	-2.54563	-0.46547
H	4.64206	-1.43431	3.10740
H	3.92053	-2.55945	1.95457
H	6.26306	-2.18862	1.33080
H	6.03116	-0.44822	1.26900
N	2.88233	-0.66848	0.33714
C	3.39404	-0.89609	-2.06276
H	2.91021	-1.87710	-2.04160
H	2.68787	-0.15436	-2.45131
H	4.22125	-0.94819	-2.77821
C	4.56484	0.89500	-0.82598
H	5.18963	1.18398	0.01980
H	3.78983	1.65403	-0.96446
H	5.19841	0.91365	-1.71928
C	1.98752	-0.94625	2.59379
H	2.22036	-0.88981	3.66205
H	1.13657	-0.28316	2.40525
H	1.69596	-1.97655	2.37330
C	3.62513	0.87655	2.24546
H	2.88019	1.62150	1.95107
H	3.67653	0.88255	3.33923
H	4.60006	1.19307	1.87437

H	-0.94920	0.86158	-1.42914
C	0.17463	2.55372	-0.79790
C	-0.92555	3.37704	-1.17029
C	1.32597	3.18186	-0.25213
C	-0.87960	4.74683	-1.00103
H	-1.81057	2.90802	-1.59227
C	1.36052	4.55381	-0.09011
H	2.18008	2.58800	0.04196
C	0.26418	5.34243	-0.45880
H	-1.72957	5.35847	-1.28861
H	2.24697	5.02145	0.32826
H	0.30307	6.41972	-0.32452
O	-1.97266	-0.98879	0.06884
N	-3.15367	-0.78937	0.51017
C	-3.24289	-0.04845	1.80087
C	-4.25284	-0.90312	-0.48904
C	-5.61186	-0.92900	0.22025
H	-6.38756	-0.80328	-0.54421
H	-5.75880	-1.92420	0.66224
C	-4.66451	-0.14184	2.36381
H	-4.73927	0.56549	3.19819
H	-4.81260	-1.14694	2.78230
C	-5.73887	0.11429	1.31919
H	-6.73085	0.04965	1.78074
H	-5.64942	1.12781	0.90827
C	-2.26387	-0.70588	2.77142
H	-2.48272	-1.77392	2.87997
H	-2.35885	-0.23218	3.75422
H	-1.23196	-0.59516	2.42850
C	-2.82611	1.40748	1.56739
H	-2.74560	1.92631	2.52888
H	-1.84979	1.43945	1.07535
H	-3.54568	1.95134	0.94831
C	-4.05191	-2.21435	-1.24618
H	-3.13131	-2.20350	-1.83416
H	-4.01554	-3.05951	-0.54980
H	-4.89619	-2.36781	-1.92669
C	-4.15609	0.27192	-1.46824
H	-4.87771	0.13254	-2.28053
H	-4.37091	1.22999	-0.98570
H	-3.15383	0.31195	-1.90682

TS_B-C

N	-0.69559	-1.29072	-1.34365
C	-1.41860	-2.53071	-1.03835
H	-1.20775	-2.86823	-0.02775
H	-2.49288	-2.39352	-1.16418
C	-0.82141	-3.44672	-2.09522
H	0.08803	-3.94851	-1.75296
H	-1.53241	-4.17474	-2.48353
O	-0.46723	-2.55476	-3.16893
C	-0.27822	-1.32489	-2.70531
O	0.12914	-0.40148	-3.35522
C	-0.50915	-0.19500	-0.57815
C	0.66510	0.62703	-0.70689
O	-1.31440	0.16529	0.38480
C	-2.88153	-0.67660	1.90854
C	-3.57616	0.49102	-0.28606
C	-4.29973	-1.26091	1.99628
C	-4.96762	-0.14450	-0.11998
C	-5.35170	-0.38692	1.33151
H	-4.52526	-1.42118	3.05691
H	-4.29605	-2.24721	1.51313
H	-5.68708	0.51694	-0.61641
H	-4.98077	-1.09991	-0.66199
H	-6.32878	-0.88119	1.37809
H	-5.45895	0.56429	1.86613
N	-2.64564	-0.42766	0.45216
C	-1.88482	-1.73965	2.37048
H	-1.99146	-2.66709	1.80094
H	-0.85363	-1.38290	2.29294
H	-2.07711	-1.97692	3.42180
C	-2.71764	0.54093	2.82796
H	-1.77336	1.05992	2.63992
H	-2.69715	0.18916	3.86473
H	-3.53040	1.26317	2.74890
C	-3.21420	0.46223	-1.77275
H	-3.15027	-0.55842	-2.16066
H	-4.00116	0.97869	-2.33174
H	-2.27887	0.99151	-1.98814
C	-3.57373	1.95782	0.16238
H	-4.11625	2.12953	1.09278
H	-2.55515	2.33868	0.27627
H	-4.06298	2.55688	-0.61301
H	1.33847	0.28020	-1.48036
C	0.53518	2.09739	-0.62921
C	0.94176	2.86158	-1.73006
C	0.04856	2.74740	0.51233
C	0.85217	4.25022	-1.69520
H	1.31708	2.36168	-2.61950
C	-0.03515	4.13527	0.54453
H	-0.22929	2.16594	1.38417
C	0.36209	4.89026	-0.55887
H	1.16620	4.83148	-2.55795

H	-0.40726	4.62919	1.43818
H	0.29429	5.97446	-0.53004
O	1.45789	0.12089	0.83289
N	2.67128	-0.33421	0.80781
C	2.84401	-1.80058	0.64512
C	3.77737	0.65050	0.68836
C	5.00508	0.07875	1.42127
H	5.85106	0.74013	1.20180
H	4.81731	0.14171	2.50161
C	4.11736	-2.22547	1.39498
H	4.30220	-3.27984	1.15859
H	3.92021	-2.16464	2.47376
C	5.32225	-1.36244	1.06348
H	6.19313	-1.70337	1.63494
H	5.58864	-1.45191	0.00320
C	1.64861	-2.49618	1.28760
H	0.72643	-2.30758	0.73684
H	1.82925	-3.57596	1.29208
H	1.50833	-2.16187	2.32002
C	2.94393	-2.21806	-0.82973
H	3.85927	-1.86319	-1.30803
H	2.94415	-3.31269	-0.88383
H	2.09431	-1.85700	-1.41248
C	3.36695	1.93760	1.39901
H	2.60367	2.49090	0.85044
H	2.99108	1.72520	2.40515
H	4.25073	2.57767	1.48875
C	4.12191	0.96646	-0.77409
H	4.50143	0.09810	-1.31694
H	3.25906	1.36866	-1.31128
H	4.90030	1.73730	-0.78775

C

N	-0.65451	-1.04752	1.65609
C	-1.44517	-2.28966	1.63343
H	-2.48260	-2.08521	1.89817
H	-1.39383	-2.76118	0.65867
C	-0.71483	-3.08776	2.70301
H	-1.37836	-3.71903	3.29216
H	0.11492	-3.67211	2.29794
O	-0.16809	-2.08246	3.58001
C	-0.03146	-0.93009	2.95375
O	0.46403	0.05858	3.41285
C	-0.45325	-0.17226	0.68406
C	0.77242	0.74480	0.60928
O	-1.19455	-0.03018	-0.35837
C	-3.50348	0.38808	0.01722
C	-2.61910	-1.20661	-1.81162
C	-4.87729	-0.28815	-0.13166
C	-4.03140	-1.80641	-1.90799
C	-5.13327	-0.82886	-1.52999
H	-5.63308	0.45246	0.15413
H	-4.94037	-1.11132	0.59289
H	-4.15837	-2.17768	-2.93133
H	-4.08082	-2.67672	-1.23985
H	-6.10349	-1.33760	-1.55740
H	-5.19257	-0.00996	-2.25683
N	-2.52177	-0.66378	-0.41910
C	-3.27282	0.69089	1.49889
H	-3.26460	-0.21332	2.11349
H	-2.35039	1.25743	1.66857
H	-4.09466	1.32061	1.85393
C	-3.44371	1.72112	-0.73850
H	-2.41561	2.08007	-0.83513
H	-3.99698	2.46824	-0.16000
H	-3.89448	1.68134	-1.73028
C	-1.60229	-2.33935	-1.95070
H	-1.74982	-3.11891	-1.19732
H	-1.72830	-2.80883	-2.93160
H	-0.57425	-1.96880	-1.89512
C	-2.34479	-0.20097	-2.93742
H	-3.16121	0.50125	-3.10668
H	-1.42928	0.36783	-2.74871
H	-2.19952	-0.75967	-3.86768
H	1.38194	0.61887	1.50056
C	0.31327	2.17733	0.45559
C	-0.01096	2.91875	1.59294
C	0.18609	2.75001	-0.81100
C	-0.45921	4.23132	1.46221
H	0.09382	2.46944	2.57711
C	-0.25273	4.06548	-0.93614
H	0.44305	2.16959	-1.69180
C	-0.57980	4.80648	0.19893
H	-0.70547	4.80671	2.35053
H	-0.34163	4.51132	-1.92317
H	-0.92415	5.83227	0.09856
O	1.49686	0.28765	-0.52061
N	2.77691	-0.25218	-0.15389
C	2.84268	-1.63415	-0.69921
C	3.82081	0.73575	-0.55144
C	5.18475	0.09657	-0.24404
H	5.96017	0.79505	-0.58088
H	5.28557	0.00507	0.84616
C	4.24489	-2.18215	-0.39229

H	4.31618	-3.17956	-0.84270
H	4.33429	-2.31181	0.69537
C	5.36412	-1.27462	-0.87208
H	6.33593	-1.69461	-0.58748
H	5.36416	-1.20321	-1.96664
C	1.84228	-2.48224	0.08796
H	0.81201	-2.27166	-0.20922
H	2.02210	-3.54421	-0.11250
H	1.95726	-2.30972	1.16417
C	2.51271	-1.76029	-2.19276
H	2.31166	-2.81119	-2.43064
H	1.61803	-1.18186	-2.44199
H	3.32711	-1.42955	-2.84092
C	3.67356	1.96032	0.35507
H	2.79267	2.55638	0.10242
H	3.60972	1.66316	1.40796
H	4.54978	2.60724	0.23550
C	3.74714	1.20398	-2.00986
H	2.72510	1.49301	-2.27137
H	4.38701	2.08439	-2.13640
H	4.08607	0.44783	-2.72120

TS_C-D

N	-0.39900	-0.90791	1.84746
C	-1.24348	-2.09726	1.92068
H	-2.28748	-1.82141	2.07532
H	-1.15074	-2.67636	1.00329
C	-0.62926	-2.79860	3.12487
H	-1.36573	-3.26631	3.77725
H	0.13670	-3.52461	2.83968
O	0.01254	-1.74278	3.86687
C	0.24257	-0.69150	3.08419
O	0.86212	0.28104	3.42804
C	-0.23975	-0.17869	0.71304
C	0.95871	0.77068	0.54936
O	-0.98914	-0.31106	-0.26094
C	-3.60022	0.48132	-0.06492
C	-3.03418	-1.40614	-1.59962
C	-5.15612	0.04413	-0.15486
C	-4.59294	-1.79901	-1.68363
C	-5.48572	-0.59955	-1.47853
H	-5.67517	0.99318	0.02065
H	-5.37066	-0.63248	0.67741
H	-4.68041	-2.25432	-2.67625
H	-4.79135	-2.56604	-0.92903
H	-6.53106	-0.93494	-1.45586
H	-5.39951	0.11290	-2.30486
N	-3.05341	-0.79068	-0.32746
C	-3.35663	0.86721	1.39340
H	-3.48904	0.01361	2.06317
H	-2.34559	1.26956	1.50964
H	-4.05919	1.65328	1.68502
C	-3.32412	1.67682	-0.97647
H	-2.24965	1.77250	-1.14355
H	-3.66464	2.58014	-0.46162
H	-3.83345	1.63024	-1.93849
C	-2.27052	-2.72588	-1.51735
H	-2.54700	-3.28833	-0.62171
H	-2.49648	-3.33490	-2.39779
H	-1.19406	-2.52868	-1.50284
C	-2.60256	-0.62772	-2.84296
H	-3.31119	0.13748	-3.15726
H	-1.62839	-0.16395	-2.66935
H	-2.49842	-1.34203	-3.66515
H	1.61738	0.71110	1.41367
C	0.42143	2.17192	0.36808
C	0.05236	2.92102	1.48838
C	0.25066	2.70673	-0.90987
C	-0.48244	4.19717	1.32894
H	0.19168	2.50306	2.48233
C	-0.27564	3.98731	-1.06467
H	0.53787	2.12047	-1.77717
C	-0.64762	4.73321	0.05274
H	-0.76145	4.77676	2.20506
H	-0.39768	4.40172	-2.06210
H	-1.05978	5.73133	-0.07002
O	1.65599	0.33917	-0.60515
N	2.90451	-0.27419	-0.27153
C	2.88898	-1.65171	-0.82735
C	3.98731	0.66313	-0.67780
C	5.32294	-0.03867	-0.39254
H	6.12914	0.62143	-0.73506
H	5.43274	-0.14365	0.69564
C	4.26663	-2.27101	-0.54622
H	4.28556	-3.26613	-1.00724
H	4.36388	-2.41616	0.53887
C	5.42370	-1.41379	-1.03259
H	6.37731	-1.88492	-0.76629
H	5.41128	-1.33324	-2.12668
C	1.85113	-2.45320	-0.03757
H	0.83187	-2.18989	-0.33568
H	1.98110	-3.52267	-0.23765
H	1.97097	-2.28567	1.03886
C	2.52591	-1.74942	-2.31532

H	2.30169	-2.79328	-2.56434
H	1.63424	-1.15220	-2.53078
H	3.33082	-1.42150	-2.97633
C	3.89654	1.88927	0.23493
H	3.01575	2.49861	0.01157
H	3.86071	1.59073	1.28875
H	4.78003	2.52043	0.08746
C	3.91712	1.14597	-2.13258
H	2.90655	1.48769	-2.37492
H	4.59847	1.99426	-2.26348
H	4.20677	0.37930	-2.85461

D_1

N	0.07605	2.29862	-1.63977
C	0.30626	3.17157	-2.78578
H	0.41022	2.57478	-3.69728
H	-0.51916	3.87359	-2.90858
C	1.60453	3.84725	-2.36755
H	2.31661	3.97300	-3.18265
H	1.43421	4.80444	-1.86712
O	2.18480	2.94392	-1.40310
C	1.24897	2.11982	-0.92097
O	1.46741	1.35076	-0.01086
C	-1.13200	1.62050	-1.52217
C	-1.40358	0.83579	-0.23893
O	-1.96556	1.74526	-2.39620
C	4.23975	0.83610	1.86080
C	4.31489	-0.90395	0.03984
C	2.48923	-0.78494	1.71253
C	3.34856	-1.79741	-0.73925
C	1.80293	-1.98344	1.15976
H	2.61761	-1.15685	-1.25011
H	3.92791	-2.31332	-1.51125
H	1.48453	-2.58942	2.01792
H	0.86622	-1.60998	0.71302
N	3.61193	-0.33476	1.24603
C	5.53296	-1.68364	0.53207
H	5.24495	-2.53388	1.15777
H	6.08470	-2.06091	-0.33487
H	6.21293	-1.04938	1.10979
C	4.74064	0.24703	-0.87361
H	3.89857	0.91099	-1.08915
H	5.56394	0.83771	-0.46394
H	5.08756	-0.18602	-1.81710
H	-0.48764	0.38769	0.14315
C	-1.96368	1.83502	0.75540
C	-1.15862	2.37215	1.76097
C	-3.29855	2.23598	0.65042
C	-1.68786	3.29120	2.66480
H	-0.11850	2.06706	1.83555
C	-3.82703	3.14807	1.55911
H	-3.91866	1.82190	-0.13967
C	-3.02292	3.67776	2.56841
H	-1.05696	3.69982	3.45015
H	-4.86837	3.44878	1.47788
H	-3.43675	4.39015	3.27737
O	-2.34388	-0.17931	-0.53190
N	-1.82366	-1.47984	-0.25661
C	-2.65329	-2.06900	0.82865
C	-1.70172	-2.20117	-1.55092
C	-1.23626	-3.63050	-1.23508
H	-1.20231	-4.18934	-2.17817
H	-0.20642	-3.58474	-0.85406
C	-2.14719	-3.49955	1.06396
H	-2.78709	-3.96235	1.82520
H	-1.13388	-3.44441	1.48694
C	-2.10617	-4.33409	-0.20605
H	-1.70254	-5.33060	0.01001
H	-3.11894	-4.48687	-0.59887
C	-2.38363	-1.25466	2.09502
H	-2.81795	-1.76476	2.96221
H	-2.82641	-0.25701	2.03544
H	-1.30594	-1.14804	2.26405
C	-4.16633	-2.05461	0.57022
H	-4.48854	-2.81797	-0.14142
H	-4.69540	-2.24083	1.51192
H	-4.48010	-1.07581	0.19670
C	-0.59689	-1.51007	-2.35567
H	-0.94066	-0.55991	-2.77272
H	0.28794	-1.32852	-1.73388
H	-0.29856	-2.14658	-3.19663
C	-2.97322	-2.20823	-2.40971
H	-2.72009	-2.53003	-3.42653
H	-3.74241	-2.88662	-2.03439
H	-3.39248	-1.19980	-2.46941
C	2.61519	-2.77639	0.15556
H	4.10760	0.81406	2.94183
H	5.30668	0.82963	1.64763
H	3.78969	1.74302	1.44712
C	1.81697	-0.10216	2.85226
H	2.35577	-0.31434	3.78524
H	1.79069	0.98136	2.71481
H	0.79759	-0.47658	2.96176
H	3.32140	-3.43455	0.67299

H	1.95461	-3.41839	-0.43429

D_2

N	0.02458	2.26841	-1.13920
C	-0.90124	2.56047	-2.22549
H	-1.00496	1.69902	-2.88615
H	-1.88297	2.82693	-1.82061
C	-0.19536	3.73736	-2.88540
H	0.44760	3.42424	-3.71302
H	-0.87180	4.52555	-3.21545
O	0.64718	4.27859	-1.84939
C	0.84798	3.37186	-0.88867
O	1.60194	3.54255	0.03686
C	-0.06065	1.07858	-0.45094
C	0.96567	0.75711	0.64171
O	-0.94415	0.28752	-0.73882
C	-2.43307	-2.34389	-0.42051
C	-4.48254	-0.93421	-0.86981
C	-3.17552	-0.72622	1.21292
C	-5.18698	-0.04989	0.17496
C	-4.06971	0.48686	1.05333
H	-2.13094	-0.47730	1.39624
H	-3.53443	-1.39309	2.00551
H	-5.77647	0.72755	-0.31797
H	-5.86911	-0.66652	0.77281
H	-4.41753	0.85604	2.02106
H	-3.52197	1.29379	0.55537
N	-3.29414	-1.44221	-0.07866
C	-1.34610	-2.72103	0.52465
H	-0.90894	-3.67802	0.23223
H	-0.56469	-1.95092	0.46261
H	-1.68971	-2.77968	1.56042
C	-2.40902	-3.03881	-1.73219
H	-2.66912	-4.09288	-1.57272
H	-1.37365	-3.02503	-2.09203
H	-3.05614	-2.62325	-2.49737
C	-5.40956	-2.06317	-1.30669
H	-5.03578	-2.63519	-2.15811
H	-5.60637	-2.74959	-0.47658
H	-6.36332	-1.61629	-1.60692
C	-3.99698	-0.08144	-2.04081
H	-4.87172	0.32424	-2.55923
H	-3.37501	0.74587	-1.69145

H	-3.41242	-0.65439	-2.76484
H	1.78163	1.47795	0.62350
C	0.23482	0.81573	1.97045
C	-0.40372	2.00331	2.34549
C	0.16029	-0.29295	2.81411
C	-1.11538	2.07376	3.53898
H	-0.33444	2.88133	1.70757
C	-0.54949	-0.21849	4.01240
H	0.65720	-1.21437	2.53147
C	-1.19507	0.96041	4.37551
H	-1.60584	3.00265	3.81799
H	-0.59696	-1.08926	4.66149
H	-1.75086	1.01587	5.30774
O	1.48282	-0.53992	0.40666
N	2.78916	-0.47712	-0.18337
C	3.77699	-0.87622	0.85514
C	2.75275	-1.22737	-1.46461
C	4.17286	-1.17891	-2.05066
H	4.17364	-1.76947	-2.97511
H	4.39309	-0.13956	-2.33142
C	5.16352	-0.84263	0.19247
H	5.89777	-1.18510	0.93192
H	5.40726	0.20252	-0.04343
C	5.23989	-1.66477	-1.08343
H	6.23291	-1.56354	-1.53756
H	5.10583	-2.73122	-0.86399
C	3.76238	0.19525	1.94891
H	2.84286	0.16431	2.54095
H	4.60013	0.02520	2.63457
H	3.87222	1.19726	1.51990
C	3.51153	-2.23696	1.51535
H	2.45445	-2.33691	1.77743
H	3.78899	-3.08346	0.88366
H	4.09492	-2.31298	2.43994
C	1.82900	-0.47311	-2.42351
H	0.77671	-0.62315	-2.16974
H	2.05091	0.60025	-2.41372
H	1.98033	-0.84415	-3.44359
C	2.24707	-2.67453	-1.36626
H	1.98043	-3.03241	-2.36774
H	2.98984	-3.36620	-0.96254
H	1.35060	-2.72689	-0.74160
